# Oxidative Functionalization of Trinor-18α-olean-17(22)-ene Derivatives. Annulation of the E-Ring by an Intramolecular Aldol Reaction

**DOI:** 10.1021/acs.joc.1c00697

**Published:** 2021-05-25

**Authors:** Kinga Kuczynska, Jarosław Jaźwiński, Zbigniew Pakulski, Piotr Cmoch, Roman Luboradzki

**Affiliations:** † Institute of Organic Chemistry, Polish Academy of Sciences, Kasprzaka 44/52, 01-224 Warsaw, Poland; ‡ Institute of Physical Chemistry, Polish Academy of Sciences, Kasprzaka 44/52, 01-224 Warsaw, Poland

## Abstract

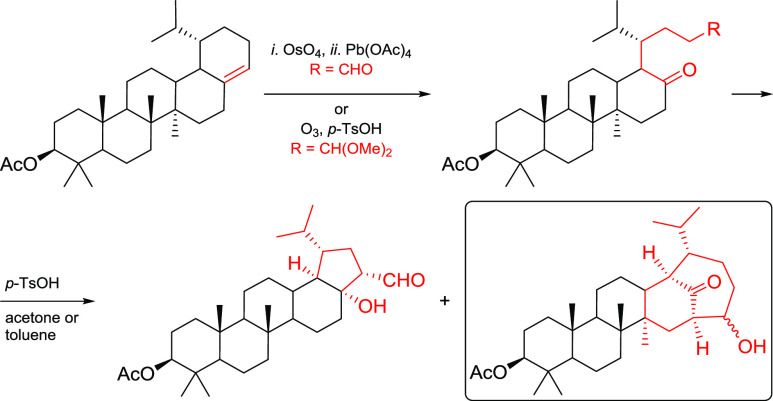

*cis*-Dihydroxylation of trinor-18α-olean-17(22)-ene **2** with osmium tetroxide led to diol **9**. Its cleavage with lead tetraacetate gave tetracyclic ketoaldehyde **10**. By comparison, the ozonation of trinor-18α-olean-17(22)-ene **2** in the presence of *p*-toluenesulfonic acid gave the corresponding ketoacetal **12**. Both products were subjected to an intramolecular aldol reaction under the acidic conditions and yielded unusual triterpenes bearing a bicyclo[4.3.1]decane fragment (**22**). Further manipulation of the protective groups afforded compounds useful in triterpene synthesis, especially in the preparation of potentially biologically active saponins based on a tetracyclic terpene core.

## Introduction

**Scheme 1:**
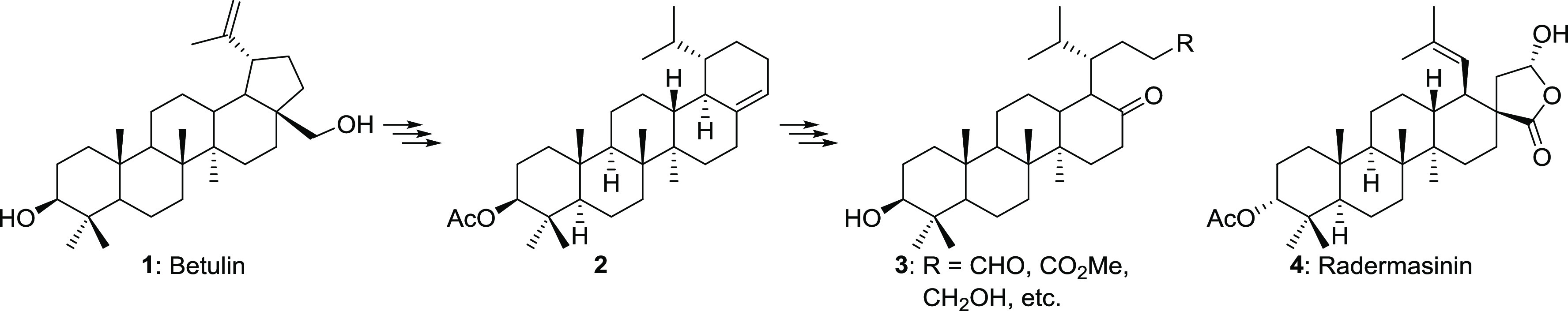
Proposed Transformation of Betulin

Birch is a hardwood tree of the genus *Betula* widespread in the Northern Hemisphere, particularly in areas of temperate and boreal climates. Its wood has had important historical and cultural significance since ancient times.^1,2^ Nowadays, birch wood is widely used in the paper industry. The outer part of birch bark (the waste generated during paper production) is an extremely rich source of lupane-type triterpenes, mostly betulin (**1**, Scheme 1), which are isolated in appreciable amounts (up to 30% of dry mass).^3,4^ Betulin has interesting biological properties, including antiviral and antitumor activities.^5−8^ Lupane triterpenoids have also been used as intermediates in the synthesis of triterpenoids employed for biological studies.^9−18^

In the pursuit of new, flexible starting materials for the functionalization of the lupane core,^19−22^ we guessed that the easily available 3β-*O*-acetyl-trinor-18α-olean-17(22)-ene (**2**)^23^ is a candidate for further transformation (Scheme 1). Special interests for us were the tetracyclic compounds with the general formula **3**, which can be obtained by oxidative cleavage of the double bond in **2**. They are structural analogues of radermasinin (**4**), a cytotoxic triterpene lactone isolated from *Radermachia sinica*.^24^

In the present paper, we report on a simple and high yield conversion of the olean-17(22)-ene scaffold by an oxidative functionalization of the double bond. The obtained derivatives may be used as starting materials for the synthesis of saponins and more complex triterpene derivatives.

## Results and Discussion

**Scheme 2:**
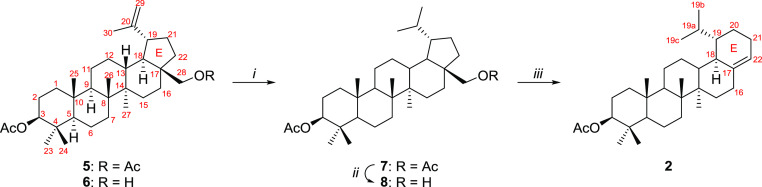
Reagents and conditions: *i*. 10% Pd/C, H_2_ (7 bar, quantitatively); *ii*. *i*-PrOH, Al(*i*-PrO)_3_, reflux, 75%; *iii*. POCl_3_, pyridine (98%).

Transformation of betulin diacetate (**5**) into 3β-*O*-acetyl-19α-isopropyl-28,29,30-trinor-18α-olean-17(22)-ene (**2**) was realized according to the modified literature methods. The key compound, 3β-*O*-acetyl-dihydrobetulin (**8**) can be prepared by several methods summarized in Scheme 2. Selective deacetylation of **5** gave monoacetate **6**.^25^ Hydrogenation of **5** or **6** yielded dihydrobetulin derivatives **7** or **8**, respectively. Similarly, selective deacetylation of **7** gave monoacetate **8**. Treatment of **8** with POCl_3_ in pyridine caused the rearrangement and an expansion of the E-ring in olean-17(22)-ene core (**2**) almost quantitatively.

**Scheme 3:**
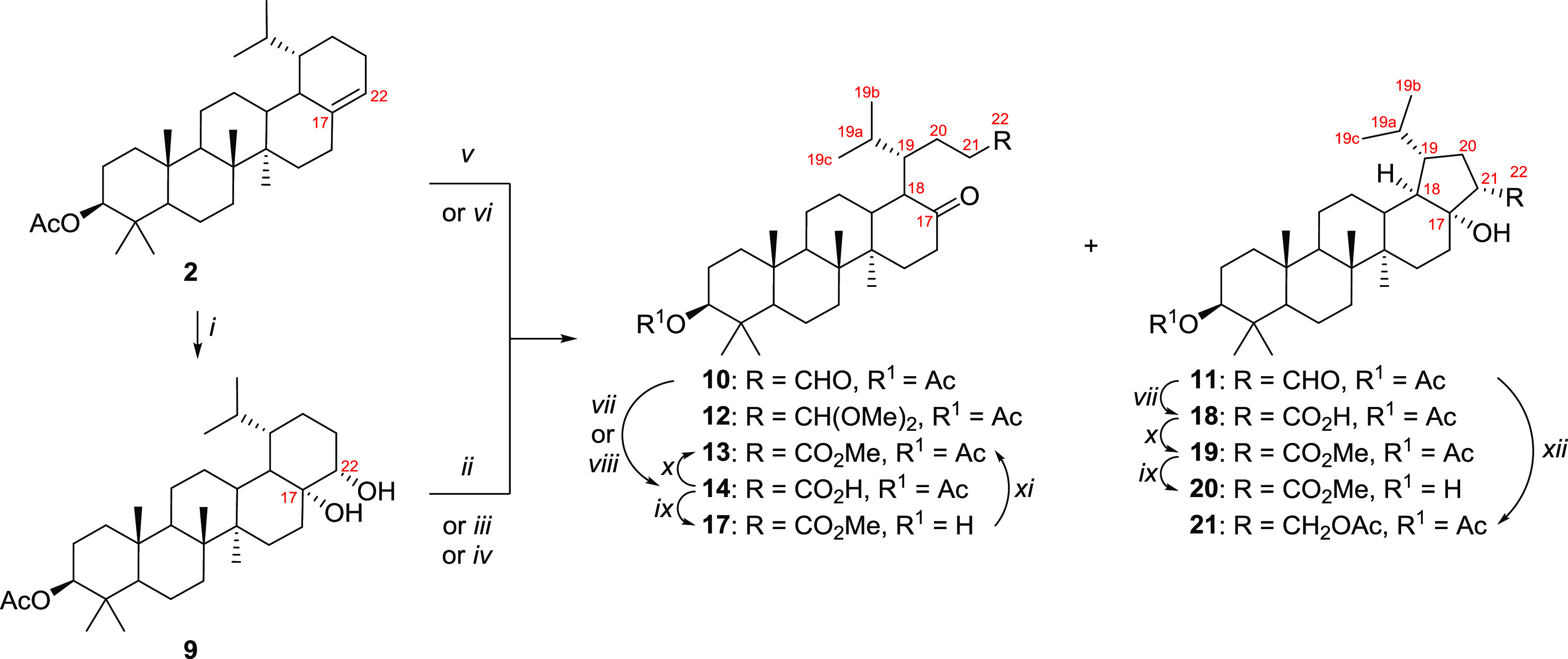
Reagents and conditions: *i*. OsO_4_, pyridine (80%); *ii*. NaIO_4_ supported on SiO_2_ (27%); *iii*. Pb(OAc)_4_, pyridine (78% of **10**, 7% of **11**); *iv*. Pb(OAc)_4_, benzene (90% of **10**); *v*. O_3_, methanol, CH_2_Cl_2_ (45–59% of **12**, 15–21% of **11**, 3–7% of **13**, and 11–15% of **14**); *vi*. O_3_, *p*-TsOH, methanol, CH_2_Cl_2_ (71% of **12**); *vii*. NaClO_2_, amylene; *viii*. Jones reagent, acetone; *ix*. MeOH, HCl (75% of **17** after two steps; 98% of **20**); *x*. MeI, K_2_CO_3_, DMF (75% of **13** after two steps; 80% of **19** after two steps); *xi*. Ac_2_O, Py (95%); *xii*. (a) LiAlH_4_, (b) Ac_2_O, Py (79% after two steps).

**Figure 1:**
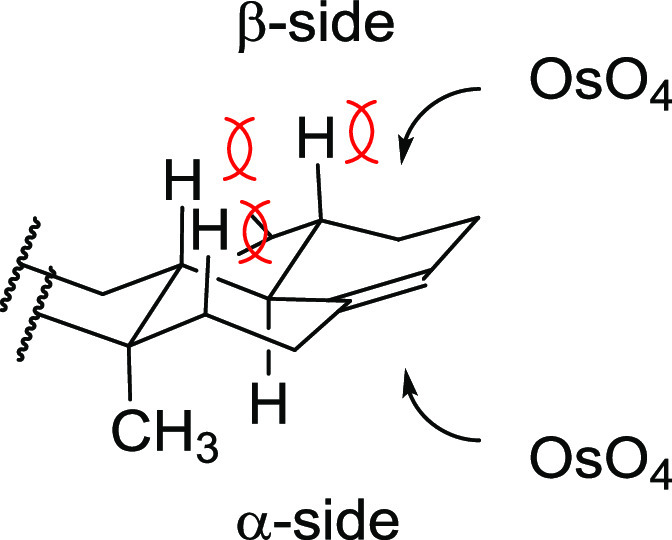
Steric interactions influencing the OsO_4_ attack.

In the first attempt, the dihydroxylation of **2** with an excess of OsO_4_ in pyridine afforded *cis*-diol **9** in high yield (80%) as a single diastereoisomer; the complete oxidation of the double bond took 7 days (Scheme 3). Catalytic dihydroxylation of **2** in catalytic version (OsO_4_/NMO) gave no product.^26,27^ According to the well-known mechanism of dihydroxylation with OsO_4_, only the *cis-*diol was formed. We expected that the steric interaction of D- and E-ring protons precluded formation of 17β,22β-diol (Figure 1). By a comparison, no steric interaction influenced the OsO_4_ attack from the α-side. The 17α,22α configuration of the new stereogenic centers in **9** was determined by the NMR experiments (see Supporting Information).

Then, we studied the oxidative cleavage of the 1,2-diol group in **9**, which, as we expected, should afford ketoaldehyde **10** as a main product. Treatment of **9** with NaIO_4_ on silica gel^28^ for 7 days, gave a single product (Scheme 3). Despite a prolonged time of the reaction, most of the starting material (53%) was recovered. In the ^1^H NMR spectra of the product, a doublet of the −CHO proton was detected at δ = 9.81 ppm. Unexpectedly, only one signal of the carbonyl group at δ = 206.5 ppm, belonging to the aldehyde moiety, was observed in the ^13^C NMR spectra. Analysis of HMBC and NOE correlations suggested that a pentacyclic product **11** was obtained (in 27% yield) instead of the expected tetracyclic **10**. Therefore, in the next attempt, we used lead tetraacetate in pyridine as an oxidizer. At room temperature, two products were obtained. In the ^1^H NMR spectrum of the main product, a typical multiplet of the −CHO proton was observed at δ = 9.75 ppm, whereas both expected signals of the carbonyl groups belonging to the ketone (δ = 202.4 ppm) and aldehyde groups (δ = 213.7 ppm) were present in the ^13^C NMR spectra. Further analysis of HMBC and NOE correlations confirmed that, in this case, compound **10** was isolated as the main product (78%), whereas **11** (7%) was identified as the minor product. Presumably, the pentacyclic compound **11** was formed in an intramolecular pyridine catalyzed aldol reaction as the basic component of this reaction. To confirm this assumption, we kept aldehyde **10** in pyridine at 50 °C for 12 h. However, no cyclization was observed. Apparently, the mechanism of this reaction is more complex and requires the presence of a metal ion. To avoid formation of **11**, we have tested benzene as a neutral solvent. As a result, tetracyclic aldehyde **10** was obtained as a sole product in 90% yield. No traces of **11** were detected in the reaction mixture. Both products (**10** and **11**) have limited shelf life.

Further optimization was focused on excluding the use of toxic OsO_4_. With alkene **2** in hand, we tried to run ozonation of the C17(22) double bond to prepare **10**. Because of the low solubility of **2** at −78 °C, we used a dichloromethane–methanol mixture as a solvent system to provide sufficient solubility of the starting material. Under these conditions, the ozonation of **2** was fast; however, a mixture of products was obtained (Scheme 3).^29,30^ Careful separation of the reaction mixture and analysis of the isolated products revealed that four compounds were obtained in this reaction. It must be noted that the composition of the mixture and the yields of the products changed significantly from batch to batch. Usually, acetal **12** (45–59%) and pentacyclic aldehyde **11** (15–21%) were isolated as the main products. In some cases, **13** (3–7%) and **14** (11–15%) were also isolated as byproducts. Surprisingly, the expected aldehyde **10** was not formed during the ozonation of **2**.

**Scheme 4:**
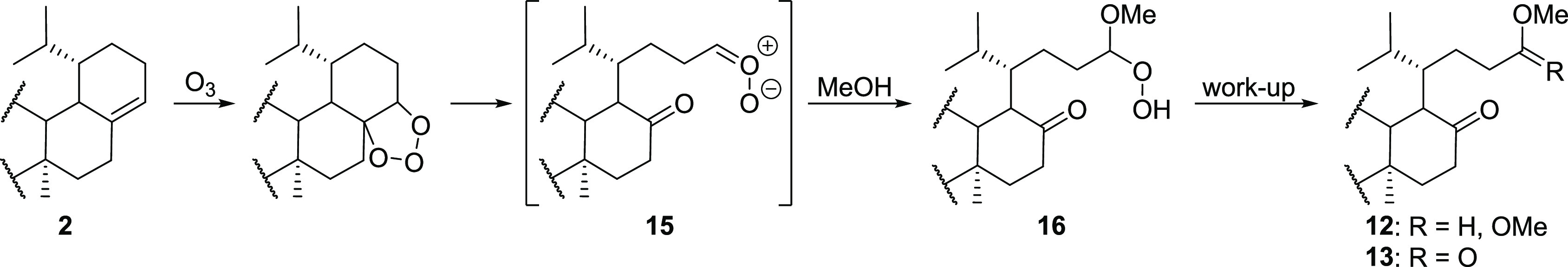
Tentative Mechanism of the Formation of Acetal and Ester

The formation of acetals and esters is rather unexpected during the ozonolysis under neutral conditions. However, in his seminal publication, Schreiber has shown^31^ that the incorporation of an acid or base during the ozonolysis of cycloalkenes, followed by a reductive workup, promoted a formation of differently substituted products, including acetals and esters, in high yields.^32,33^ As suggested, such products were formed by the addition of methanol, a participating solvent, to the carbonyl oxide **15**, reactive intermediate in the Criegee mechanism,^34^ and subsequent transformation of α-alkoxy hydroperoxide **16** during workup (Scheme 4). It is interesting that, in the case of ozonation of **2**, acetal **12** and ester **13** were formed in the absence of a catalyst. We supposed that the unpredictability of the ozonation of **2** and fluctuation in the product proportions were caused by an uncontrolled decomposition of α-alkoxy hydroperoxide **16** under neutral conditions. To confirm the above assumption, we repeated this reaction under the Schreiber’s conditions. The ozonation of **2** in the presence of NaHCO_3_ caused decomposition of the starting material. By comparison, the reaction performed in the presence of *p-*TsOH (approximately 10% w/w) afforded acetal **12** as a sole product in high yield (71%, Scheme 3).

All compounds prepared above (**10**–**12**) are valuable starting materials in the synthesis of differently substituted analogues. Therefore, in the next step, we have tested their reactivity and scope of possible transformations. We were especially interested in the synthesis of compounds bearing the free −OH groups, starting materials for the preparation of saponins (triterpene glycosides).^35^ Then, we initially converted aldehyde **10** into acid **14** by its oxidation using a procedure developed by Clive.^36^ The same reaction can be performed using the Jones reagent. Acid **14** was then transformed into methyl ester by treatment with acidic methanol which afforded **17**, bearing a free 3β-OH group (75% yield after two steps). Moreover, treatment of **14** with methyl iodide in the presence of potassium carbonate afforded fully protected ester **13** in 75% yield. The same compound **13** was obtained by acetylation of **17** under the standard conditions in 95% yield.

**Figure 2:**
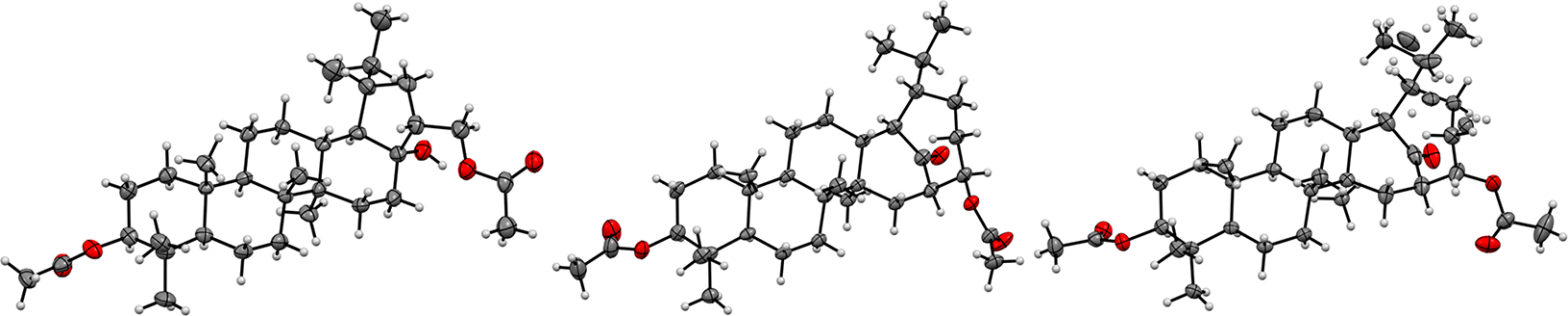
X-ray structure of **21** (left), **23** (middle), and **24** (right). Contour probability level: 50%.

Similar oxidation of **11** to free acid **18** with the Clive’s method, followed by esterification with methyl iodide, gave ester **19** (80% yield after two steps). Treatment of **19** with acidic methanol afforded ester **20** with the free 3-OH group (98%). The reduction of **11** with LiAlH_4_, followed by acetylation of the crude reaction mixture, yielded diacetate **21** (79% after two steps). Its structure was confirmed by a single-crystal X-ray analysis (Figure 2).

**Scheme 5:**
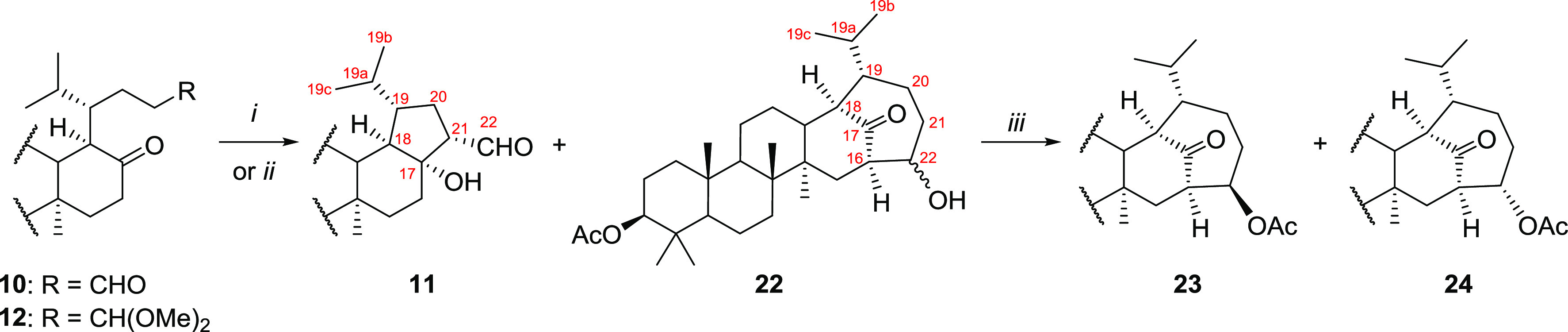
Reagents and conditions: *i*. *p*-TsOH, acetone (**10** → 20% of **11** and 56% of **22**; **12** → 18% of **11** and 59% of **22**); *ii*. *p*-TsOH, toluene (**10** → 74% of **22**; **12** → 50% of **22**); *iii*. Ac_2_O, pyridine.

Finally, we have attempted the hydrolysis of acetal **12** into aldehyde **10** by a treatment with *p*-TsOH in acetone. This reaction required high loading of acid (at least 40% w/w) for a complete transformation. Column chromatography of the reaction mixture gave two fractions. The first contained a compound which was identified as **11** (18%, Scheme 5). The second fraction was composed of an inseparable mixture of two epimeric compounds (**22**, 56%). They were chromatographically separated after acetylation under standard conditions. On the basis of the analysis of the NMR spectra, we proposed their structures as diacetates **23** (46%, after two steps) and **24** (28%, after two steps). Both structures were confirmed by single-crystal X-ray analysis (Figure 2). Similarly, treatment of aldehyde **10** with *p*-TsOH in acetone afforded the same products in an identical ratio. Notably, the subjecting of **12** to acidic conditions led to the hydrolysis of the acetal function and the formation of aldehyde **10**, which immediately cyclized by intramolecular aldol reaction,^37^ affording cyclic products **11** and **22**. When acetone was replaced by toluene, the cyclization of **10** was highly selective toward **22** (74%). Similarly, cyclization of acetal **12** in toluene solution gave the epimers **22**, but in lower yield (50%). In both cases, only traces of **11** were detected. Neither equilibration nor retro-aldol reactions were observed when compounds **11** and **22** were subjected to acidic conditions.

Compounds **22**–**24** belong to modified triterpenes with an unusual bicyclo[4.3.1]decane framework.^38−43^ To the best of our knowledge, compounds bearing the bicyclo[4.3.1]decane motif in their structure have never been synthesized by an intramolecular aldol reaction. This methodology was, however, used for the preparation of derivatives with the bicyclo[3.3.1]nonane fragment,^44−46^ found in some natural compounds.^47−50^

**Scheme 6:**
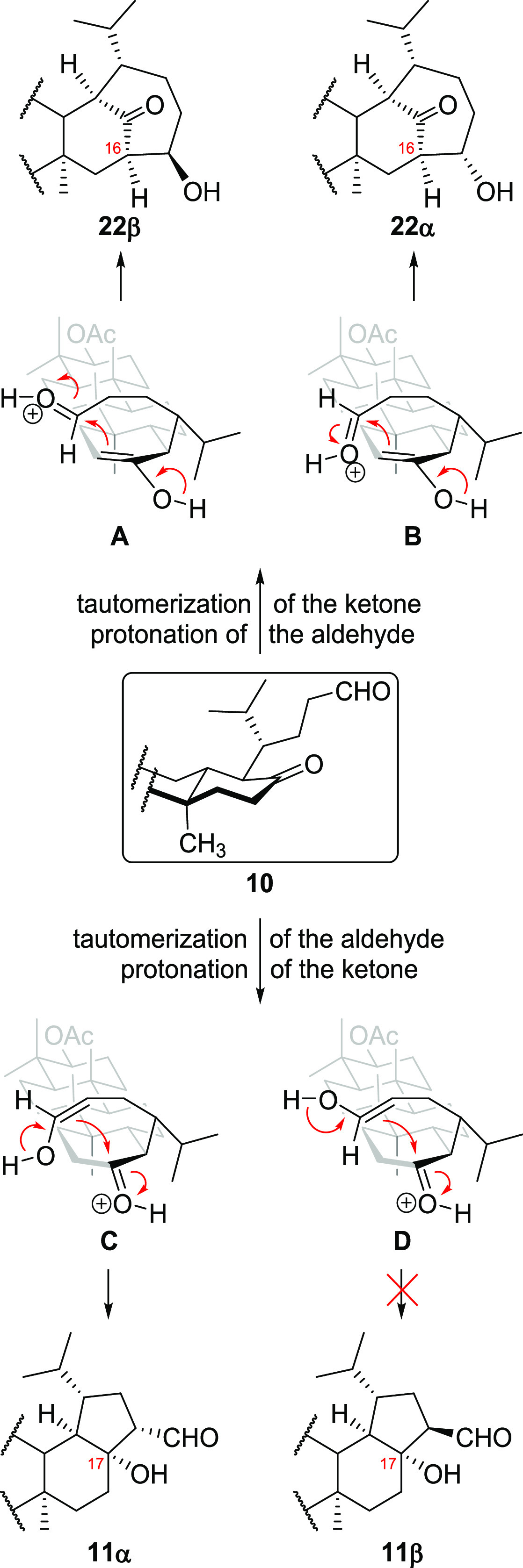
Tentative Reaction Mechanism

The possible reaction mechanism is presented in Scheme 6. When an aldehyde’s carbonyl group is protonated (structures **A** and **B**), cyclization leads to product **22** having a seven-membered ring. Protonation of the ketone’s carbonyl group (structures **C** and **D**) should result in the formation of five-membered ring products. In this case, however, compound **11α** was formed selectively. Probably, the formation of **11β** is precluded by steric interactions of the aldehyde group with protons of the ring D and the angular methyl group at the C8. Stereochemistry of the newly generated stereogenic center at C16 (in **22**) and C17 (in **11**) was determined by the structure of aldehyde **10**; formation of the new carbon–carbon bond is possible only from the β-side of the D-ring.

## DFT Calculations

**Figure 3:**
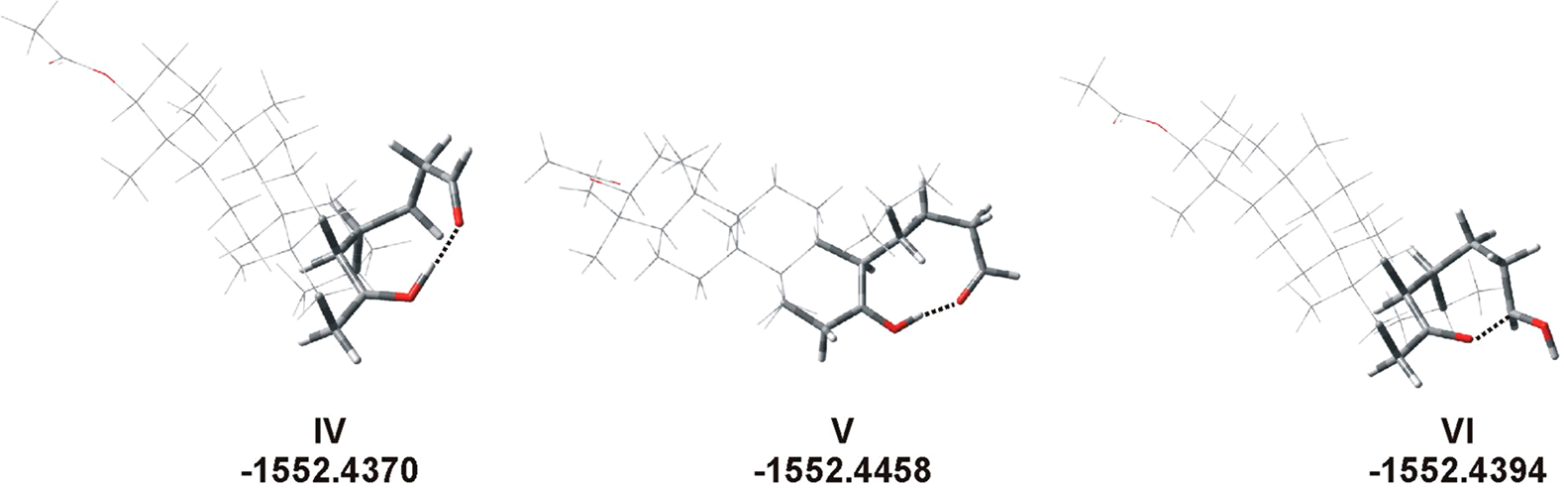
Rotamers stabilized by hydrogen bonds (dotted lines) or the O···C interaction. The bond lengths are as follows: 1.032 and 1.514 Å (O···H, **IV**), 1.032 and 1.555 Å (O···H, **V**), and 1.591 Å (=O···CH(OH^+^), **VI**).

Initially, 18 structures of **10** differing in the arrangement of the side chains were considered, while the remaining part of the molecules were unchanged. The analysis of energy revealed that only three structures have a significant population (0.22, 0.13, and 0.48 molar fractions, Figure 2S, rotamers **I**, **II**, and **III**), having an aldehyde group far from the ketone fragment. In the next step, the structures of four tautomers derived from **III** (i.e., from the low-energy structure) were optimized, and their molecular energies were estimated (Figure 3S). An analysis of the molecular energies of **III** and its tautomers together with the assumption of equilibrium between species indicated a negligible molar fraction of tautomeric forms.

**Figure 4:**
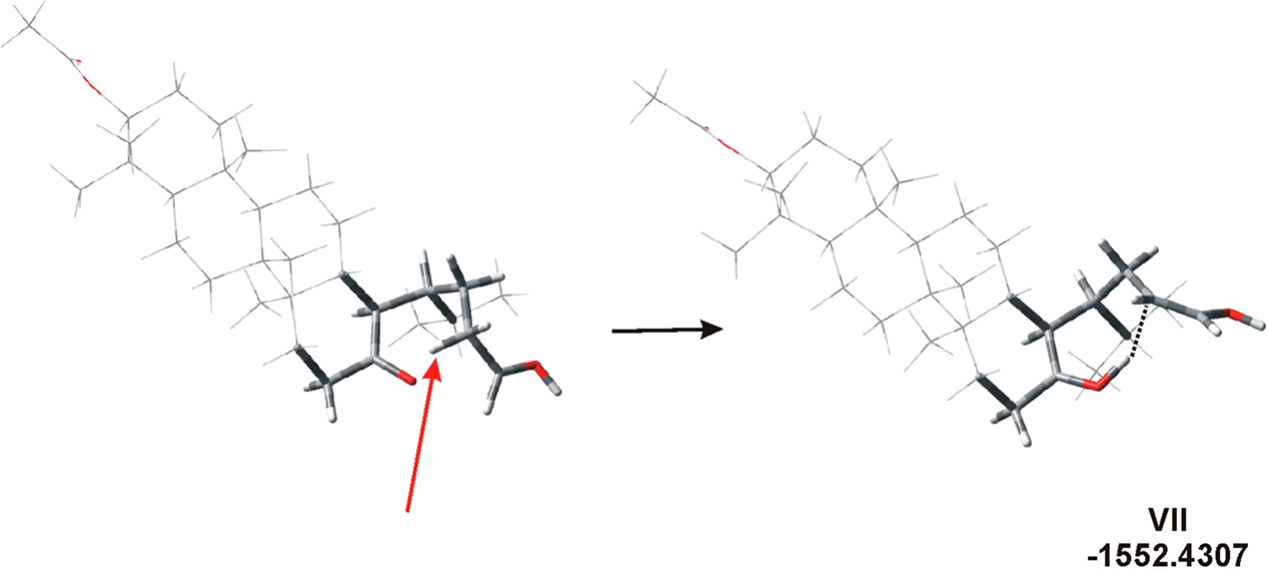
Formation of enol during structure optimization. The H atom from CH_2_ (red arrow) of the protonated aldehyde moved to the C=O group, forming the rotamer **VII** (enol) stabilized by OH···–CH= interaction (dotted line, *d*_OH_ 1.022 Å, *d*_HC_ 1.795 Å).

**Figure 5:**
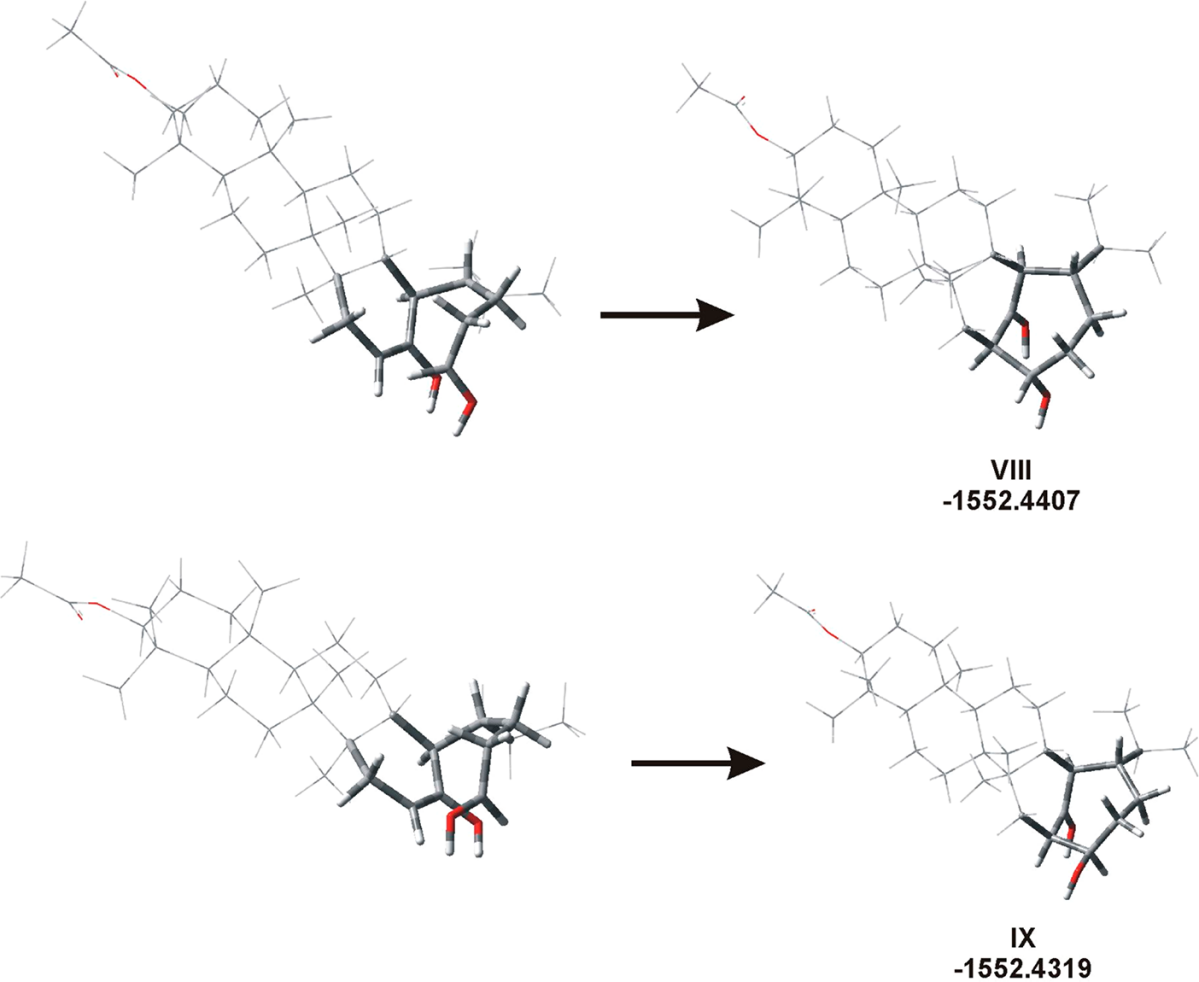
Transformation of **10** (enole form) to cyclic product **22**. Depending on the orientation of the CH(=OH^+^) group, two configurations of the CH(OH) center have been obtained.

In the third step, calculations were performed for the protonated compounds. All input structures (cations) were constructed starting from the most stable rotamer **III** and related tautomers. A proton was located at either the ketone or aldehyde groups (Figure 4S). The structure with H^+^ at the ketone group appeared to be the most stable. However, the most interesting results were obtained starting from formally unfavorable rotamers. Optimization resulted in structures stabilized by hydrogen bonds (HB) and/or other weak interactions, such as C···O, CH···O, and OH···C (Figure 3). In one case, we observed the transformation of aldehyde to enol, forced by the transfer of H (Figure 4). Twice, the ring closure occurred during optimization (Figure 5).

**Figure 6:**
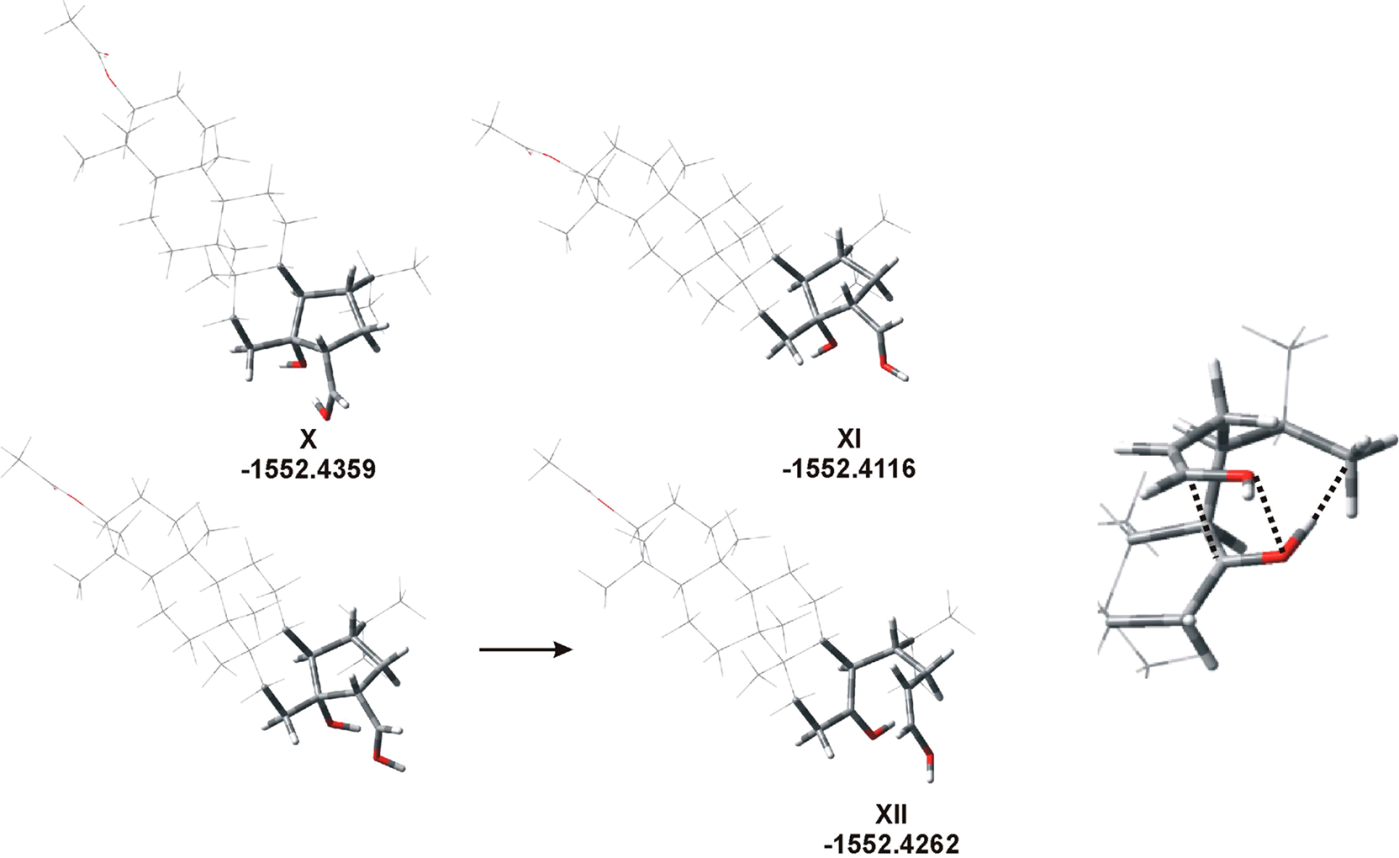
Two structures of **11** with (**X**) and without (**XI**) hydrogen bonds (top). The structure of **11** with different hydrogen bond patterns transformed to linear isomer **XII** during optimization (bottom). At right: linear structure is stabilized by various intramolecular weak interactions, O···O, C···C, and OH···σ*(CH_3_).

The last series of calculations concerned the analysis of the hydrogen bonds expected for **11**. Three initial structures differing in the arrangements of the OH bonds were considered (Figure 6), the first with the aldehyde group CH(=OH^+^) involved in HB formation, the second structure without HBs, and the third one HB formed by the ketone group (C=OH^+^). Surprisingly, an open chain product has been obtained during optimization in the last case, with molecular energy lower than cyclic **XI** without HB. This “linear” form was stabilized by the C···C, O···O, and OH···C interactions. The optimization process appeared to be reversible; cyclic **11** with HB has been obtained during optimization when a “linear” reagent with the right OH bonds pattern was used as the input structure.

Concluding, the first calculations revealed that rotamers of **10** having the CHO and CO functionalities far from one another seem to be preferential ones. However, the optimization and examination of seemingly disadvantageous rotamers of protonated **10** resulted in some structures having both C=O groups close to each other. Analysis by Atom-In-Molecule (AIM) methodology shows that these rotamers are stabilized by numerous weak interactions, such as OH···O, CH···O, and C···HO hydrogen bonds, and/or interactions between C and O atoms. Weak interactions preorganize **10**, enabling the reacting fragments to come closer, and facilitating certain reactions. In particular, the ring closure leads to **11** or **22**, depending on the conformation of the reagent. The results of the calculations are in agreement with the proposed reaction mechanism.

## Conclusions

The presented results clearly show that the oxidative functionalization of the double bond of trinor-18α-olean-17(22)-ene derivatives led to synthetically useful products. The proper choice of oxidants and reaction conditions gave highly functionalized tetracyclic triterpenes and unusual products with the bicyclo[4.3.1]decane fragment. In all cases, the key compounds were obtained as the major products. Prepared derivatives are convenient starting materials for further synthesis of tetracyclic terpene analogues, especially the synthesis of saponins. The most interesting achievement is the synthesis of triterpenes bearing a bicyclo[4.3.1]decane fragment. To our best knowledge, this is the first observation of an intramolecular aldol reaction leading to this unusual structure.^51^ As with recently reported bicyclo[3.3.1]nonane derivatives,^52^ the bicyclo[4.3.1]decane derivatives may be considered as precursors of chiral macrocycles and highly sterically hindered ligands.

## Experimental Section

### General Procedures

Silica gel HF_254_ and Silica gel 230–400 mesh (E. Merck) were used for TLC and column chromatography, respectively. ^1^H and ^13^C{^1^H} NMR spectra were recorded at 298 K with Varian NMR-vnmrs600 or vnmrs500 spectrometers, using standard experimental conditions and Varian software (ChemPack 4.1). Configurational assignments were based on the NMR measurements generated using two-dimensional techniques like COSY and ^1^H–^13^C gradient selected HSQC (g-HSQC), as well as ^1^H–^13^C gradient selected HMBC (g-HMBC). Internal TMS was used as the ^1^H and ^13^C NMR chemical shift standard. *J* values are given in Hertz. High-resolution mass spectra (HRMS ESI) were acquired with Mariner and MaldiSYNAPT G2-S HDMS (Waters) mass spectrometers. Optical rotations were measured with a Jasco P-2000 automatic polarimeter. Single crystal X-ray diffraction measurements were carried out on an Agilent Supernova diffractometer at 100 K with monochromated Cu Kα radiation (1.54184 Å). The structures of compounds **21**, **23**, and **24** were determined on crystals prepared in a chloroform/methanol solvent system by slow evaporation at room temperature.

All DFT calculations have been performed using the Gaussian program suite.^53^ Molecular energies (a.u.) have been estimated at the B3LYP/6-311++G(2d,p) level of theory, using B3LYP/6-31G(2d,p) optimized structures. All calculations were performed assuming isolated molecules. The protonated compounds were calculated as cations. Topological analysis: detection of the weak interactions, hydrogen bonds, and bond critical points was performed by the AIMAll program package.^54^ Some preliminary calculations were performed using simplified structures (Figure 1S) to save computational time; then the results were used to plan the calculations for full molecules **10**, **11**, and **22**. The population of a series of rotamers was estimated on the basis of molecular energy, using the Boltzmann distribution. The details of the calculations, some key figures as well as atomic coordinates are enclosed in the Supporting Information.

### 20,29-Dihydrobetulin 3β,28-di-*O*-acetate (**7**)

Betulin diacetate (**5**) was converted to dihydrobetulin diacetate (**7**) by a modified procedure of Lehn.^55^ To a solution of betulin diacetate (**5**, 5.27 g, 10.00 mmol) in THF (70 mL) and methanol (70 mL) was added 10% Pd/C (300 mg), and the mixture was hydrogenated under 7 bar of hydrogen for 48 h. Then, the whole mixture was filtered through a short silica pad (hexane–ethyl acetate–methanol, 5:3:1 as an eluent) to afford dihydrobetulin diacetate (**7**, 5.25 g, quant) as a white solid.^56^ No further purification was necessary. ^1^H NMR (600 MHz, CDCl_3_) δ: 4.48 (dd, 1 H, *J* 5.6 and 10.8 Hz, H-3), 4.24 (dd, 1 H, *J* 1.8 and 11.2 Hz, H-28), 3.82 (d, 1 H, *J* 10.9 Hz, H-28), 2.06 (s, 3 H, COCH_3_), 2.04 (s, 3 H, COCH_3_), 1.17–1.88 (m, 23 H), 0.87–1.03 (m, 2 H), 1.04 (s, 3 H, CH_3_), 0.95 (s, 3 H, CH_3_), 0.86 (s, 3 H, CH_3_), 0.85 (s, 3 H, CH_3_), 0.84 (s, 3 H, CH_3_), 0.84 (d, 1 H, *J* 6.8 Hz, CH_3_), 0.78–0.80 (m, 1 H, H-5), 0.77 (d, 1 H, *J* 6.8 Hz, CH_3_). ^13^C{^1^H} NMR (150 MHz, CDCl_3_) δ: 171.6 (C=O), 171.0 (C=O), 80.9 (C-3), 62.8 (CH_2_), 55.3, 49.9, 48.1, 46.4 (C), 44.5, 42.8 (C), 40.9 (C), 38.3 (CH_2_), 37.8 (C), 37.1, 37.0 (C), 34.6 (CH_2_), 34.2 (CH_2_), 29.8 (CH_2_), 29.4, 27.9, 26.9 (CH_2_), 26.8 (CH_2_), 23.7 (CH_2_), 22.9, 21.6 (CH_2_), 21.3, 21.0, 20.8 (CH_2_), 18.2 (CH_2_), 16.5, 16.1, 16.0, 14.9, 14.6.

### 20,29-Dihydrobetulin 3β-*O*-acetate (**8**)

#### Method A

To a solution of betulin 3β-*O*-acetate (**6**, 3.15 g, 6.50 mmol) in THF (35 mL) and methanol (55 mL) was added 10% Pd/C (230 mg), and the mixture was hydrogenated under 7 bar of hydrogen for 3 days. Then, the whole mixture was filtered through a syringe filter to remove the catalyst and evaporated to dryness under reduced pressure. No further purification was necessary. Dihydrobetulin 3β-acetate (**8**) was obtained quantitatively as a white solid.^56^

#### Method B

Dihydrobetulin 3β-*O*-acetate (**8**) was prepared from dihydrobetulin 3β,28-di-*O*-acetate (**7**) by selective deacetylation according to a modified procedure of Thibeault.^25^ A mixture of dihydrobetulin 3β,28-di-*O*-acetate (**7**, 10.00 g, 18.91 mmol), Al(*i*-OPr)_3_ (11.65 g, 57.04 mmol), and *i*-PrOH (300 mL) was stirred under reflux for 24 h. The crude mixture was concentrated under reduced pressure and water (300 mL) was added. The suspension was slightly acidified with 2 M HCl and extracted with chloroform (3 × 100 mL). The combined organic layers were washed with saturated NaHCO_3_ (20 mL) and concentrated under reduced pressure. Column chromatography of the residue (hexane–ethyl acetate, 15:1 to 10:1) afforded 6.86 g (75%) of **8** as a white solid. ^1^H NMR (500 MHz, CDCl_3_) δ: 4.48 (dd, 1 H, *J* 5.3 and 11.1 Hz, H-3), 3.77 (bd, 1 H, *J* 10.9 Hz, H-28), 3.30 (bd, 1 H, *J* 10.9 Hz, H-28), 2.04 (s, 3 H, CH_3_), 0.97–1.97 (m, 26 H), 1.03 (s, 3 H, CH_3_), 0.95 (s, 3 H, CH_3_), 0.86 (s, 3 H, CH_3_), 0.83–0.85 (m, 9 H, 3 x CH_3_), 0.79–0.82 (m, 1 H), 0.77 (d, 3 H, *J* 6.7 Hz, CH_3_). ^13^C{^1^H} NMR (125 MHz, CDCl_3_) δ: 171.0 (C=O), 80.9 (C-3), 60.5 (CH_2_), 55.3, 49.9, 48.0, 47.9 (C), 44.5, 42.8 (C), 40.9 (C), 38.3 (CH_2_), 37.8 (C), 37.0 (C), 36.8, 34.2 (CH_2_), 34.0 (CH_2_), 29.4, 29.3 (CH_2_), 27.9, 26.9 (CH_2_), 26.8 (CH_2_), 23.7 (CH_2_), 22.9, 21.7 (CH_2_), 21.3, 20.8 (CH_2_), 18.2 (CH_2_), 16.5, 16.1, 15.9, 14.9, 14.6.

### 3β-*O*-Acetyl-19α-isopropyl-28,29,30-trinor-18α-olean-17(22)-ene (**2**)

Compound **2** was prepared from dihydrobetulin 3-*O*-acetate (**8**) according to the procedure published for betulin 3-*O*-acetate.^57^ POCl_3_ (19.5 mL, 210 mmol) was added to a solution of dihydrobetulin 3-acetate (**8**, 6.33 g, 13.00 mmol) in anhydrous pyridine (50 mL) and heated at 60 °C in an oil bath for 24 h. Then, the mixture was carefully poured onto ice (500 g). The product was extracted with chloroform (3 × 100 mL), and the organic extracts were concentrated under reduced pressure and filtered through a short silica path. Organic solvents were evaporated; column chromatography of the residue (hexane–ethyl acetate, 40:1 to 20:1) gave the title compound (**2**, 5.98 g, 98%) as a foam, sufficiently pure for further transformation. [α]_D_^20^ −20.5 (*c* 0.2, chloroform); lit.^23^ [α]_D_^20^ −31 (*c* 1.40). ν_max_ (film): 2946, 2869, 1732, 1451, 1370, 1247, 1026, 981, 740 cm^–1^. ^1^H NMR (500 MHz, CDCl_3_) δ: 5.32–5.33 (bs, 1 H, H-22), 4.49 (dd, 1 H, *J* 5.6 and 10.8 Hz, H-3), 2.04 (s, 3 H, CH_3_), 1.05 (s, 3 H, CH_3_), 0.95 (s, 3 H, CH_3_), 0.88 (d, 3 H, *J* 6.5 Hz, CH_3_), 0.87 (d, 3 H, *J* 6.6 Hz, CH_3_), 0.86 (s, 3 H, CH_3_), 0.85 (s, 3 H, CH_3_), 0.84 (s, 3 H, CH_3_), 1.96–2.14 (m, 23 H), 0.80–1.07 (m, 3 H). ^13^C{^1^H} NMR (125 MHz, CDCl_3_) δ: 171.0, 141.6 (C-17), 117.5 (C-22), 80.9 (C-3), 55.4, 50.3, 43.3, 42.6 (C), 40.9 (C), 40.2, 38.5 (CH, CH_2_), 37.8 (C), 37.1 (C), 34.5 (CH_2_), 34.0 (CH_2_), 33.2 (CH_2_), 28.1, 27.9, 25.6 (CH_2_), 23.7 (CH_2_), 22.1 (CH_2_), 21.3, 21.2 (CH_2_), 21.1 (CH_2_), 20.8 (CH_2_), 20.7, 18.2 (CH_2_), 16.5, 16.3, 15.8, 14.8. Anal. Calcd for C_32_H_52_O_2_: C, 81.99; H, 11.18. Found: C, 82.09; H, 11.20.

### 3-*O*-Acetyl-19α-isopropyl-28,29,30-trinor-17α,18α-oleanan-3β,17α,22α-triol (**9**)^58^

To a solution of **2** (1.000 g, 2.133 mmol) in pyridine (30 mL) was added OsO_4_ (600 mg, 2.36 mmol), and the mixture was stirred in the dark for 7 days. Then, pyridine was co-evaporated with toluene under reduced pressure, and the residue was dissolved in ethyl acetate. Water (40 mL), Na_2_S_2_O_5_ (2.0 g), and Na_2_S_2_O_3_·5H_2_O (3.0 g) were added, and the mixture was stirred until decomposition of osmate ester was detected on TLC (2–3 days). Then, water (100 mL) was added, and the product was extracted with chloroform (3 × 30 mL). Combined organic extracts were concentrated and the residue was purified by column chromatography (hexane–ethyl acetate, 20:1 to 7:3) to afford 860 mg (80%) of the title compound as a foam. [α]_D_^20^ +36.2 (*c* 0.2, chloroform). ν_max_ (film): 3479, 2946, 2868, 1730, 1716, 1451, 1374, 1248, 1032, 979, 736 cm^–1^. ^1^H NMR (600 MHz, CDCl_3_) δ: 4.48 (dd, 1 H, *J* 5.3 and 11.1 Hz, H-3), 3.79 (dd, 1 H, *J* 5.3 and 10.8 Hz, H-22), 2.29–2.35 (m, 1 H, H-19a), 2.05 (s, 3 H, COCH_3_), 2.00–2.03 (m, 1 H, H-16), 1.88–1.90 (m, 1 H, H-18), 1.74 (H-12), 1.69 (H-1), 1.69 (H-20), 1.68 (H-21), 1.67 (H-13), 1.65 (H-2), 1.61 (H-2), 1.61 (H-21), 1.57 (H-15), 1.52 (H-6), 1.52 (H-11), 1.44 (H-20), 1.40 (H-7), 1.40 (H-16), 1.39 (H-6), 1.36 (H-9), 1.24 (H-11), 1.22 (H-19), 1.16 (H-15), 1.02 (H-1), 1.01 (s, 3 H, H-27), 0.98 (s, 3 H, H-26), 0.91 (d, *J* 6.4 Hz, H-19b), 0.88 (H-12), 0.87 (s, 3 H, H-25), 0.85 (d, *J* 6.6 Hz, H-19c), 0.85 (s, 3 H, H-23), 0.84 (s, 3 H, H-24), 0.80–0.82 (m, 1 H, H-5). ^13^C{^1^H} NMR (150 MHz, CDCl_3_) δ: 171.0 (C=O), 80.9 (C-3), 74.9 (C-17), 68.0 (C-22), 55.4 (C-5), 50.1 (C-9), 43.2 (C-18), 41.3 (C-14), 40.9 (C-8), 40.3 (C-19), 38.4 (C-1), 37.8 (C-4), 37.0 (C-10), 36.5 (C-13), 34.0 (C-7), 31.8 (C-16), 28.1 (C-19a), 28.0 (C-15), 27.9 (C-23), 26.6 (C-21), 25.8 (C-12), 23.7 (C-2), 22.4 (C-19c), 22.3 (C-19b), 21.4 (C-20), 21.3 (C-11). 18.2 (C-6), 16.5 (C-24), 16.3 (C-25), 15.6 (C-26), 15.0 (C-27). HRMS (ESI-TOF) *m*/*z*: [M + Na]^+^ calcd for C_32_H_54_O_4_Na 525.3920; found 525.3908.

### Compounds **10** and **11**

#### Method C

To a vigorously stirred suspension of silica gel (230–400 mesh, 400 mg) in CH_2_Cl_2_ (4 mL) was added a solution of NaIO_4_ (56 mg, 0.26 mmol) in water (1 mL), and the heterogeneous mixture was stirred for 15 min to form a flaky suspension. Diol **9** (100 mg, 0.200 mmol) in CH_2_Cl_2_ (5 mL) was then added and stirred for 7 days. The mixture was filtered through sintered glass, silica gel was washed with CH_2_Cl_2_, and the solvents were evaporated under reduced pressure. The residue was purified by column chromatography (hexane–ethyl acetate, 9:1 to 5:1) to afford **11** (27 mg, 27%) as a foam and recovered diol **9** (53 mg, 53%).

#### Method D

A mixture of diol **9** (201 mg, 0.400 mmol) and lead tetraacetate (320 mg, 0.65 mmol) in pyridine (10 mL) was stirred at room temperature for 30 min. Then, two drops of glycerin were added to decompose an excess of lead tetraacetate and the solvents were co-evaporated with toluene under diminished pressure. The residue was purified by column chromatography (hexane–ethyl acetate, 9:1 to 5:1) to afford **10** (156 mg, 78%) and **11** (14 mg, 7%), both as a foams.

#### Method E

A mixture of diol **9** (402 mg, 0.800 mmol) and lead tetraacetate (600 mg, 1.20 mmol) in benzene (15 mL) was stirred at room temperature for 30 min. Then, two drops of glycerin were added to decompose an excess of lead tetraacetate and the solvents were evaporated under diminished pressure. The residue was purified by column chromatography (hexane–ethyl acetate, 9:1 to 5:1) to afford **10** (360 mg, 90%) as a foam.

#### Data for **10**

[α]_D_^20^ +29.0 (*c* 0.2, chloroform). ν_max_ (film): 2948, 2872, 1728, 1451, 1369, 1247, 1026, 981, 737 cm^–1^. ^1^H NMR (600 MHz, CDCl_3_) δ: 9.74–9.75 (m, 1 H, H-22), 4.49 (dd, 1 H, *J* 5.3 and 11.2 Hz, H-3), 2.48–2.54 (m, 1 H, H-21), 2.35–2.42 (m, 2 H, H-16, H-21) 2.26–2.29 (m, 1 H, H-18), 2.23–2.26 (m, 1 H, H-16), 2.05 (C*H*_3_C=O), 2.00 (H-13), 2.00 (H-19a), 1.84 (H-15), 1.83 (H-20), 1.72 (H-1), 1.70 (H-12), 1.68 (H-20), 1.64 (H-2), 1.58 (H-15), 1.56 (H-6), 1.55 (H-11), 1.46 (H-7), 1.42 (H-6), 1.40 (H-9), 1.31 (H-11), 1.22 (H-19), 1.12 (H-12), 1.09 (s, 3 H, H-27), 1.05 (H-1), 1.02 (s, 3 H, H-26), 0.93 (d, 1 H, *J* 6.7 Hz, H-19c), 0.90 (s, 3 H, H-25), 0.87 (s, 3 H, H-23), 0.86 (s, 3 H, H-24), 0.84 (H-5), 0.82 (d, 1 H, *J* 6.7 Hz, H-19b). ^13^C{^1^H} NMR (150 MHz, CDCl_3_) δ: 213.7 (C-17), 202.4 (C-22), 171.0 (CH_3_*C*=O), 80.8 (C-3), 55.6 (C-5), 52.6 (C-18), 50.7 (C-9), 44.6 (C-21), 44.2 (C-19), 41.4 (C-13), 41.2 (C-8), 40.8 (C-14), 38.8 (C-16), 38.6 (C-1), 37.8 (C-4), 37.1 (C-10), 34.1 (C-7), 30.9 (C-15), 30.7 (C-19a), 27.9 (C-23), 27.5 (C-12), 23.7 (C-2), 22.2 (C-20), 21.9 (C-19c), 21.8 (C-19b), 21.3 (*C*H_3_C=O), 21.1 (C-11), 18.1 (C-6), 16.5 (C-24, C-25), 15.7 (C-26), 14.7 (C-27). HRMS (ESI-TOF) *m*/*z*: [M + Na]^+^ calcd for C_32_H_52_O_4_Na 523.3763; found 523.3761.

#### Data for **11**

[α]_D_^20^ +30.6 (*c* 0.2, chloroform). ν_max_ (film): 3486, 2948, 2870, 1712, 1450, 1374, 1248, 1024, 981, 756 cm^–1^. ^1^H NMR (500 MHz, CDCl_3_) δ: 9.81 (d, 1 H, *J* 2.7 Hz, H-22), 4.48 (dd, 1 H, *J* 5.3 and 11.1 Hz, H-3), 2.80 (ddd, 1 H, *J* 2.8, 8.1, and 11.3 Hz, H-21), 2.04 (s, 3 H, C*H*_3_C=O), 2.01 (H-20), 1.96 (H-20), 1.94 (H-16), 1.70 (H-1), 1.69 (H-12), 1.69 (H-16), 1.66 (H-19a), 1.65 (H-2), 1.61 (H-2), 1.54 (H-11), 1.52 (H-6), 1.50 (H-19), 1.49 (H-15), 1.44 (H-18), 1.40 (H-6), 1.40 (H-7), 1.36 (H-9), 1.27 (H-15), 1.19 (H-11), 1.08 (H-13), 1.02 (H-1), 0.95 (H-12), 0.95 (s, 3 H, H-27), 0.94 (s, 3 H, H-26), 0.91 (d, 3 H, *J* 6.5 Hz, H-19c), 0.90 (d, 3 H, *J* 6.7 Hz, H-19b), 0.87 (s, 3 H, H-25), 0.85 (s, 3 H, H-23), 0.84 (s, 3 H, H-24), 0.81 (H-5). ^13^C{^1^H} NMR (125 MHz, CDCl_3_) δ: 206.5 (C-22), 171.0 (CH_3_*C*=O), 83.1 (C-17), 80.8 (C-3), 55.5 (C-5), 55.5 (C-21), 53.6 (C-18), 51.1 (C-9), 50.2 (C-19), 42.7 (C-13), 40.8 (C-14), 40.5 (C-8), 38.6 (C-1), 37.8 (C-4), 37.1 (C-10), 34.8 (C-19a), 33.9 (C-7), 31.3 (C-16), 29.0 (C-20), 28.1 (C-15), 27.9 (C-23), 26.7 (C-12), 23.6 (C-2), 22.7 (C-19b), 21.4 (C-11), 21.3 (*C*H_3_C=O), 20.7 (C-19c), 18.1 (C-6), 16.6 (C-25), 16.5 (C-24), 15.5 (C-26), 14.6 (C-27). HRMS (ESI-TOF) *m*/*z*: [M + Na]^+^ calcd for C_32_H_52_O_4_Na 523.3763; found 523.3757.

### Ozonolysis of **2**

#### Method F

Ozone was bubbled through a solution of **2** (4.69 g, 10.00 mmol) in MeOH (100 mL) and CH_2_Cl_2_ (100 mL) at −78 °C until the disappearance of the starting material on TLC (30 min). Oxygen was passed through the solution for an additional 15 min to remove an excess of ozone, and Me_2_S_2_ (10 mL) was added. The mixture was then left to warm to room temperature and stirred overnight. Solvents were evaporated under reduced pressure and the residue was purified by column chromatography (hexane–ethyl acetate, 9:1 to 5:1) to afford **13** (320 mg, 6%),^58^**12** (3.06 g, 56%), **11** (902 mg, 18%), and crude **14** (565 mg, 11%) in order of appearance, all as foams.

#### Method G

Ozone was bubbled through a solution of **2** (910 mg, 1.94 mmol) and *p*-TsOH (100 mg) in MeOH (25 mL) and CH_2_Cl_2_ (25 mL) at −78 °C until the disappearance of the starting material on TLC (30 min). Oxygen was passed through the solution for an additional 15 min to remove an excess of ozone. Then, the mixture was stirred for 1 h at room temperature to ensure the complete acetal formation and Me_2_S_2_ (1 mL) was added. The reaction was worked up following *Method F*. Acetal **12** (754 mg, 71%) was obtained as the sole product.

#### Data for **12**

[α]_D_^20^ +35.2 (*c*, 0.3 chloroform). ν_max_ (film): 2949, 2873, 1732, 1708, 1452, 1370, 1246, 1126, 1026, 980, 736 cm^–1^. ^1^H NMR (600 MHz, CDCl_3_) δ: 4.49 (dd, 1 H, *J* 5.1 and 11.3 Hz, H-3), 4.28–4.30 (m, 1 H, H-22), 3.31 (s, 3 H, OCH_3_), 3.30 (s, 3 H, OCH_3_), 2.33–2.39 (m, 1 H), 2.22–2.27 (m, 2 H), 2.05 (s, 3 H, COCH_3_), 1.93–1.98 (m, 2 H), 1.84 (ddd, 1 H, *J* 6.1, 6.1, and 13.0 Hz), 0.96–1.77 (m), 1.06 (s, 3 H, CH_3_), 1.01(s, 3 H, CH_3_), 0.93 (d, 3 H, *J* 6.7 Hz, CH_3_), 0.89 (s, 3 H, CH_3_), 0.86 (s, 3 H, CH_3_), 0.85 (s, 3 H, CH_3_), 0.82 (d, 3 H, *J* 6.7 Hz, CH_3_). ^13^C{^1^H} NMR (150 MHz, CDCl_3_) δ: 214.0 (C-17), 171.0, 104.7 (C-22), 80.7 (C-3), 55.5, 52.9, 52.8, 52.5, 50.7, 45.1, 41.4, 41.1 (C), 40.6 (C), 38.7 (CH_2_), 38.5 (CH_2_), 37.8 (C), 37.1 (C), 34.1 (CH_2_), 33.9 (CH_2_), 31.2, 30.6 (CH_2_), 27.9, 27.6 (CH_2_), 25.5 (CH_2_), 23.6 (CH_2_), 21.9 (CH_3_, CH_3_), 21.3, 21.1 (CH_2_), 18.1 (CH_2_), 16.5 (CH_3_, CH_3_), 15.6, 14.8. HRMS (ESI-TOF) *m*/*z*: [M + Na]^+^ calcd for C_34_H_58_O_5_Na 569.4182; found 569.4167.

#### Data for **13**

[α]_D_^20^ +32.5 (*c*, 0.2 chloroform). ν_max_ (film): 2949, 2872, 1736, 1706, 1451, 1368, 1245, 1170, 1026, 756 cm^–1^. ^1^H NMR (600 MHz, CDCl_3_) δ: 4.49 (dd, 1 H, *J* 5.2 and 11.3 Hz, H-3), 3.66 (s, 3 H, COC*H*_3_), 2.36 (H-16), 2.35 (H-21), 2.26 (H-21), 2.25 (H-16), 2.25 (H-18), 2.05 (s, 3 H, C*H*_3_CO), 1.98 (H-13), 1.97 (H-19a), 1.85 (H-15), 1.85 (H-20), 1.72 (H-1), 1.71 (H-12), 1.66 (H-20), 1.62 (H-2), 1.60 (H-2), 1.56 (H-6), 1.56 (H-15), 1.55 (H-11), 1.48 (H-7), 1.43 (H-7), 1.42 (H-6), 1.39 (H-9), 1.30 (H-11), 1.21 (H-19), 1.11 (H-12), 1.07 (s, 3 H, H-27), 1.05 (H-1), 1.02 (s, 3 H, H-26), 0.93 (d, 3 H, *J* 6.7 Hz, H-19b), 0.90 (s, 3 H, H-26), 0.87 (s, 3 H, H-23), 0.85 (s, 3 H, H-24), 0.84 (H-5), 0.82 (d, 3 H, *J* 6.7 Hz, H-19c). ^13^C{^1^H} NMR (150 MHz, CDCl_3_) δ: 213.7 (C-17), 174.1 (C-22), 171.0 (CH_3_*C*=O), 80.7 (C-3), 55.5 (C-5), 52.7 (C-18), 51.5 (CO_2_*C*H_3_), 50.7 (C-9), 44.2 (C-19), 41.3 (C-13), 41.1 (C-8), 40.7 (C-14), 38.7 (C-16), 38.5 (C-1), 37.8 (C-4), 37.1 (C-10), 34.8 (C-21), 34.1 (C-7), 30.8 (C-19a), 30.7 (C-15), 27.9 (C-23), 27.5 (C-12), 25.7 (C-20), 23.6 (C-2), 21.8 (C-19c), 21.8 (C-19b), 21.3 (*C*H_3_CO), 21.1 (C-11), 18.1 (C-6), 16.5 (C-25), 16.5 (C-24), 15.6 (C-26), 14.7 (C-27). HRMS (ESI-TOF) *m*/*z*: [M + Na]^+^ calcd for C_33_H_54_O_5_Na 553.3869; found 553.3863.

#### Selected Data for **14**

^13^C{^1^H} NMR (125 MHz, CDCl_3_) δ: 213.9 (C-17), 179.5 (C-22), 171.0 (CH_3_*C*=O), 80.8 (C-3), 55.5, 52.7, 50.8, 44.3, 41.2, 41.1 (C), 40.7 (C), 38.7 (CH_2_), 38.6 (CH_2_), 37.8 (C), 37.1 (C), 34.8 (CH_2_), 34.1 (CH_2_), 30.8, 30.7 (CH_2_), 27.9, 27.5 (CH_2_), 25.5 (CH_2_), 23.6 (CH_2_), 21.9, 21.8, 21.3, 21.0 (CH_2_), 18.1 (CH_2_), 16.5 (2 × CH_3_), 15.6, 14.7.

### Compound **14**

#### Method H

Aldehyde **10** (201 mg, 0.400 mmol) was dissolved in a mixture of THF (5 mL), *tert*-BuOH (15 mL), and 2-methyl-2-butene (5 mL). The solution was cooled in an ice bath, and a solution of NaH_2_PO_4_·2H_2_O (600 mg) and NaClO_2_ (720 mg) in water (10 mL) was added. The solution was stirred at 0 °C for 10 min; then the temperature was raised to room temperature and stirring was continued for 30 min. A saturated solution of NH_4_Cl (0.5 mL) and 15 mL of water were added. Product was extracted with dichloromethane (3 × 100 mL), and the combined organic extracts were evaporated to dryness. Short column chromatography of the residue (hexane–ethyl acetate, 9:1 to 5:1, and hexane–ethyl acetate–methanol, 5:3:1) gave crude acid **14** (202 mg) as an amorphous powder.^58^

#### Method I

To a cooled in an ice bath solution of **10** (100 mg, 0.200 mmol) in acetone (10 mL) was added Jones reagent (0.8 mL) dropwise, and the mixture was stirred at room temperature for 1 h. Then, isopropanol (1 mL) was added and stirring was continued for an additional 20 min. The solution was decanted, and the precipitated solid mass was washed with acetone (4 × 10 mL). Combined organic extracts were evaporated under reduced pressure and the residue was purified by column chromatography (hexane–ethyl acetate, 5:1, to hexane–ethyl acetate–methanol, 5:3:1) to afford crude acid **14** (100 mg) as amorphous powder.

### Compound **17**

Crude acid **14** (100 mg, 0.194 mmol) was dissolved in methanol (6 mL), and acetyl chloride (50 μL) was added. The mixture was stirred at room temperature for 24 h. The reaction was quenched with Et_3_N (0.3 mL), and the solvents were evaporated under reduced pressure. Column chromatography of the residue (hexane–ethyl acetate, 7:3 to 1:1) gave **17** (76 mg, 75% after two steps) as a glass.^58^ [α]_D_^20^ +17.4 (*c*, 0.2 chloroform). ν_max_ (film): 3461, 2947, 2870, 1736, 1704, 1449, 1384, 1254, 1172, 1038, 755 cm^–1^. ^1^H NMR (500 MHz, CDCl_3_) δ: 3.66 (s, 3 H, OCH_3_), 3.21 (dd, 1 H, *J* 5.0 Hz, 11.5 Hz, H-3), 2.32–2.40 (m, 2 H), 2.22–2.29 (m, 3 H), 1.95–2.01 (m, 2 H), 1.82–1.88 (m, 2 H), 1.19–1.74 (m), 1.07 (s, 3 H, CH_3_), 1.02 (s, 3 H, CH_3_), 0.99 (s, 3 H, CH_3_), 0.93 (d, 3 H, *J* 6.7 Hz, H-19b), 0.87 (s, 3 H, CH_3_), 0.82 (d, 3 H, *J* 6.7 Hz, H-19c), 0.78 (s, 3 H, CH_3_), 0.72–1.35 (m). ^13^C{^1^H} NMR (125 MHz, CDCl_3_) δ: 213.8 (C-17), 174.1 (C-22), 78.8 (C-3), 55.4, 52.7, 51.5, 50.8, 44.3, 41.3, 41.1 (C), 40.7 (C), 38.9 (C, CH_2_), 38.7 (CH_2_), 37.2 (C), 34.8 (CH_2_), 34.2 (CH_2_), 30.8, 30.7 (CH_2_), 27.9, 27.6 (CH_2_), 27.4 (CH_2_), 25.7 (CH_2_), 21.8, 21.8, 21.1 (CH_2_), 18.2 (CH_2_), 16.4, 15.6, 15.4, 14.8. HRMS (ESI-TOF) *m*/*z*: [M + Na]^+^ calcd for C_31_H_52_O_4_Na 511.3763; found 511.3767.

### Compound **13**

#### Method J

Crude acid **14** (100 mg, 0.194 mmol) was dissolved in DMF (5 mL), to which K_2_CO_3_ (150 mg), and MeI (100 μL) were added. The mixture was stirred at room temperature for 24 h, and the solvents were evaporated under reduced pressure. Column chromatography of the residue (hexane–ethyl acetate, 7:3 to 1:1) gave **13** (80 mg, 75% after two steps) as a glass.

#### Method K

Acetylation of ester **17** (35 mg, 0.072 mmol) with acetyl anhydride (1.0 mL) in pyridine (2 mL) at room temperature for 24 h, followed by the usual workup and column chromatography (hexane–ethyl acetate, 9:1 to 7:3), gave **13** (36 mg, 95%) as a glass.

### Compound **19**

Aldehyde **11** (495 mg, 0.988 mmol) was dissolved in THF (5 mL), *tert*-BuOH (25 mL), and 2-methyl-2-butene (8 mL). The solution was cooled in an ice bath, and a solution of NaH_2_PO_4_·2H_2_O (1.3 g) and NaClO_2_ (1.0 g) in water (12 mL) was added. The solution was stirred at 0 °C for 30 min; then the temperature was raised to room temperature and the mixture was stirred for 2 h. The mixture was poured into 10 mL of a saturated solution of NH_4_Cl and 50 mL of water. The product was extracted with dichloromethane (4 × 30 mL), and the combined organic extracts were evaporated to dryness. Column chromatography of the residue (hexane–ethyl acetate, 9:1 to 5:1, and finally hexane–ethyl acetate–methanol, 5:3:1) gave crude acid **18** (449 mg, 88%) as an amorphous powder. HRMS (ESI-TOF) *m*/*z*: [M + Na]^+^ calcd for C_32_H_52_O_5_ 539.3712; found 539.3693.

Crude **18** (422 mg, 0.817 mmol) was then dissolved in DMF (5 mL), K_2_CO_3_ (300 mg), and MeI (500 μL) was added. The mixture was stirred at room temperature for 2 h. Then, the solvents were evaporated under reduced pressure. Column chromatography of the residue (hexane–ethyl acetate, 9:1 to 5:1) gave **19** (346 mg, 80%) as a foam. [α]_D_^20^ +60.3 (*c*, 0.4 chloroform). ν_max_ (film): 3508, 2949, 2871, 1728, 1449, 1368, 1247, 1198, 1175, 1023, 982, 756 cm^–1^. ^1^H NMR (500 MHz, CDCl_3_) δ: 4.48 (dd, 1 H, *J* 5.1 and 11.4 Hz, H-3), 3.71 (s, 3 H, OCH_3_), 2.89 (dd, 1 H, *J* 7.7 and 12.5 Hz, H-21), 2.05 (H-20), 2.04 (s, 3 H, CH_3_CO), 1.83 (H-20), 1.75 (H-16), 1.70 (H-1), 1.68 (H-12), 1.65 (H-2), 1.61 (H-2), 1.53 (H-11), 1.52 (H-6), 1.49 (H-19), 1.42 (H-19a), 1.40 (H-15), 1.38 (H-6), 1.37 (H-7), 1.37 (H-9), 1.22 (H-15), 1.19 (H-11), 1.07 (H-13), 1.02 (H-1), 0.97 (H-12), 0.96 (s, 3 H, H-27), 0.94 (s, 3 H, H-26), 0.88 (d, 3 H, *J* 6.9 Hz, H-19c), 0.87 (s, 3 H, H-25), 0.86 (d, 3 H, *J* 6.7 Hz, H-19b), 0.85 (s, 3 H, H-23), 0.84 (s, 3 H, H-24), 0.81 (H-5). ^13^C{^1^H} NMR (125 MHz, CDCl_3_) δ: 176.1 (C-22), 171.0 (CH_3_*C*=O), 80.8 (C-17), 80.8 (C-3), 55.5 (C-5), 51.7 (CO_2_*CH*_3_), 51.0 (C-9), 51.0 (C-19), 50.2 (C-19a), 47.8 (C-21), 42.4 (C-13), 40.8 (C-8 or C-14), 40.4 (C-8 or C-14), 38.5 (C-1), 37.7 (C-4), 37.1 (C-10), 34.5 (C-18), 33.8 (C-7), 32.3 (C-20), 30.8 (C-16), 28.1 (C-15), 27.9 (C-23), 26.7 (C-12), 23.6 (C-2), 22.8 (C-19c), 21.4 (C-11), 21.3 (*C*H_3_C=O), 20.7 (C-19b), 18.1 (C-7), 16.6 (C-25), 16.4 (C-24), 15.5 (C-26), 14.5 (C-27). HRMS (ESI-TOF) *m*/*z*: [M + Na]^+^ calcd for C_33_H_54_O_5_Na 553.3869; found 553.3873.

### Compound **20**

Ester **19** (103 mg, 0.194 mmol) was dissolved in methanol (5 mL), and acetyl chloride (50 μL) was added. The mixture was stirred at room temperature for 24 h. The reaction was quenched with Et_3_N (0.3 mL), and the solvents were evaporated under reduced pressure. Column chromatography of the residue (hexane–ethyl acetate, 5:1 to 7:3) gave **20** (94 mg, 98%) as a foam. [α]_D_^20^ +56.1 (*c*, 0.3 chloroform). ν_max_ (film): ν_max_ (film): 3489, 2946, 2870, 1713, 1450, 1374, 1212, 1197, 1175, 1031, 756 cm^–1^. ^1^H NMR (600 MHz, CDCl_3_) δ: 3.72 (s, 3 H, OCH_3_), 3.20 (dd, 1 H, *J* 4.8, 11.5 Hz, H-3), 2.89 (dd, 1 H, *J* 7.7, 12.5 Hz, H-3), 2.03–2.08 (m, 1 H), 1.80–1.86 (m, 1 H), 1.48–1.76 (m, 11 H), 1.34–1.45 (m, 6 H), 1.14–1.24 (m, 2 H), 1.05–1.09 (m, 1 H), 0.97 (s, 3 H, CH_3_), 0.96 (s, 3 H, CH_3_), 0.94 (s, 3 H, CH_3_), 0.88 (d, 3 H, *J* 6.7 Hz, H-19b), 0.86 (d, 3 H, *J* 6.5 Hz, H-19c), 0.84 (s, 3 H, CH_3_), 0.76 (s, 3 H, CH_3_), 0.69–0.71 (m, 1 H, H-5). ^13^C{^1^H} NMR (150 MHz, CDCl_3_) δ: 176.1 (C=O), 80.9 (C-17), 78.9 (C-3), 55.4, 51.7, 51.2, 51.1, 50.2, 47.8, 42.5, 40.9 (C), 40.4 (C), 38.9 (CH_2_), 38.8 (C), 37.2 (C), 34.6, 34.0 (CH_2_), 32.2 (CH_2_), 30.9 (CH_2_), 28.1 (CH_2_), 27.9, 27.4 (CH_2_), 26.8 (CH_2_), 22.8, 21.4 (CH_2_), 20.7, 18.2 (CH_2_), 16.5, 15.6, 15.3, 14.5. HRMS (ESI-TOF) *m*/*z*: [M + Na]^+^ calcd for C_31_H_52_O_4_Na 511.3763; found 511.3764.

### Compound **21**

A solution of aldehyde **11** (100 mg, 0.200 mmol) in THF (8 mL) was added to a suspension of LiAlH_4_ (100 mg, 2.6 mmol) in THF (2 mL), and the mixture was stirred at room temperature under an argon atmosphere for 2.5 h. Then, sat. NH_4_Cl (few drops) and ethyl acetate (5 mL) were added. The whole mixture was filtered through a short silica pad, eluted with hexane–ethyl acetate (1:1), and evaporated to dryness. To the residue were added pyridine (3 mL) and acetic anhydride (2 mL), and the mixture was stirred at room temperature for 24 h. Solvents were co-evaporated with toluene under reduced pressure. Column chromatography (hexane–ethyl acetate, 5:1 to 7:3) of the residue afforded diacetate **21** (86 mg, 79% after two steps) as a white solid. mp 224–226 °C; [α]_D_^20^ +32.2 (*c*, 0.25 chloroform). ^1^H NMR (600 MHz, CDCl_3_) δ: 4.48 (dd, 1 H, *J* 5.2 and 11.2 Hz, H-3), 4.28 (dd, 1 H, *J* 6.0 and 11.2 Hz, H-22), 4.07 (dd, 1 H, *J* 6.8 and 11.2 Hz, H-22), 2.22–2.28 (m, 1 H, H-21), 2.05 (s, 3 H, CH_3_CO), 2.04 (s, 3 H, CH_3_CO), 1.91–1.96 (m, 1 H, H-20), 1.88 (m, 1 H, H-16), 1.71 (H-1), 1.68 (H-12), 1.65 (H-2), 1.61 (H-2), 1.60 (H-16), 1.54 (H-19a), 1.53 (H-11), 1.52 (H-6), 1.44 (H-15), 1.43 (H-7), 1.40 (H-18), 1.39 (H-6), 1.37 (H-7), 1.37 (H-9), 1.37 (H-19), 1.35 (H-20), 1.21 (H-15), 1.18 (H-11), 1.12 (H-13), 1.03 (H-1), 0.96 (s, 3 H, H-27), 0.94 (H-12), 0.94 (s, 3 H, H-26), 0.88 (H-19c), 0.87 (H-19b), 0.87 (s, 3 H, H-25), 0.85 (s, 3 H, H-23), 0.84 (s, 3 H, H-24), 0.81 (H-5). ^13^C{^1^H} NMR (150 MHz, CDCl_3_) δ: 171.1 (CH_3_*C*=O), 171.0 (CH_3_*C*=O), 80.9 (C-3), 80.7 (C-17), 64.6 (C-22), 55.6 (C-5), 53.6 (C-18), 51.1 (C-9), 49.0 (C-19), 42.9 (C-13), 41.9 (C-21), 41.0 (C-14), 40.5 (C-8), 38.6 (C-1), 37.8 (C-4), 37.1 (C-10), 35.0 (C-19a), 33.9 (C-7), 31.8 (C-20), 30.5 (C-16), 28.0 (C-15), 27.9 (C-23), 26.7 (C-12), 23.7 (C-2), 22.6 (C-19c), 21.5 (C-11), 21.3 (*C*H_3_C=O), 21.1 (*C*H_3_C=O), 20.6 (C-19b), 18.1 (C-6), 16.6 (C-25), 16.5 (C-24), 15.6 (C-26), 14.8 (C-27). HRMS (ESI-TOF) *m*/*z*: [M + Na]^+^ calcd for C_34_H_56_O_5_Na 567.4025; found 567.4006.

### Cyclization of **10** and **12** by Intramolecular Aldol Reaction

#### Method L

A solution of **12** (273 mg, 0.500 mmol) and *p*-TsOH (150 mg) in acetone (10 mL) was stirred at room temperature for 24 h. The whole mixture was then filtered through a short silica gel pad and concentrated under reduced pressure, and the residue was purified by column chromatography (hexane–ethyl acetate, 9:1 to 7:3) to give **11** (45 mg, 18%) and a mixture of epimers **22** (148 mg, 59%), both as foams.

#### Method M

Starting from **10** (250 mg, 0.500 mmol), and following the *Method L*, **11** (50 mg, 20%) and **22** (140 mg, 56%) were obtained, both as foams.

#### Method N

Starting from **12** (273 mg, 0.500 mmol) and *p*-TsOH (150 mg) in toluene (10 mL), and following the *Method L*, **22** (125 mg, 50%) was obtained as foam.

#### Method O

Starting from **10** (250 mg, 0.500 mmol) and *p*-TsOH (150 mg) in toluene (10 mL), and following the *Method L*, **22** (185 mg, 74%) was obtained as foam.

### Compounds **23** and **24**

The acetylation of **22** (751 mg, 1.500 mmol) with acetyl chloride (2.0 mL) in pyridine (4 mL) at room temperature for 24 h, followed by the usual workup and column chromatography (hexane–ethyl acetate, 20:1 to 5:1), gave **23** (374 mg, 46%) and **24** (228 mg, 28%), both as a white solid. Crystals suitable for X-ray structure analysis were obtained by slow evaporation of a chloroform/methanol solution at room temperature.

#### Data for **23**

mp 214–216 °C; [α]_D_^20^ +45.3 (*c* 0.4, chloroform); ν_max_ (film): 2948, 2871, 1736, 1699, 1466, 1448, 1369, 1246, 1208, 1027, 982, 757 cm^–1^. ^1^H NMR (600 MHz, CDCl_3_) δ: 4.81 (ddd, 1 H, *J* 3.7, 5.9, and 11.5 Hz, H-22), 4.48 (dd, 1 H, *J* 5.0 and 11.5 Hz, H-3), 3.01–3.05 (m, 1 H, H-16), 2.15 (dd, 1 H, *J* 7.0 and 14.3 Hz, H-15), 2.07 (H-13), 2.05 (s, 3 H, COCH_3_), 2.03 (s, 3 H, COCH_3_), 2.00 (H-21), 1.94 (H-18), 1.82 (H-21), 1.78 (H-20), 1.73 (H-1), 1.73 (H-12), 1.66 (H-2), 1.62 (H-2), 1.58 (H-19a), 1.54 (H-6), 1.51 (H-11), 1.48 (H-15), 1.47 (H-7), 1.44 (H-7), 1.41 (H-19), 1.40 (H-6), 1.31 (H-9), 1.29 (H-11), 1.12 (s, 3 H, H-26), 1.10 (H-20), 1.09 (H-12), 1.04 (H-1), 0.91 (s, 3 H, H-25), 0.90 (d, 3 H, *J* 6.7 Hz, H-19b), 0.85 (d, 3 H, *J* 6.3 Hz, H-19c), 0.86 (s, 3 H, H-23), 0.85 (s, 3 H, H-24), 0.84 (s, 3 H, H-27), 0.81 (H-5). ^13^C{^1^H} NMR (150 MHz, CDCl_3_) δ: 215.7 (C-17), 170.9 (C=O), 169.9 (C=O), 80.7 (C-3), 75.4 (C-28), 55.4 (C-5), 54.4 (C-18), 50.7 (C-9), 49.9 (C-19), 49.0 (C-16), 47.5 (C-13), 41.0 (C-8), 39.7 (C-14), 38.5 (C-1), 37.7 (C-4), 37.1 (C-10), 34.1 (C-7), 33.8 (C-20), 29.9 (C-15), 29.8 (C-12), 27.9 (C-23), 27.7 (C-22), 25.5 (C-21), 23.6 (C-2), 21.3 (CH_3_), 21.2 (CH_3_), 21.2 (C-11), 20.7 (C-29), 19.7 (C-30), 18.0 (C-6), 16.6 (C-25), 16.5 (C-24), 16.0 (C-26), 15.7 (C-27). HRMS (ESI-TOF) *m*/*z*: [M + Na]^+^ calcd for C_34_H_54_O_5_Na 565.3869; found 565.3856.

#### Data for **24**

mp 279–282 °C; [α]_D_^20^ +44.2 (*c* 0.2, chloroform); ν_max_ (film): 2948, 2871, 1736, 1699, 1466, 1448, 1369, 1246, 1208, 1027, 982, 757 cm^–1^. ^1^H NMR (600 MHz, CDCl_3_) δ: 4.80 (ddd, 1 H, *J* 2.8, 2.8, and 5.7 Hz, H-22), 4.47 (dd, 1 H, *J* 5.0 and 11.5 Hz, H-3), 2.68–2.71 (m, 1 H, H-16), 2.08 (H-18), 2.06 (s, 3 H, COCH_3_), 2.05 (H-21), 2.04 (s, 3 H, COCH_3_), 2.00 (H-13), 1.88 (H-21), 1.86 (H-15), 1.73 (H-15), 1.72 (H-1), 1.72 (H-12), 1.69 (H-20), 1.65 (H-2), 1.65 (H-19a), 1.61 (H-2), 1.56 (H-20), 1.53 (H-6), 1.51 (H-11), 1.43 (H-7), 1.41 (H-7), 1.37 (H-6), 1.28 (H-9), 1.28 (H-11), 1.25 (H-19), 1.10 (H-12), 1.07 (H-1), 1.05 (s, 3 H, H-26), 0.95 (d, 3 H, *J* 6.7 Hz, H-19b), 0.90 (s, 3 H, H-25), 0.88 (d, 3 H, *J* 6.8 Hz, H-19c), 0.86 (s, 3 H, H-27), 0.85 (s, 3 H, H-23), 0.84 (s, 3 H, H-24), 0.80 (H-5). ^13^C{^1^H} NMR (150 MHz, CDCl_3_) δ: 215.0 (C-17), 170.9 (C=O), 170.5 (C=O), 80.7 (C-3), 73.5 (C-28), 55.4 (C-5), 55.4 (C-18), 50.7 (C-9), 50.2 (C-16), 47.6 (C-19), 46.1 (C-13), 40.8 (C-8), 39.7 (C-14), 38.5 (C-1), 37.8 (C-4), 37.1 (C-10), 35.0 (C-15), 34.2 (C-7), 32.2 (H-20), 29.6 (C-12), 27.9 (C-23), 26.3 (C-22), 23.6 (C-2), 23.2 (C-21), 21.4 (CH_3_), 21.3 (CH_3_), 21.2 (C-30), 21.1 (C-11), 20.2 (C-29), 18.0 (C-6), 16.5 (C-25), 16.5 (C-24), 16.0 (C-26), 15.7 (C-27). HRMS (ESI-TOF) *m*/*z*: [M + Na]^+^ calcd for C_34_H_54_O_5_Na 565.3869; found 565.3867.

## Supporting Information Available

The crystal structure determination of compounds **21**, **23**, and **24**; key HMBC and NOE correlations; copies of ^1^H and ^13^C{^1^H} NMR spectra for key compounds; the results of DFT calculations and atom coordinates for the key structures (PDF)

## Accession Codes

CCDC 2031169, 2031957, and 2055466 contain the supplementary crystallographic data for this paper. These data can be obtained free of charge via www.ccdc.cam.ac.uk/data_request/cif, or by emailing data_request@ccdc.cam.ac.uk, or by contacting The Cambridge Crystallographic Data Centre, 12 Union Road, Cambridge CB2 1EZ, UK; fax: +44 1223 336033.

## References

[ref1] Perthuison J.; Schaeffer P.; Debels P.; Galant P.; Adam P. Betulin-related esters from birch bark tar: Identification, origin and archaeological significance. Org. Geochem. 2020, 139, 103944 10.1016/j.orggeochem.2019.103944.

[ref2] Stacey R. J.; Dunne J.; Brunning S.; Deviese T.; Mortimer R.; Ladd S.; Parfitt K.; Evershed R.; Bull I. Birch bark tar in early Medieval England – Continuity of tradition or technological revival?. J. Archaeol. Sci. Rep. 2020, 29, 102118 10.1016/j.jasrep.2019.102118. 32190727 PMC7063695

[ref3] Hayek E. W. H.; Jordis U.; Moche W.; Sauter F. A bicentennial of betulin. Phytochemistry 1989, 28, 2229–2242. 10.1016/S0031-9422(00)97961-5.

[ref4] Krasutsky P. A. Birch bark research and development. Nat. Prod. Rep. 2006, 23, 919–942. 10.1039/b606816b. 17119640

[ref5] Alakurtti S.; Makela T.; Koskimies S.; Yli-Kauhaluoma J. Pharmacological properties of the ubiquitous natural product betulin. Eur. J. Pharm. Sci. 2006, 29, 1–13. 10.1016/j.ejps.2006.04.006. 16716572

[ref6] Jonnalagadda S.; Corsello M.; Sleet C. Betulin-betulinic acid natural product based analogs as anti-cancer agents. Anti-Cancer Agents Med. Chem. 2013, 13, 1477–1499. 10.2174/18715230113129990094. 23848199

[ref7] Hordyjewska A.; Ostapiuk A.; Horecka A.; Kurzepa J. Betulin and betulinic acid: triterpenoids derivatives with a powerful biological potential. Phytochem. Rev. 2019, 18, 929–951. 10.1007/s11101-019-09623-1.

[ref8] Amiri S.; Dastghaib S.; Ahmadi M.; Mehrbod P.; Khadem F.; Behrouj H.; Aghanoori M.-R.; Machaj F.; Ghamsari M.; Rosik J.; Hudecki A.; Afkhami A.; Hashemi M.; Los M. J.; Mokarram P.; Madrakian T.; Ghavami S. Betulin and its derivatives as novel compounds with different pharmacological effects. Biotechnol. Adv. 2020, 38, 107409 10.1016/j.biotechadv.2019.06.008. 31220568

[ref9] Cmoch P.; Korda A.; Rárová L.; Oklešt’ková J.; Strnad M.; Gwardiak K.; Karczewski R.; Pakulski Z. Synthesis of lupane-type saponins containing an unusual α-D-idopyranoside fragment as potent cytotoxic agents. Eur. J. Org. Chem. 2014, 2014, 4089–4098. 10.1002/ejoc.201402187.

[ref10] Wiemann J.; Heller L.; Perl V.; Kluge R.; Ströhl D.; Csuk R. Betulinic acid derived hydroxamates and betulin derived carbamates are interesting scaffolds for the synthesis of novel cytotoxic compounds. Eur. J. Med. Chem. 2015, 106, 194–210. 10.1016/j.ejmech.2015.10.043. 26547057

[ref11] Kuczynska K.; Cmoch P.; Rárová L.; Oklešt’ková J.; Korda A.; Pakulski Z.; Strnad M. Influence of intramolecular hydrogen bonds on regioselectivity of glycosylation. Synthesis of lupane-type saponins bearing the OSW-1 saponin disaccharide unit and its isomers. Carbohydr. Res. 2016, 423, 49–69. 10.1016/j.carres.2016.01.010. 26878488

[ref12] Borkova L.; Adamek R.; Kalina P.; Drašar P.; Dzubak P.; Gurska S.; Rehulka J.; Hajduch M.; Urban M.; Sarek J. Synthesis and cytotoxic activity of triterpenoid thiazoles derived from allobetulin, methyl betulonate, methyl oleanonate, and oleanonic acid. ChemMedChem 2017, 12, 390–398. 10.1002/cmdc.201600626. 28084676

[ref13] Kahnt M.; Heller L.; Grabandt P.; Al-Harrasi A.; Csuk R. Platanic acid: a new scaffold for the synthesis of cytotoxic agents. Eur. J. Med. Chem. 2018, 143, 259–265. 10.1016/j.ejmech.2017.11.046. 29197730

[ref14] Bildziukevich U.; Rárová L.; Šaman S.; Wimmer Z. Picolyl amides of betulinic acid as antitumor agents causing tumor cell apoptosis. Eur. J. Med. Chem. 2018, 145, 41–50. 10.1016/j.ejmech.2017.12.096. 29316537

[ref15] Mierina I.; Vilskersts R.; Turks M. Delivery systems for birch-bark triterpenoids and their derivatives in anticancer research. Curr. Med. Chem. 2020, 27, 1308–1336. 10.2174/0929867325666180530095657. 29848269

[ref16] Chrobak E.; Kadela-Tomanek M.; Bębenek E.; Marciniec K.; Wietrzyk J.; Trynda J.; Pawełczak B.; Kusz J.; Kasperczyk J.; Chodurek E.; Paduszyński P.; Boryczka S. New phosphate derivatives of betulin as anticancer agents: Synthesis, crystal structure, and molecular docking study. Bioorg. Chem. 2019, 87, 613–628. 10.1016/j.bioorg.2019.03.060. 30947097

[ref17] Juang Y.-P.; Liang P.-H. Biological and pharmacological effects of synthetic saponins. Molecules 2020, 25, 4974 10.3390/molecules25214974. PMC7663351 33121124

[ref18] Korda A.; Rárová L.; Pakulski Z.; Strnad M.; Oklešt’ková J.; Kuczynska K.; Cmoch P.; Gwardiak K.; Karczewski R. New lupane bidesmosides exhibiting strong cytotoxic activities *in vitro*. Bioorg. Chem. 2020, 100, 103868 10.1016/j.bioorg.2020.103868. 32388425

[ref19] Cmoch P.; Korda A.; Rárová L.; Oklešt’ková J.; Strnad M.; Luboradzki R.; Pakulski Z. Synthesis and structure–activity relationship study of cytotoxic lupane-type 3β-*O*-monodesmosidic saponins with an extended C-28 side chain. Tetrahedron 2014, 70, 2717–2730. 10.1016/j.tet.2014.03.006.

[ref20] Sidoryk K.; Korda A.; Rárová L.; Oklešt’ková J.; Strnad M.; Cmoch P.; Pakulski Z.; Gwardiak K.; Karczewski R.; Luboradzki R. Synthesis and biological activity of new homolupanes and homolupane saponins. Tetrahedron 2015, 71, 2004–2012. 10.1016/j.tet.2015.02.008.

[ref21] Sidoryk K.; Rárová L.; Oklešt’ková J.; Pakulski Z.; Strnad M.; Cmoch P.; Luboradzki R. Synthesis of 28a-homoselenolupanes and 28a-homoselenolupane saponins. Org. Biomol. Chem. 2016, 14, 10238–10248. 10.1039/C6OB01938B. 27735956

[ref22] Sidoryk K.; Korda A.; Rárová L.; Oklešt’ková J.; Pakulski Z.; Strnad M.; Cmoch P.; Gwardiak K.; Karczewski R. Synthesis and cytotoxicity of 28a-homothiolupanes and 28a-homothiolupane saponins, *Eur*. J. Org. Chem. 2016, 2016, 373–383. 10.1002/ejoc.201501147.

[ref23] Vystrčil A.; Křeček V.; Buděšínský M. Elimination reactions on angular hydroxymethyl group of the lupane skeleton. Collect. Czech. Chem. Commun. 1974, 39, 2494–2506. 10.1135/cccc19742494.

[ref24] Rice G. K.; Yokoi T.; Hayashi T.; Suzuki H.; Lee K.-H.; McPhail A. T. Structure and stereochemistry of radermasinin, a novel cytotoxic triterpene lactone from *Radermachia sinica*. X-Ray crystal structure of radermasinin monohydrate. J. Chem. Soc., Chem. Commun. 1986, 1397–1398. 10.1039/c39860001397.

[ref25] Thibeault D.; Gauthier C.; Legault J.; Bouchard J.; Dufour P.; Pichette A. Synthesis and structure–activity relationship study of cytotoxic germanicane- and lupane-type 3β-O-monodesmosidic saponins starting from betulin. Bioorg. Med. Chem. 2007, 15, 6144–6157. 10.1016/j.bmc.2007.06.033. 17614290

[ref26] Zaitsev A. B.; Adolfsson H. Recent developments in asymmetric dihydroxylations. Synthesis 2006, 2006, 1725–1756. 10.1055/s-2006-942378.

[ref27] Christie S. D. R.; Warrington A. D. Osmium and palladium: Complementary metals in alkene activation and oxidation. Synthesis 2008, 2008, 1325–1341. 10.1055/s-2008-1067031.

[ref28] Daumas M.; Vo-Quang Y.; Vo-Quang L.; Le Goffic F. A new and efficient heterogeneous system for the oxidative cleavage of 1,2-diols and the oxidation of hydroquinones. Synthesis 1989, 1989, 64–65. 10.1055/s-1989-27155.

[ref29] Hanson J. R. The ozonolysis of terpenoids, a Pandora’s box of by-products. J. Chem. Res. 2017, 41, 557–563. 10.3184/174751917X15064232103029.

[ref30] Audran G.; Marque S. R. A.; Santelli M. Ozone, chemical reactivity and biological functions. Tetrahedron 2018, 74, 6221–6261. 10.1016/j.tet.2018.09.023.

[ref31] Schreiber S. L.; Claus R. E.; Reagan J. Ozonolytic cleavage of cycloalkenes to terminally differentiated products. Tetrahedron Lett. 1982, 23, 3867–3870. 10.1016/S0040-4039(00)87729-1.

[ref32] Testero S. A.; Mangione M. I.; Poeylaut-Palena A. A.; Sierra M. G.; Spanevello R. A. Tetrahedron 2007, 63, 11410–11420. 10.1016/j.tet.2007.08.070.

[ref33] Van Ornum S. G.; Champeau R. M.; Pariza R. Ozonolysis applications in drug synthesis. Chem. Rev. 2006, 106, 2990–3001. 10.1021/cr040682z. 16836306

[ref34] Kuczkowski R. L. The structure and mechanism of formation of ozonides. Chem. Soc. Rev. 1992, 21, 79–83. 10.1039/cs9922100079.

[ref35] Hostettmann K.; Marston A. Saponins; Cambridge University Press: Cambridge, U.K., 1995.

[ref36] Clive D. L. J.; Wickens P. L.; da Silva G. V. J. Preparation of a semisynthetic analogue of mevinolin and compactin. J. Org. Chem. 1995, 60, 5532–5536. 10.1021/jo00122a037.

[ref37] Hsu D.-S.; Liao C.-C. First total syntheses of (±)-penicillones A and B. Org. Lett. 2007, 9, 4563–4565. 10.1021/ol702062p. 17902684

[ref38] Collins D. J.; Hobbs J. J.; Rawson R. J. A novel skeletal rearrangement during reduction of 6β-bromo-4β,5-epoxy-5β-cholestan-3β-ol with lithium aluminium hydride. Chem. Commun. 1967, 0, 135–136. 10.1039/C19670000135.

[ref39] Collins D. J.; Hobbs J. J.; Rawson R. J. 4,5-*Seco*-4,6-cyclosteroids. I. Structure and properties of the diol from reductive rearrangement of 6β-bromo-4β,5-epoxy-5β-cholestan-3β-ol. Aust. J. Chem. 1969, 22, 607–626. 10.1071/CH9690607.

[ref40] Collins D. J.; Hobbs J. J.; Rawson R. J. 4,5-*Seco*-4,6-cyclosteroids. II. Synthesis of 4,5-*seco*-4,6-cyclo-6β-cholestan-5α-ol. Aust. J. Chem. 1969, 22, 627–635. 10.1071/CH9690627.

[ref41] Collins D. J.; Hobbs J. J.; Rawson R. J. 4,5-*Seco*-4,6-cyclosteroids. III. Observations on the course of reductive rearrangement of 6β-bromo-4β,5-epoxy-5β-cholestan-3β-ol. Aust. J. Chem. 1969, 22, 807–819. 10.1071/CH9690807.

[ref42] Collins D. J.; Hobbs J. J.; Rawson R. J. 4,5-*Seco*-4,6-cyclosteroids. IV. Physical properties and the conformation of ring A. Aust. J. Chem. 1969, 22, 821–838. 10.1071/CH9690821.

[ref43] Coxon J. N.; Hartshorn M. P.; Rae W. J. Reactions of epoxides-XXVI. The BF_3_-catalysed rearrangement of 5,6α-epoxy-6β-phenyl-5α-cholestane and a novel ketone rearrangement. Tetrahedron 1970, 26, 1091–1094. 10.1016/S0040-4020(01)98785-X. 5443310

[ref44] ApSimon J. W.; Badripersaud S.; Hooper J. W.; Pike R.; Birnbaum G. I.; Huber C.; Post M. L. Approaches to the synthesis of triterpenoids. IV. The ABC + E ring approach to the pentacyclic triterpene skeleton. Synthesis of a pentacyclic compound suitable for triterpene synthesis. Can. J. Chem. 1978, 56, 2139–2149. 10.1139/v78-350.

[ref45] Li T.-S.; Li Y.-L.; Liang X.-T. Studies of the synthesis of biomarkers. VII. Synthesis of 5α-(17R,20R)-14,l5-secocholestane. Steroids 1990, 55, 263–265. 10.1016/0039-128X(90)90042-A. 2385850

[ref46] Deng J.; Ning Y.; Tian H.; Gui J. Divergent synthesis of antiviral diterpenes wickerols A and B. J. Am. Chem. Soc. 2020, 142, 4690–4695. 10.1021/jacs.9b11838. 32073850

[ref47] Abe F.; Yamauchi T. Oleasides; Novel cardenolides with an unusual framework in Nerium (Nerium 10). Chem. Pharm. Bull. 1979, 27, 1604–1610. 10.1248/cpb.27.1604. 540349

[ref48] Yamauchi T.; Abe F.; Wan A. S. C. Cardenolide Monoglycosides from the Leaves of Cerbera odollam and Cerbera manghas (Cerbera. III). Chem. Pharm. Bull. 1987, 35, 2744–2749. 10.1248/cpb.35.2744.

[ref49] Laphookhieo S.; Cheenpracha S.; Karalai C.; Chantrapromma S.; Rat-a-pa Y.; Ponglimanont C.; Chantrapromma K. Cytotoxic cardenolide glycoside from the seeds of *Cerbera odollam*. Phytochemistry 2004, 65, 507–510. 10.1016/j.phytochem.2003.10.019. 14759549

[ref50] Cao Y.-L.; Zhang M.-H.; Lu Y.-F.; Li C.-Y.; Tang J.-S.; Jiang M.-M. Cardenolides from the leaves of Nerium oleander. Fitoterapia 2018, 127, 293–300. 10.1016/j.fitote.2018.03.004. 29540313

[ref51] Rodríguez J. R.; Castedo L.; Mascareñas J. L. Construction of bridged polycyclic systems via radical cyclizations. uncovering of a novel carbocyclization–ring expansion sequence. Org. Lett. 2001, 3, 1181–1183. 10.1021/ol015643d. 11348189

[ref52] Pakulski Z.; Cmoch P.; Korda A.; Luboradzki R.; Gwardiak K.; Karczewski R. Rearrangements of betulin core. Synthesis of terpenoids possessing the bicyclo[3.3.1]nonane fragment by rearrangement of lupane-type epoxides. J. Org. Chem. 2021, 86, 1084–1095. 10.1021/acs.joc.0c02560. 33353300

[ref53] Frisch M. J.; Trucks G. W.; Schlegel H. B.; Scuseria G. E.; Robb M. A.; Cheeseman J. R.; Scalmani G.; Barone V.; Mennucci B.; Petersson G. A.; Nakatsuji H.; Caricato M.; Li X.; Hratchian H. P.; Izmaylov A. F.; Bloino J.; Zheng G.; Sonnenberg J. L.; Hada M.; Ehara M.; Toyota K.; Fukuda R.; Hasegawa J.; Ishida M.; Nakajima T.; Honda Y.; Kitao O.; Nakai H.; Vreven T.; Montgomery J. A. Jr.; Peralta J. E.; Ogliaro F.; Bearpark M.; Heyd J. J.; Brothers E.; Kudin K. N.; Staroverov V. N.; Kobayashi R.; Normand J.; Raghavachari K.; Rendell A.; Burant J. C.; Iyengar S. S.; Tomasi J.; Cossi M.; Rega N.; Millam J. M.; Klene M.; Knox J. E.; Cross J. B.; Bakken V.; Adamo C.; Jaramillo J.; Gomperts R.; Stratmann R. E.; Yazyev O.; Austin A. J.; Cammi R.; Pomelli C.; Ochterski J. W.; Martin R. L.; Morokuma K.; Zakrzewski V. G.; Voth G. A.; Salvador P.; Dannenberg J. J.; Dapprich S.; Daniels A. D.; Farkas O.; Foresman J. B.; Ortiz J. V.; Cioslowski J.; Fox D. J. Gaussian 09, Rev. A.1; Gaussian Inc.: Wallingford, CT, 2009.

[ref54] Keith T. A. AIMAll, ver. 19.10.12; TK Gristmill Software: Overland Park, KS, 2019. aim.tkgristmill.com.

[ref55] Lehn J. M.; Ourisson G. Syntheses dans la serie du lupane. Bull. Soc. Chim. France 1962, 1133–1136.

[ref56] Wahhab A.; Ottosen M.; Bachelor F. W. The synthesis of nor- and bisnorlupanes. Can. J. Chem. 1991, 69, 570–577. 10.1139/v91-086.

[ref57] Flekhter O. B.; Medvedeva N. I.; Kukovinets O. S.; Spirikhin L. V.; Galkin E. G.; Galin F. Z.; Golovanov D. G.; Pavlova N. I.; Savinova O. V.; Boreko E. I.; Tolstikov G. A. Synthesis and antiviral activity of lupane triterpenoids with modified cycle E. Russ. J. Bioorg. Chem. 2007, 33, 584–588. 10.1134/S1068162007060088. 18173126

[ref58] Vystrčil A.; Křeček V.; Buděšínský M. Elimination reactions of the angular hydroxymethyl group of the lupane skeleton. Collect. Czech. Chem. Commun. 1974, 39, 3131–3143. 10.1135/cccc19743131.

