# Supplementary material for: Oxidative Functionalization of Trinor-18α-olean-17(22)-ene Derivatives. Annulation of the E-Ring by an Intramolecular Aldol Reaction
Source: J Org Chem. 2021 May 25;86(11):7636–47. doi: 10.1021/acs.joc.1c00697 (PMC8279477; doi:10.1021/acs.joc.1c00697)
Supplement: Supplementary file 1 — jo1c00697_si_001.pdf [file jo1c00697_si_001.pdf]

Supporting information

Oxidative functionalization of trinor-18α-olean-17(22)-ene derivatives. Annulation of the E-ring by an intramolecular aldol reaction

Kinga Kuczynska,[,a] Jarosław Jaźwiński,[a] Zbigniew Pakulski,[*,a] Piotr Cmoch,[a] Roman Luboradzki [b]*

[a] Institute of Organic Chemistry, Polish Academy of Sciences, Kasprzaka 44/52, 01-224 Warsaw, Poland
[b] Institute of Physical Chemistry, Polish Academy of Sciences, Kasprzaka 44/52, 01-224 Warsaw, Poland

**Table of contents**	**Page**
Crystal structure determination of compounds **21**, **23**, and **24**	S2-S5
Key HMBC and NOE correlations	S5
DFT calculation – Figures 1S-11S	S6-S9
DFT calculation – atomic coordinates of the key structures	S9-S51
NMR spectra of compounds **2**, **8-14**, **17**, **19**, **20**, **21**, **23**, and **24**	S52-S79

Single crystal X-ray diffraction measurements were carried out on an Agilent Supernova diffractometer at 100K with monochromated Cu Kα radiation (1.54184 A). The data reduction was made by using CrysAlisPRO software.[1] All non-hydrogen atoms were refined as anisotropic while hydrogen atoms were placed in calculated positions and refined in riding mode. CCDC 2055466 (**21**), CCDC 2031957 (**23**), CCDC 2031169 (**24**) contain the supplementary crystallographic data for this paper. These data can be obtained free of charge from The Cambridge Crystallographic Data Centre via www.ccdc.cam.ac.uk/data_request/cif

The structures of compounds **21**, **23**, and **24** were determined on crystals prepared in a chloroform / methanol solvent system by slow evaporation at room temperature.

Using Olex2, structure **21** was solved with olex2.solve structure solution program using Charge Flipping and refined with olex2.refine refinement package using Gauss-Newton minimisation.[2] The crystal was of poor quality [0.09 × 0.03 × 0.01 mm] that a sharp drop in diffraction intensity was observed at high theta angles. Therefore, the measurement was carried out for the theta angle values ranging up to 104.7°. This caused an alert A in the checkCIF report, however the data obtained is absolutely sufficient to determine the correct structure and conformation of the molecule.

The structures **23** and **24** were solved by direct methods and refined on F^2 by full-matrix least-squares by using SHELXS97 and SHELXL97.[3] The fragment of molecule **24** was refined as disordered in two parts with occupancy 0.85 and 0.15 respectively.

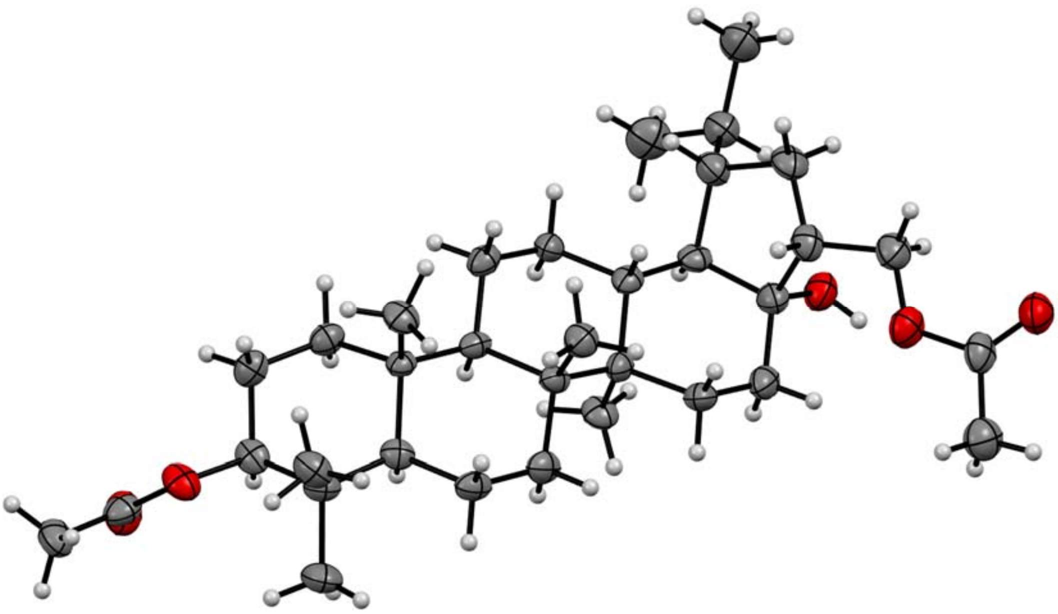

Structure S1. ORTEP diagram of compound **21** (CCDC 2055466). Contour probability level: 50%.

Table S1. Crystal data for **21**.

Compound	KK768B_010
Empirical formula	$C_{34}H_{56}O_5$
Moiety formula	$C_{34}H_{56}O_5$
Formula weight	544.821
CCDC No.	CCDC 2055466
Wavelength	1.54184

[1] Agilent. CrysAlis PRO; Agilent Technologies, Yarnton, England, **2011**.
[2] Bourhis, L. J.; Dolomanov, O. V.; Gildea, R. J.; Howard, J. A. K.; Puschmann, H., The anatomy of a comprehensive constrained, restrained refinement program for the modern computing environment - Olex2 dissected, *Acta Crystallogr., Sect. A: Found. Crystallogr.* **2015**, *A71*, 59-75.
[3] Sheldrick, G. M. A short history of SHELX. *Acta Crystallogr., Sect. A: Found. Crystallogr.* **2008**, *A64*, 112-122.

Crystal system	orthorhombic	
Space group	$P2_12_12_1$	
Unit cell dimensions	a = 6.6009(3)Å	
	b = 13.0155(8)Å	
	c = 35.547(2)Å	
Volume	3054.0(3)Å^3	
Z	4	
Density Calc.	1.185g/cm^3	
Absorption coefficient	0.604 mm^{-1}	
F(000)	1200	
Crystal	Colourless needle	
Crystal size	0.09 × 0.03 × 0.01 mm	
Index ranges	$-6 \leq h \leq 6$, $-13 \leq k \leq 12$, $-32 \leq l \leq 36$	
Reflections collected (all / independent)	7403 / 3287 [R_{int} = 0.0545]	
Absorption correction	multi-scan	
Refinement method	Full-matrix least-squares on F^2	
Restraints / parameters	0 / 362	
Goodness-of-fit on F^2	1.0502	
Final R indices [$F^2 > 2\sigma(F^2)$]	R_1 = 0.0540, wR_2 = 0.1214	
R indices (all data)	R_1 = 0.0715, wR_2 = 0.1361	

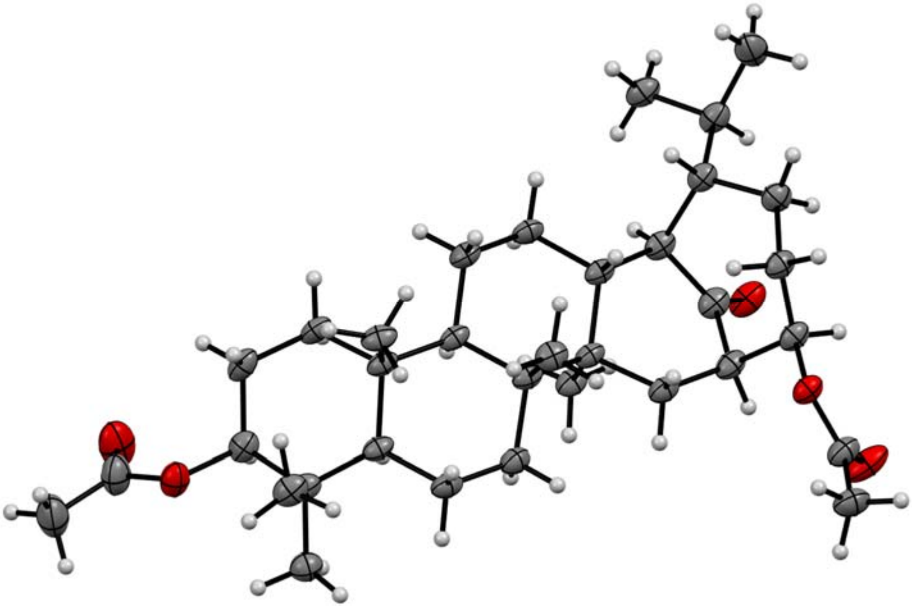

Structure S2. ORTEP diagram of compound **23** (CCDC 2031957). Contour probability level: 50%.

Table S2. Crystal data for **23**.

Compound	kk767a	
Empirical formula	$C_{34}H_{54}O_5$	
Moiety formula	$C_{34}H_{54}O_5$	
Formula weight	542.77	
CCDC No.	CCDC 2031957	
Wavelength	1.54184	
Crystal system	monoclinic	
Space group	$P2_1$	
Unit cell dimensions	a = 14.7524(6)Å	
	b = 7.2005(2)Å	β = 101.562(4)°

	c = 14.8903(6)Å	
Volume	1549.62(10)) Å^3	
Z	2	
Density Calc.	1.163 g/cm^3	
Absorption coefficient	0.595 mm^{-1}	
F(000)	596	
Crystal	Colorless needle	
Crystal size	0.5 × 0.05 × 0.01 mm	
Index ranges	-16≤ h ≤ 18, -8 ≤ k ≤ 6, -18 ≤ l ≤18	
Reflections collected (all / independent)	9447 / 4782 [R_{int} = 0.0508]	
Absorption correction	multi-scan	
Refinement method	Full-matrix least-squares on F^2	
Restraints / parameters	1 / 361	
Goodness-of-fit on F^2	1.075	
Final R indices [$F^2 > 2\sigma(F^2)$]	R_1 = 0.0428, wR_2 = 0.1179	
R indices (all data)	R_1 = 0.0597, wR_2 = 0.1242	

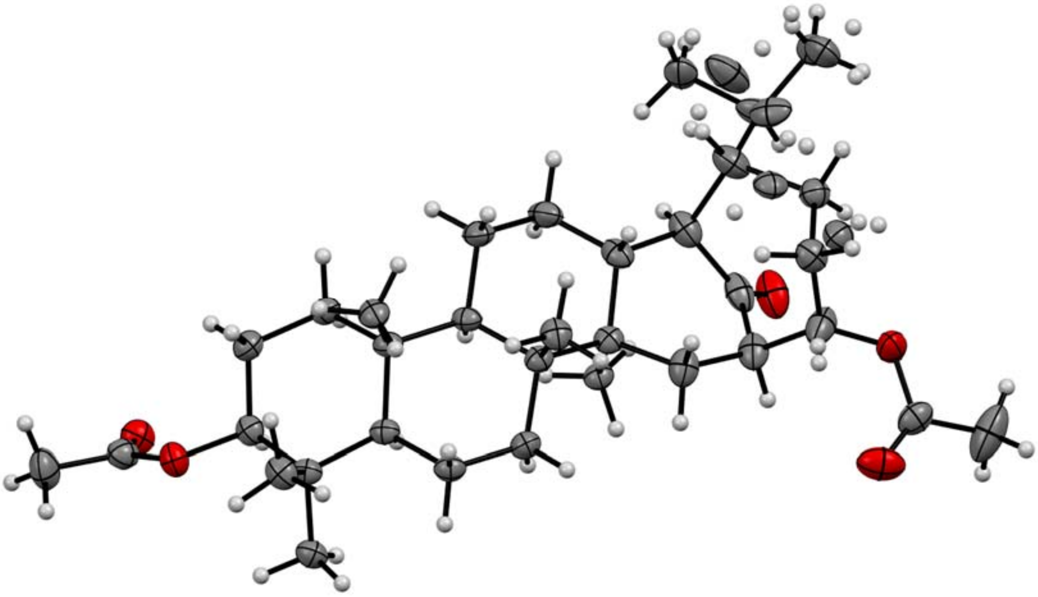

Structure S3. ORTEP diagram of compound **24** (CCDC 2031169). Contour probability level: 50%.

Table S3. Crystal data for **24**.

Compound	kk767b	
Empirical formula	$C_{34}H_{54}O_5$	
Moiety formula	$C_{34}H_{54}O_5$	
Formula weight	542.77	
CCDC No.	CCDC 2031169	
Wavelength	1.54184	
Crystal system	orthorhombic	
Space group	$P2_12_12_1$	
Unit cell dimensions	a = 8.12000(10) Å	
	b = 11.32180(10) Å	
	c = 32.5914(4) Å	
Volume	2996.23(6) Å^3	
Z	4	
Density Calc.	1.203 g/cm^3	

Absorption coefficient	0.616 mm^{-1}
F(000)	1192
Crystal	Colorless plate
Crystal size	0.4 × 0.3 × 0.02 mm
Index ranges	$-9 \leq h \leq 6$, $-13 \leq k \leq 13$, $-38 \leq l \leq 39$
Reflections collected (all / independent)	27660 / 5655 [R_{int} = 0.0370]
Absorption correction	multi-scan
Refinement method	Full-matrix least-squares on F^2
Restraints / parameters	0 / 409
Goodness-of-fit on F^2	1.056
Final R indices [$F^2 > 2\sigma(F^2)$]	R_1 = 0.0382, wR_2 = 0.0929
R indices (all data)	R_1 = 0.0423, wR_2 = 0.0943

Table S4. Key HMBC (------) and NOE (------) correlations.

ZP 4803

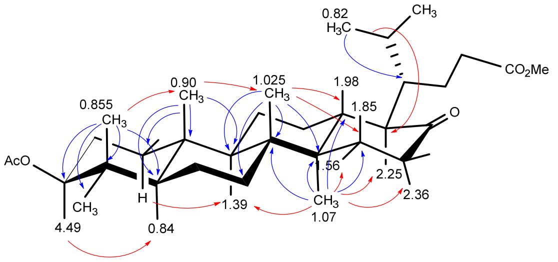

KK 602

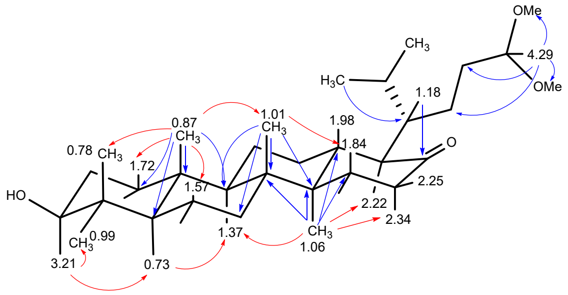

BT 1021B

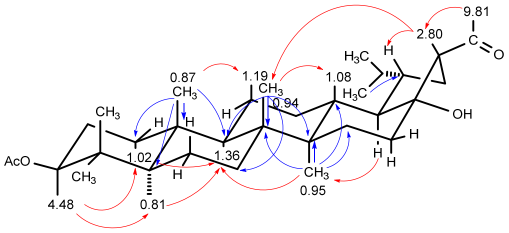

KK 700C

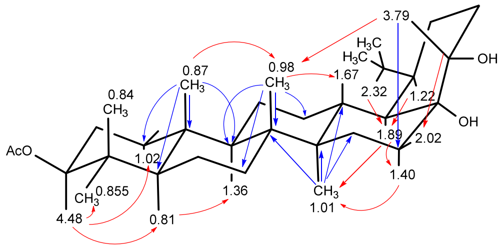

ZP 4741

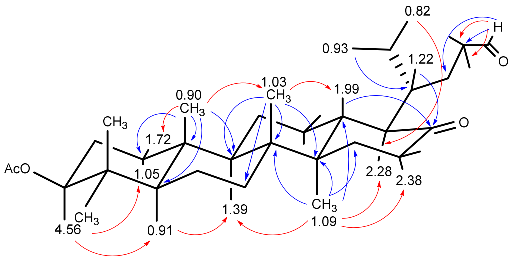

DFT calculations:

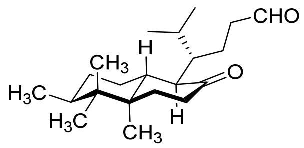

Figure 1S. A simplified structure used in the preliminary calculations

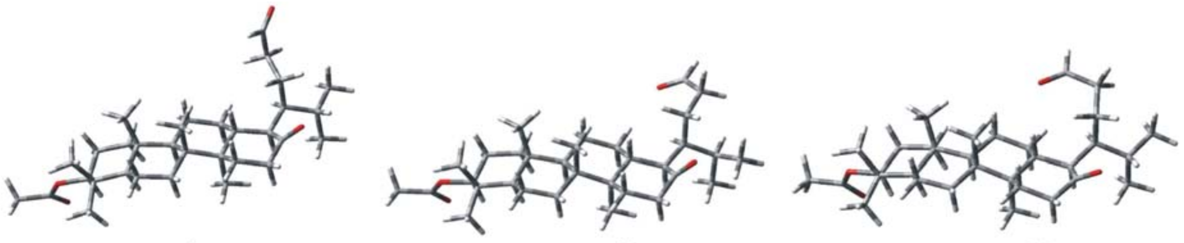

I
-1552.0802

II
-1552.0797

III
-1552.0810

Figure 2S. Three the most populated rotamers of **10** (0.22, 0.13, and 0.48 molar fractions, respectively).

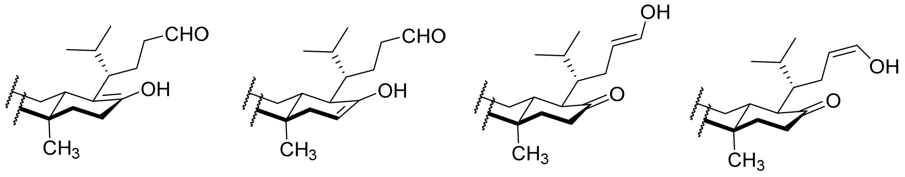

-1552.0655 -1552.0653 -1552.0668 -1552.0680

Figure 3S. Enol structures used as input in the calculations; the structures were constructed starting from **III** (Fig.2S). Boltzmann distribution revealed molar fractions of 0.05, 0.05, 0.20, and 0.70, respectively, if only these four tautomers were considered. However, the molar fractions were much smaller than 0.01 when **III** was included. Molecular energy differences between the above tautomers and **III** vary from 34 to 41 kJ/mol.

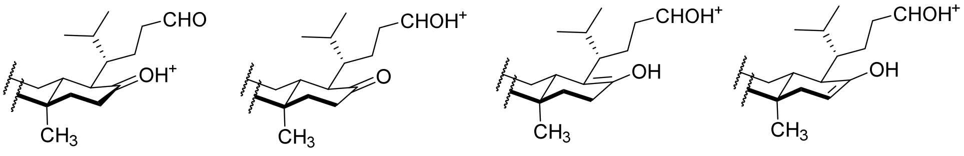

-1552.4322 -1552.4069 -1552.3905 -1552.3864

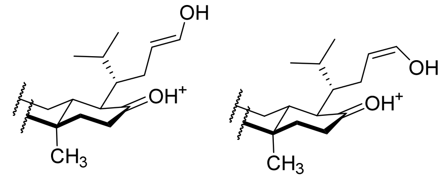

-1552.4138 -1552.4120

Figure 4S. Molecular energies of protonated compounds; the input structures were constructed starting from **III** (Fig.2S). The first structure is the most populated (x≈1)

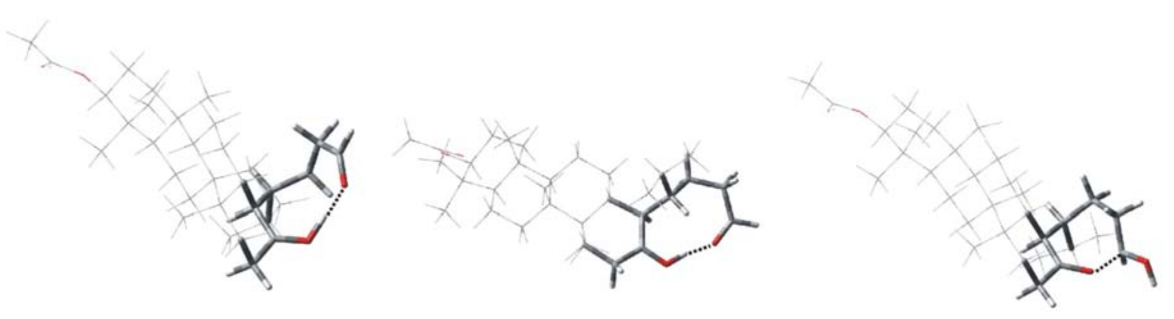

IV
-1552.4370

V
-1552.4458

VI
-1552.4394

Figure 5S. The rotamers stabilized by hydrogen bonds or the O···C interaction (dotted lines). Bond lengths are as follows: 1.032 and 1.514 Å (O···H, **IV**), 1.032 and 1.555 Å (O···H, **V**), 1.591 Å (=O···CH(OH$^+$, **VI**).

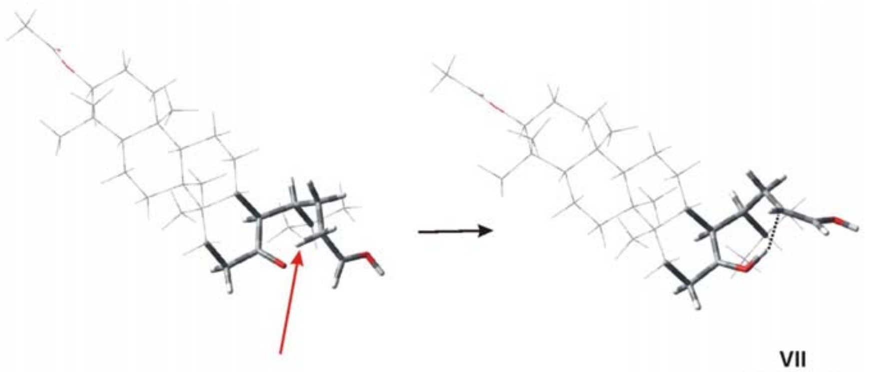

VII
-1552.4307

Figure 6S. The formation of enol during structure optimization. The H atom from CH_2 group (red arrow) moved to C=O group, forming the rotamer **VII** (enol) stabilized by OH···-CH= interaction (, dotted line, d_{OH} 1.022 Å, d_{HC} 1.795 Å)

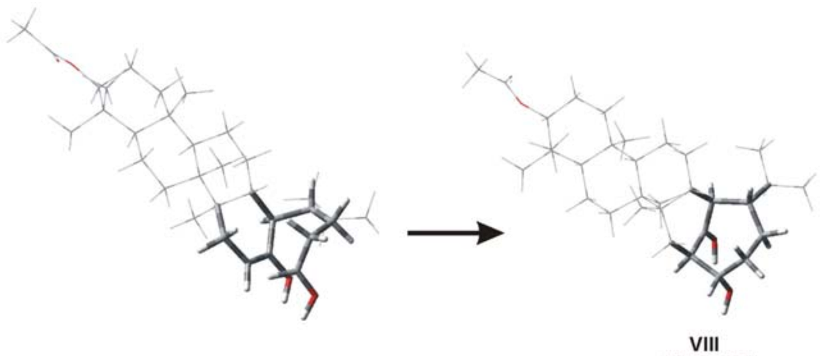

VIII
-1552.4407

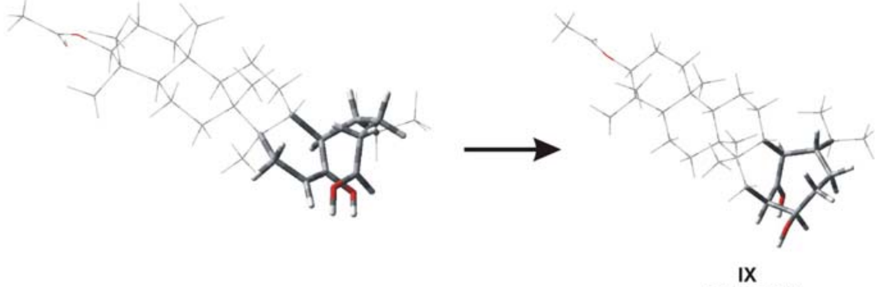

IX
-1552.4319

Figure 7S. The transformation of **10** (enole form) to cyclic product **22**. Depending on the orientation of CH(=OH$^+$) group, two configurations of CH(OH) center were obtained.

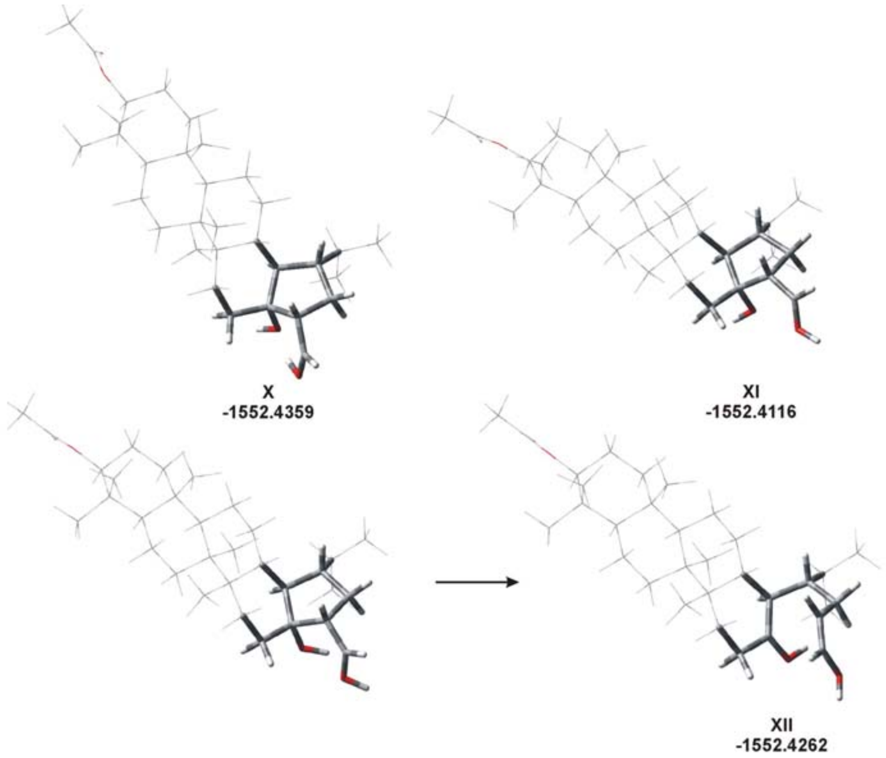

Figure 8S. Two structures, **11** with and without hydrogen bonds (on the top). Below, the structure with opposite hydrogen bond patterns that in **X**. During optimization, the isomer **XII** with the open chain fragment was obtained.

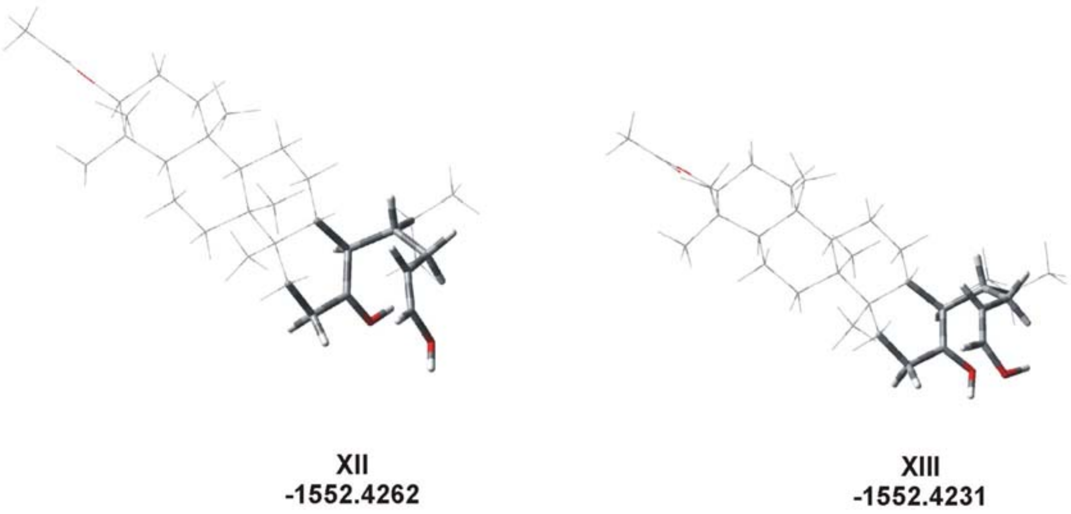

Figure 9S. Two arrangements of hydrogen bond pattern obtained during various optimizations. In both structures, O-H bonds are parallel.

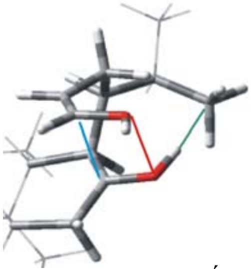

Figure 10S. Weak interactions in **XII**, O$\cdots$O (red line, d_{OO} of 2.914 Å), C$\cdots$C (blue line, d_{CC} of 3.035 Å), and H$\cdots$C (green line, d_{HC} of 2.275 Å).

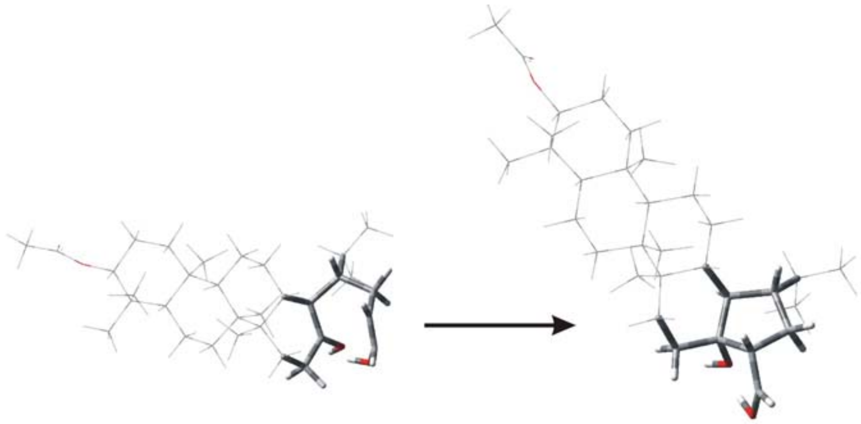

Figure 11S. Formation of **11** from a linear rotamer (cf. Fig. 8S).

Atomic coordinates of the key structures. Structures have been optimized at the B3LYP/6-31G(2d,p) theory level; the molecular energies have been estimated at the B3LYP/6-311++G(2d,p) theory level.

I (Fig.2S), E = -1552.0802

0 1

C	0.00000000	0.00000000	0.00000000
C	1.54654447	0.00000000	0.00000000
C	1.99058940	1.50516543	0.00000000
C	1.39502281	2.43392187	1.11498412
C	-0.15025455	2.28314783	1.06202134
C	-0.62869066	0.82761398	1.11278400
H	-0.35210133	0.38447222	-0.96288258
H	-0.61618760	2.82967194	1.88798071
H	-0.52468966	2.74209045	0.13689034
H	-0.40457998	0.36889792	2.08175519
H	-1.71620847	0.79821156	0.99638087
H	1.55426156	1.90017294	-0.93243628
C	1.85305782	2.08296457	2.55403338
H	2.86349734	1.67528911	2.60355580
H	1.82172152	2.96177419	3.20229725
H	1.19497337	1.34040443	3.00942152
C	1.75134374	3.91931377	0.69866210
H	1.23943541	4.01951442	-0.26569905
C	3.50454035	1.72028297	-0.13788201
H	3.91688797	1.04823006	-0.89564457
H	4.02409541	1.47149434	0.79373830
C	3.81105288	3.16078265	-0.56735862
H	3.37722366	3.29808064	-1.56285848
H	4.89406427	3.27978519	-0.68193742
C	3.26253240	4.24025023	0.40138667
C	2.00179190	-0.66627397	-1.31914670
H	3.07900935	-0.85076014	-1.33511119
H	1.50583653	-1.63356057	-1.43629862
H	1.74979899	-0.04984762	-2.18931925
C	2.11825542	-0.83549927	1.16314140
H	1.72625144	-0.54424447	2.13769107
H	1.87568337	-1.89072952	1.01586126
H	3.20809981	-0.74750114	1.20152212
O	-0.45120108	-1.37648176	0.10220555
C	-1.62917408	-1.68451111	-0.48181208
O	-2.32135398	-0.89234606	-1.07356085
C	4.13114906	4.18708417	1.68282972
H	5.18250720	4.37126285	1.45188531
H	3.83123217	4.90670742	2.44657078
H	4.09007212	3.20380306	2.14547116
C	3.36169858	5.70106757	-0.27646009

C	1.13295382	4.99662681	1.60458692
H	0.07141248	4.79259815	1.76919760
H	1.59923662	4.98915052	2.59671405
C	2.72530201	6.76614850	0.69005544
C	1.26699888	6.40293079	1.00537005
H	0.83658014	7.11875359	1.71019605
H	0.66071457	6.47807146	0.09270902
C	2.65528245	5.71873964	-1.65938545
H	2.48801405	6.73682281	-2.01593801
H	3.26684436	5.22250644	-2.41746595
H	1.67985580	5.23160480	-1.65811795
H	3.28444822	6.70253003	1.63095764
C	2.88473264	8.24722115	0.18778604
H	2.32740681	8.30770553	-0.75447139
C	4.84101150	6.09597368	-0.51231586
H	5.39436471	6.06737172	0.43042127
H	5.32538230	5.37949500	-1.18264840
C	5.00134349	7.51287884	-1.09140683
H	4.54226675	7.57638136	-2.08520400
C	4.34418796	8.52725143	-0.17790748
H	6.05516924	7.78236568	-1.19463765
C	2.12897018	9.22730483	3.73673675
H	1.37062115	10.01242008	3.79243976
C	2.29619570	9.32708575	1.14168766
H	1.26113749	9.02392615	1.33996172
C	3.04508825	9.38222713	2.50680824
H	3.81275854	8.60329092	2.55122322
H	3.59755922	10.32311656	2.58205418
O	4.96081316	9.48173990	0.24927394
C	2.91783989	9.23243188	5.02440788
O	2.72090599	9.97244387	5.95558061
H	3.73909702	8.47701794	5.06388118
C	2.18307049	10.73960948	0.49141339
C	1.41043105	11.70517454	1.40561114
H	1.35295187	12.69889791	0.94967965
H	0.38193079	11.35897507	1.56936498
H	1.88291086	11.82625158	2.38383334
C	-1.94844104	-3.14788343	-0.29241256
H	-2.91102198	-3.37124281	-0.75064954
H	-1.16720496	-3.76397609	-0.74703780
H	-1.97455181	-3.39144537	0.77342339
C	1.51126744	10.71817155	-0.89088726
H	2.11870646	10.21767454	-1.65003349
H	0.53420848	10.21908831	-0.85542840
H	1.34086849	11.74051992	-1.24358552
H	3.19868125	11.12803443	0.36912078
H	1.60757900	8.25990061	3.68136526

II (Fig. 2S) E = -1552.0797

0 1

C	0.00000000	0.00000000	0.00000000
C	1.54642065	0.00000000	0.00000000
C	1.98870736	1.50594377	0.00000000
C	1.39188644	2.43467947	1.11389923
C	-0.15346435	2.28060522	1.06534277
C	-0.62875235	0.82400090	1.11531007
H	-0.35188190	0.38724750	-0.96189018
H	-0.61786721	2.82561170	1.89279618
H	-0.53155579	2.73991524	0.14199669
H	-0.40023424	0.36405710	2.08263063
H	-1.71644211	0.79139345	1.00133600
H	1.55303552	1.90017875	-0.93303006
C	1.85328837	2.08932406	2.55262698

H	2.86971255	1.69678449	2.60382013
H	1.80699744	2.96841106	3.19936519
H	1.20484891	1.33812996	3.00765757
C	1.74203427	3.92011684	0.69366212
H	1.22132256	4.01832867	-0.26588545
C	3.50260751	1.72272421	-0.13498600
H	3.91951803	1.04671778	-0.88695037
H	4.01789730	1.48116349	0.80061453
C	3.81008599	3.16155655	-0.57073320
H	3.38779810	3.28961420	-1.57222434
H	4.89418454	3.28208760	-0.67492487
C	3.25001613	4.24696863	0.38444351
C	2.00155035	-0.66743128	-1.31823535
H	3.07864327	-0.85246135	-1.33308949
H	1.50494330	-1.63457294	-1.43457933
H	1.75040167	-0.05185702	-2.18925019
C	2.11900196	-0.83522869	1.16325230
H	1.73371466	-0.53939244	2.13922151
H	1.87118370	-1.88988728	1.01966931
H	3.20938803	-0.75146138	1.19570115
O	-0.45323006	-1.37697358	0.09787480
C	-1.63194528	-1.68109534	-0.48530616
O	-2.32243505	-0.88772965	-1.07764254
C	4.11860128	4.21405090	1.66733522
H	5.16544767	4.42841473	1.44207749
H	3.79630323	4.91997151	2.43406881
H	4.10440453	3.22645044	2.12402269
C	3.33017455	5.70078871	-0.31052916
C	1.13497566	4.99537541	1.60895781
H	0.07508164	4.79334329	1.78567271
H	1.61039873	4.98080605	2.59614276
C	2.70639130	6.77224891	0.65950649
C	1.25882908	6.41082879	1.02773602
H	0.86800169	7.11039878	1.76937495
H	0.61714012	6.50561703	0.14087671
C	2.59700049	5.70503122	-1.67850107
H	2.49148311	6.71611985	-2.07555274
H	3.15624119	5.13902523	-2.42758012
H	1.59086523	5.28812153	-1.63568507
H	3.29457768	6.73020422	1.58380751
C	2.85189116	8.25106044	0.12353489
H	2.37749851	8.26229074	-0.86370846
C	4.80675917	6.08903075	-0.58500866
H	5.39451494	6.01631908	0.33362129
H	5.25681147	5.39479038	-1.30143945
C	4.97418052	7.52871122	-1.10851597
H	4.50093555	7.64079416	-2.09071614
C	4.33984406	8.49044645	-0.12309651
H	6.03146915	7.78548219	-1.20810767
C	1.90714247	10.36330664	3.35646769
H	2.47362468	10.51664934	4.28887991
C	2.70505939	9.47577359	2.39576272
H	2.76768520	8.47965258	2.84696880
H	3.72740160	9.84966321	2.33401245
O	5.00753680	9.32119752	0.45869218
C	0.56139795	9.83700092	3.78717839
O	0.09618677	8.76220312	3.48980159
H	-0.00928367	10.52562104	4.45216873
C	1.88793761	10.69975930	0.24672930
C	1.18067357	10.52825566	-1.10772972
H	0.82088582	11.49541583	-1.47399840
H	1.85279435	10.12882173	-1.87552371
H	0.31707871	9.85795545	-1.03388817

C	-1.95690552	-3.14335838	-0.29467040
H	-2.92064854	-3.36295164	-0.75236352
H	-1.17858717	-3.76316658	-0.74924815
H	-1.98366736	-3.38587159	0.77138091
C	3.09708408	11.63541451	0.09820868
H	3.61551541	11.79856818	1.04641767
H	3.83568400	11.24781298	-0.60620749
H	2.76096217	12.61083725	-0.27211961
H	1.16682554	11.22737810	0.88660779
H	1.75393185	11.38017840	2.97038496
C	2.11888213	9.33319634	0.97109473
H	1.10290151	8.94696190	1.09243870

III (Fig. 2S) E = -1552.0810

0 1

C	0.00000000	0.00000000	0.00000000
C	1.54618155	0.00000000	0.00000000
C	1.98617191	1.50684957	0.00000000
C	1.39178675	2.43009266	1.11941777
C	-0.15334580	2.27395929	1.07830716
C	-0.62862958	0.81719330	1.12040243
H	-0.35161712	0.39348112	-0.95909237
H	-0.61256171	2.81299558	1.91247237
H	-0.53632091	2.73932759	0.15986217
H	-0.40187101	0.35188389	2.08565518
H	-1.71642684	0.78552071	1.00504497
H	1.54487386	1.90092765	-0.93054730
C	1.86369542	2.08674005	2.55539538
H	2.87890411	1.68995240	2.59708392
H	1.83062965	2.96947379	3.19799203
H	1.21679524	1.33977794	3.02022657
C	1.73690299	3.91632434	0.70015848
H	1.22167597	4.00863586	-0.26323141
C	3.49883928	1.72908629	-0.14097857
H	3.91323457	1.05914924	-0.89963450
H	4.01876597	1.48205976	0.79037189
C	3.80372854	3.17248904	-0.56651303
H	3.37815298	3.31110948	-1.56580433
H	4.88776770	3.29316370	-0.67190677
C	3.24578925	4.24858341	0.39973583
C	2.00237483	-0.66647211	-1.31836629
H	3.08039513	-0.84596886	-1.33412140
H	1.51023337	-1.63599390	-1.43405205
H	1.74733525	-0.05141148	-2.18861588
C	2.11768878	-0.83473449	1.16418586
H	1.73318038	-0.53503259	2.13906279
H	1.86762315	-1.88942452	1.02366332
H	3.20827833	-0.75381752	1.19674331
O	-0.45541038	-1.37709142	0.08957768
C	-1.62618870	-1.67947910	-0.50995127
O	-2.30790592	-0.88557458	-1.11187204
C	4.11298737	4.21499019	1.68321218
H	5.15551390	4.44861186	1.45502752
H	3.77917873	4.91017829	2.45497499
H	4.11706483	3.22334601	2.13170064
C	3.32656730	5.70671575	-0.28247856
C	1.11167411	4.98959135	1.60569037
H	0.05067021	4.77778584	1.76619469
H	1.57507903	4.99853769	2.59743879
C	2.69387097	6.76439104	0.68991791
C	1.23486187	6.39397992	1.00210876
H	0.80291895	7.10345645	1.71104491
H	0.62865253	6.46485603	0.08827944

C	2.60995482	5.71662857	-1.66152947
H	2.38133350	6.73199937	-1.99250877
H	3.24109519	5.27116724	-2.43575984
H	1.66360799	5.17661402	-1.66578998
H	3.24217726	6.69781051	1.63303952
C	2.83988253	8.24915512	0.19999230
H	2.22737800	8.32551574	-0.70741716
C	4.79968104	6.11732282	-0.53496022
H	5.35696369	6.12304752	0.40606600
H	5.29493400	5.39294866	-1.18933905
C	4.92512735	7.51837963	-1.15254046
H	4.45152528	7.54630585	-2.14140496
C	4.26661665	8.55678816	-0.26821252
H	5.97106714	7.80706387	-1.28322791
C	2.49793091	9.50025726	3.80597762
H	3.10587227	10.01454971	4.56803495
C	2.31723913	9.31752278	1.20780893
H	1.33525528	8.96607532	1.54604771
C	3.23468960	9.44261765	2.46120892
H	3.92908127	8.60021037	2.50718531
H	3.86217458	10.32932428	2.35480629
O	4.85798058	9.57186491	0.04140068
C	2.15009717	8.16822259	4.42283337
O	2.50173302	7.08721339	4.01249519
H	1.53481417	8.23894835	5.34873596
C	2.05379908	10.70269915	0.53820720
C	1.46143469	11.71939567	1.52875517
H	1.29393509	12.67952226	1.02967251
H	0.49158517	11.38173431	1.91693296
H	2.11961860	11.91096313	2.38021018
C	-1.95705678	-3.14089075	-0.32250272
H	-2.90216279	-3.36435350	-0.81583838
H	-1.16083871	-3.76403201	-0.73958563
H	-2.02731732	-3.37460795	0.74368101
C	1.12289425	10.61297270	-0.68311155
H	1.57308591	10.08236200	-1.52615228
H	0.18056163	10.10914166	-0.43262307
H	0.87050861	11.61677875	-1.04040316
H	3.02085806	11.08996884	0.20319014
H	1.57194171	10.08782145	3.75957210

tatomer E = -1552.0655 (Fig.3S),

0 1

C	0.00000000	0.00000000	0.00000000
C	1.54618058	0.00000000	0.00000000
C	1.98952156	1.50500586	0.00000000
C	1.39469640	2.43298496	1.11738103
C	-0.15050579	2.27943933	1.06930277
C	-0.62745916	0.82312792	1.11649158
H	-0.35362293	0.38625386	-0.96149773
H	-0.61262100	2.82209928	1.89960837
H	-0.52821550	2.74273528	0.14755365
H	-0.39773872	0.36203677	2.08304477
H	-1.71540083	0.79112883	1.00356609
H	1.55297752	1.90052448	-0.93199432
C	1.86010783	2.08630979	2.55507282
H	2.86802039	1.67196960	2.59824109
H	1.84257212	2.97081030	3.19607350
H	1.20023905	1.35194943	3.02227174
C	1.74985842	3.91623707	0.69740348
H	1.25378308	4.00736770	-0.27616289
C	3.50418425	1.71845953	-0.14042140
H	3.91324124	1.05118419	-0.90449770

H	4.02384298	1.46023182	0.78857134
C	3.81616406	3.16216863	-0.55595709
H	3.38937774	3.31352442	-1.55309410
H	4.90073492	3.27727545	-0.66326620
C	3.26602021	4.23406837	0.41837077
C	2.00195849	-0.66979469	-1.31663123
H	3.07934187	-0.85276101	-1.33098895
H	1.50688123	-1.63797230	-1.43091635
H	1.74990122	-0.05639376	-2.18885281
C	2.11512467	-0.83411033	1.16571465
H	1.72128244	-0.53995995	2.13884626
H	1.87269289	-1.88989550	1.02063336
H	3.20491520	-0.74633534	1.20645326
O	-0.45177659	-1.37801214	0.10007758
C	-1.62681480	-1.68785664	-0.48604628
O	-2.31857605	-0.89979454	-1.08414552
C	4.12977759	4.17634072	1.70295050
H	5.16948938	4.42516902	1.47868607
H	3.79268253	4.85558024	2.48770113
H	4.14089434	3.17589602	2.13063084
C	3.36895780	5.69229867	-0.24348962
C	1.10930903	5.00289750	1.57958153
H	0.04467191	4.79586275	1.72070998
H	1.55391378	5.01106710	2.58180570
C	2.73563910	6.74763810	0.72311774
C	1.25500987	6.40440149	0.96847083
H	0.80024511	7.12181558	1.65600893
H	0.68084490	6.46971265	0.03646102
C	2.67082669	5.73773049	-1.63072422
H	2.46314246	6.77081890	-1.91776476
H	3.30699025	5.30523881	-2.40887657
H	1.71958889	5.20658706	-1.66130397
H	3.22023773	6.62713149	1.70030716
C	3.02108169	8.20521341	0.33026051
C	4.83918231	6.11806871	-0.46736689
H	5.35485799	6.19682061	0.49340804
H	5.37825919	5.37478164	-1.06337890
C	4.93081058	7.48381847	-1.15551983
H	4.71368278	7.40680806	-2.23047894
C	4.02495162	8.49373729	-0.51217775
H	5.96685184	7.84930037	-1.08536974
C	2.80672734	10.32988929	3.43035320
H	3.44338296	11.05434815	3.96308607
C	2.29728135	9.32908922	1.08207425
H	1.57481486	8.85362342	1.75117825
C	3.29648116	10.11082629	1.99631712
H	4.23702846	9.55765787	2.04757479
H	3.53512151	11.07865939	1.54656808
O	4.31606585	9.81309566	-0.81001314
C	2.78321128	9.09153390	4.29259849
O	3.13476743	7.98824180	3.94828451
H	2.41300066	9.26168208	5.33025467
C	1.46964335	10.27435204	0.15820731
C	0.71598746	11.35510550	0.94855156
H	0.14036417	11.99267176	0.26876318
H	0.00332987	10.90800269	1.65390986
H	1.38645193	12.00949158	1.51325074
C	-1.94709421	-3.15081417	-0.29074390
H	-2.90515078	-3.37759146	-0.75681617
H	-1.16113139	-3.76961954	-0.73333447
H	-1.98385537	-3.38738494	0.77640363
C	0.47207408	9.50114019	-0.71516606
H	0.97235757	8.75194578	-1.33335306

H	-0.27751638	8.98555629	-0.10234615
H	-0.06513267	10.18371675	-1.38285565
H	2.17886827	10.77715438	-0.50714920
H	1.80010318	10.76896749	3.47197103
H	5.10312036	9.83465932	-1.36343275

tautomer E = -1552.0653 (Fig.3S),

0 1

C	0.00000000	0.00000000	0.00000000
C	1.54619375	0.00000000	0.00000000
C	1.98921574	1.50522580	0.00000000
C	1.39336918	2.43415134	1.11431521
C	-0.15236963	2.27943928	1.06695612
C	-0.62871465	0.82305282	1.11599224
H	-0.35289385	0.38681369	-0.96144006
H	-0.61560101	2.82425799	1.89534053
H	-0.53067930	2.73955131	0.14401538
H	-0.40068354	0.36202591	2.08295193
H	-1.71652734	0.79062400	1.00151818
H	1.55354186	1.89921870	-0.93318371
C	1.85607685	2.08577036	2.55222443
H	2.87543746	1.69977774	2.60093561
H	1.80551294	2.96259196	3.20188426
H	1.21373568	1.32784056	3.00550611
C	1.74230664	3.92057505	0.69550684
H	1.20633116	4.02825570	-0.25515606
C	3.50377741	1.72417807	-0.13726734
H	3.92390328	1.03967778	-0.87985867
H	4.02112858	1.50116698	0.80199335
C	3.79449130	3.15933321	-0.59365105
H	3.35065464	3.27366137	-1.58741076
H	4.87511155	3.29107508	-0.72063477
C	3.24540296	4.24779505	0.36235498
C	2.00184184	-0.66819204	-1.31757690
H	3.07864236	-0.85471023	-1.33033980
H	1.50384002	-1.63449784	-1.43514862
H	1.75372280	-0.05173843	-2.18869272
C	2.11682425	-0.83522055	1.16396449
H	1.72427329	-0.54252935	2.13794781
H	1.87514012	-1.89120112	1.01818518
H	3.20656297	-0.74629137	1.20329297
O	-0.45258581	-1.37801193	0.09829134
C	-1.62770939	-1.68547400	-0.48876189
O	-2.31828669	-0.89566771	-1.08606246
C	4.13112255	4.22698174	1.63533513
H	5.18839025	4.31314412	1.37268360
H	3.90575280	5.02327907	2.34555101
H	4.02796034	3.28583853	2.17175877
C	3.31175735	5.69322457	-0.34093999
C	1.15537044	4.99544884	1.62780955
H	0.10623088	4.77980830	1.85030384
H	1.67788961	5.00825376	2.59045152
C	2.68770074	6.76346930	0.61412208
C	1.24707632	6.38862175	0.99841171
H	0.83866597	7.12343864	1.70005690
H	0.60188289	6.43273734	0.11061958
C	2.58179879	5.70659607	-1.71017409
H	2.56004958	6.71838994	-2.11917096
H	3.10258524	5.08899965	-2.44616947
H	1.55001894	5.35606421	-1.65970420
H	3.27808977	6.75140870	1.53439585
C	2.75308016	8.22827481	0.08830405
H	1.90286493	8.38643705	-0.58748604

C	4.78127107	6.11243759	-0.61584532
H	5.40320110	5.94786816	0.27070807
H	5.20657140	5.47423423	-1.39964298
C	4.89863033	7.55159548	-1.04100766
C	3.99278533	8.48119959	-0.73263150
H	5.76376640	7.83295296	-1.64154232
C	3.95632460	9.73320369	3.45753286
H	4.90541812	10.16914797	3.80909405
C	2.60625012	9.25900170	1.27329621
H	1.97990904	8.76888076	2.02794196
C	3.98165844	9.53138973	1.93871429
H	4.64889671	8.68883396	1.73470975
H	4.45005219	10.40020280	1.46709406
O	4.08397985	9.78586296	-1.16026598
C	3.76354103	8.48402025	4.28086856
O	3.69149044	7.35675600	3.85248526
H	3.70286372	8.66707181	5.37842163
C	1.83559155	10.53992481	0.82589310
C	1.90411146	11.70752968	1.82328408
H	1.36993663	12.57391907	1.41846877
H	1.42287786	11.45394369	2.77634267
H	2.92844237	12.02820083	2.03082455
C	-1.95066311	-3.14817440	-0.29573257
H	-2.90631302	-3.37359286	-0.76741127
H	-1.16283870	-3.76802324	-0.73342065
H	-1.99439357	-3.38462913	0.77120388
C	0.35399536	10.23268729	0.54862816
H	0.21305665	9.44049703	-0.19092846
H	-0.15676741	9.92036322	1.46809448
H	-0.16057840	11.12391009	0.17395694
H	2.29541227	10.88405695	-0.10621196
H	3.19379774	10.45119235	3.78421464
H	4.88283736	9.87615686	-1.69395694

tautomer E = -1552.0668 (Fig. 3S),

0 1

C	0.00000000	0.00000000	0.00000000
C	1.54657305	0.00000000	0.00000000
C	1.99158584	1.50513936	0.00000000
C	1.39608028	2.43340828	1.11494806
C	-0.14901329	2.28376396	1.05943244
C	-0.62902925	0.82895045	1.11135279
H	-0.35214021	0.38270655	-0.96356091
H	-0.61577868	2.83194409	1.88365811
H	-0.52096245	2.74211518	0.13292709
H	-0.40542154	0.37101377	2.08087704
H	-1.71654183	0.79979069	0.99428194
H	1.55612705	1.89962723	-0.93315889
C	1.85280453	2.08130361	2.55426396
H	2.85965614	1.66477616	2.60185985
H	1.83148967	2.96146849	3.20070602
H	1.18945268	1.34520189	3.01286876
C	1.75345431	3.91930124	0.70243147
H	1.24314247	4.02230523	-0.26285997
C	3.50586659	1.71958830	-0.13748294
H	3.91727562	1.04880000	-0.89710325
H	4.02513774	1.46792993	0.79362358
C	3.81389142	3.16143043	-0.56267555
H	3.38024231	3.30260630	-1.55789627
H	4.89719783	3.28000969	-0.67723692
C	3.26580552	4.23916871	0.40846450
C	2.00133659	-0.66655055	-1.31909898
H	3.07867001	-0.85029976	-1.33549263

H	1.50593556	-1.63426350	-1.43602927
H	1.74888157	-0.05069026	-2.18960227
C	2.11799057	-0.83616656	1.16281817
H	1.73198455	-0.54038805	2.13866730
H	1.86943912	-1.89052101	1.01888384
H	3.20843848	-0.75378798	1.19630676
O	-0.45144592	-1.37691312	0.10414787
C	-1.63003810	-1.68594523	-0.47668758
O	-2.32383661	-0.89587744	-1.06959613
C	4.13080952	4.18005016	1.69227525
H	5.18240009	4.36843081	1.46537339
H	3.82555770	4.89164893	2.46123828
H	4.09210685	3.19288168	2.14713075
C	3.36983281	5.70136222	-0.26460727
C	1.13375364	4.99539939	1.60900165
H	0.07125433	4.79083845	1.76906390
H	1.59635735	4.99259812	2.60254257
C	2.73056550	6.75997697	0.70405851
C	1.27034787	6.40102245	1.01118063
H	0.84917230	7.11420312	1.72260591
H	0.66566257	6.48110254	0.09755923
C	2.67136551	5.72476685	-1.65103380
H	2.50081522	6.74506466	-2.00056291
H	3.28821383	5.23631957	-2.41040735
H	1.69820131	5.23336103	-1.65719419
H	3.27993732	6.69126460	1.64794023
C	2.89273035	8.24598852	0.21927501
H	2.33936152	8.32233412	-0.72593311
C	4.85030283	6.09754828	-0.49055376
H	5.39816496	6.06583199	0.45520473
H	5.34008973	5.38478969	-1.16137526
C	5.01059058	7.51664338	-1.06245760
H	4.55449042	7.58351791	-2.05759789
C	4.34979984	8.53118042	-0.15000392
H	6.06411202	7.78893741	-1.16267365
C	2.27672890	8.50183246	3.63883741
H	1.22788043	8.73963292	3.82798860
C	2.29703349	9.31465779	1.18089941
H	1.24669150	9.03463965	1.32549999
C	2.98259483	9.31269341	2.58511600
H	4.01847223	8.97691514	2.49336214
H	3.05400764	10.35085266	2.93146573
O	4.96366115	9.49487296	0.26209876
C	2.85562771	7.54325240	4.36355372
O	2.27762383	6.78354906	5.33909740
H	3.89860819	7.26692144	4.23652764
C	2.24825917	10.75898213	0.58934249
C	1.23527793	11.61412433	1.36950329
H	1.26396594	12.65528840	1.03160714
H	0.21322023	11.24525040	1.21508160
H	1.42625579	11.61492476	2.44664652
C	-1.94936833	-3.14895029	-0.28266181
H	-2.91108830	-3.37413193	-0.74185417
H	-1.16711293	-3.76666938	-0.73326366
H	-1.97768986	-3.38846849	0.78406779
C	1.89592922	10.81544747	-0.90450677
H	2.67174997	10.37204816	-1.53444907
H	0.94858402	10.30305361	-1.11689326
H	1.77897561	11.85622905	-1.22459733
H	3.24289938	11.19880207	0.71054765
H	1.35990906	7.06380722	5.44498310

Tautomer E = -1552.0680 (Fig. 3S)

0 1

C	0.00000000	0.00000000	0.00000000
C	1.54632788	0.00000000	0.00000000
C	1.98592143	1.50681494	0.00000000
C	1.39207670	2.43116199	1.11903931
C	-0.15319918	2.27596328	1.07656402
C	-0.62839495	0.81906847	1.11921726
H	-0.35171809	0.39202526	-0.95975170
H	-0.61319909	2.81455028	1.91067300
H	-0.53569865	2.74143079	0.15803607
H	-0.40156735	0.35456475	2.08483154
H	-1.71611009	0.78748788	1.00398705
H	1.54449972	1.90082548	-0.93048473
C	1.86181403	2.08473454	2.55537673
H	2.87265451	1.67680477	2.59769289
H	1.83774361	2.96721906	3.19865416
H	1.20713885	1.34464705	3.01999415
C	1.73879799	3.91760146	0.70082960
H	1.22372339	4.01247601	-0.26259315
C	3.49867512	1.72867902	-0.14214025
H	3.91215427	1.05965966	-0.90203072
H	4.01964484	1.48074795	0.78831449
C	3.80208591	3.17191369	-0.56776868
H	3.37135049	3.31030568	-1.56461214
H	4.88549325	3.29284008	-0.67862986
C	3.24839071	4.24771154	0.40149737
C	2.00278812	-0.66599050	-1.31860147
H	3.08053180	-0.84730598	-1.33344270
H	1.50936596	-1.63469734	-1.43535999
H	1.74984329	-0.05033459	-2.18907274
C	2.11849008	-0.83487348	1.16353689
H	1.73446496	-0.53591368	2.13885100
H	1.86841695	-1.88938787	1.02244800
H	3.20910623	-0.75402055	1.19553475
O	-0.45481682	-1.37669759	0.09187742
C	-1.62750807	-1.67962655	-0.50432977
O	-2.31198967	-0.88495464	-1.10192721
C	4.11808938	4.20358752	1.68363363
H	5.16582980	4.40740706	1.45010310
H	3.80911093	4.90886560	2.45704085
H	4.09954612	3.21502723	2.13776558
C	3.33429260	5.70743449	-0.27884122
C	1.11528806	4.99126064	1.60707942
H	0.05630057	4.77817717	1.77681140
H	1.58679194	4.99585531	2.59621444
C	2.68947659	6.76678786	0.68732010
C	1.23299258	6.39627801	1.00348360
H	0.81009300	7.10773755	1.71479969
H	0.62544575	6.46412029	0.09073730
C	2.62901128	5.71786119	-1.66279663
H	2.42634841	6.73359275	-2.00830440
H	3.25635885	5.25010508	-2.42641009
H	1.67099812	5.19851825	-1.66721267
H	3.24608732	6.70553848	1.62841802
C	2.83827662	8.24808030	0.18516251
H	2.29663008	8.30160108	-0.76707351
C	4.81110350	6.11474966	-0.51887921
H	5.36845801	6.08759582	0.42162190
H	5.30056722	5.40512486	-1.19309980
C	4.95699178	7.53572553	-1.09302670
H	4.48763415	7.60091718	-2.08166271
C	4.30020228	8.53315251	-0.16163229
H	6.00832581	7.81272168	-1.20259237

C	2.07401550	8.66056072	3.60840378
H	1.07028927	9.03568021	3.79025654
C	2.22014012	9.32862743	1.11587751
H	1.16798134	9.04759348	1.24290972
C	2.87573089	9.34971881	2.53695688
H	3.88839030	8.94064647	2.46787590
H	3.03081548	10.39375938	2.83267257
O	4.92299468	9.47015257	0.29742564
C	2.46763417	7.63630186	4.36871351
O	3.67409799	6.99320180	4.34028650
H	1.82268347	7.19105464	5.11915385
C	2.18933898	10.76073493	0.49730410
C	1.18634414	11.64212285	1.26056570
H	1.22664245	12.67613852	0.90277133
H	0.16051372	11.28105820	1.11383442
H	1.37783464	11.66189148	2.33753144
C	-1.95462435	-3.14220187	-0.32038967
H	-2.91137775	-3.36148587	-0.79266261
H	-1.16940446	-3.76136441	-0.76374454
H	-1.99747513	-3.38586071	0.74489167
C	1.83879770	10.79203725	-0.99814111
H	2.61673451	10.34365735	-1.62210944
H	0.89420339	10.27213902	-1.20340851
H	1.71768021	11.82725167	-1.33367945
H	3.18941418	11.19039348	0.61225341
H	4.26608136	7.46749191	3.74332927

Protonated compounds calculated as cations (Fig. 4S):

H^+ at C=O, E = -1552.4322

1 1

C	0.00000000	0.00000000	0.00000000
C	1.54720649	0.00000000	0.00000000
C	1.98377350	1.50722698	0.00000000
C	1.38913102	2.42931875	1.12111756
C	-0.15647352	2.27224026	1.08480863
C	-0.62583875	0.81401746	1.12582413
H	-0.35095345	0.40354907	-0.95589768
H	-0.61386822	2.80680726	1.92354951
H	-0.54538984	2.73724041	0.16833514
H	-0.39500580	0.34806164	2.08941729
H	-1.71320600	0.78188349	1.01430165
H	1.53972302	1.89972985	-0.93006175
C	1.86621141	2.09049087	2.55617575
H	2.87843364	1.68564816	2.59833210
H	1.83609107	2.97094306	3.20282316
H	1.21655811	1.34696265	3.01989908
C	1.72928264	3.91477672	0.69370593
H	1.21815792	3.99704230	-0.27230373
C	3.49622822	1.72989577	-0.14416566
H	3.90715535	1.06503277	-0.90788758
H	4.02126811	1.47824643	0.78241691
C	3.80325661	3.17327410	-0.56868506
H	3.37738441	3.31182348	-1.56754128
H	4.88816296	3.29501687	-0.67108897
C	3.24011134	4.24239000	0.40187259
C	2.00518735	-0.66215205	-1.31980874
H	3.08169776	-0.85116806	-1.33268735
H	1.50722761	-1.62742476	-1.43937198
H	1.75609670	-0.04479065	-2.19029937
C	2.11952001	-0.83569503	1.16268304
H	1.73695669	-0.53952488	2.13952606

H	1.86443626	-1.88817055	1.01959068
H	3.21078726	-0.76234102	1.19430944
O	-0.45426514	-1.37117809	0.07914463
C	-1.64708209	-1.65174225	-0.50131031
O	-2.32876552	-0.82880880	-1.06110934
C	4.10331122	4.21255762	1.68727870
H	5.14947283	4.44578410	1.47342766
H	3.76051816	4.89105293	2.47052829
H	4.10702426	3.21614505	2.12185471
C	3.31717955	5.70618112	-0.28602638
C	1.09439384	4.98921258	1.59025840
H	0.03103034	4.78224353	1.73156971
H	1.53991570	4.99870830	2.58936018
C	2.68059530	6.74003889	0.69868346
C	1.21455860	6.39936071	0.99058793
H	0.78458781	7.10613923	1.70227526
H	0.62160461	6.47645559	0.07031515
C	2.60237735	5.71760084	-1.66442611
H	2.36709612	6.72916621	-2.00976247
H	3.23209199	5.26661148	-2.43493756
H	1.65821275	5.17686945	-1.66466393
H	3.22325430	6.69127161	1.64508877
C	2.82792004	8.26696354	0.18816279
H	2.29220268	8.26791147	-0.76648697
C	4.78655822	6.11298936	-0.52632916
H	5.36390576	6.09266149	0.40015437
H	5.28308874	5.44160544	-1.22999977
C	4.90699267	7.55661632	-1.12148227
H	4.38724687	7.59595464	-2.08297748
C	4.24581217	8.45129221	-0.16197466
H	5.94941826	7.84808221	-1.25803341
C	2.01400283	9.52972873	3.71663050
H	2.43725403	10.15774668	4.51346996
C	2.20342200	9.36284133	1.10228681
H	1.18572593	9.01533502	1.29868651
C	2.92842549	9.51360123	2.47975162
H	3.63227272	8.69070168	2.64348189
H	3.50484643	10.45183991	2.49135106
O	5.02235605	9.29354076	0.42564576
C	1.80306670	8.16337229	4.33383987
O	2.35672864	7.15644905	3.96023736
H	1.10208217	8.14106205	5.19245913
C	2.04618382	10.73486818	0.36808976
C	1.28533366	11.74868670	1.23822525
H	1.21224992	12.70764879	0.71856259
H	0.26345171	11.40465773	1.43537031
H	1.77091426	11.93896025	2.19859022
C	-1.98532863	-3.11251373	-0.34737195
H	-2.95219524	-3.31061831	-0.80777765
H	-1.21441559	-3.72873688	-0.81884067
H	-2.01262392	-3.38139646	0.71232938
C	1.34288802	10.61473224	-0.99438352
H	1.93693910	10.08617990	-1.74558417
H	0.37906551	10.10130775	-0.89904757
H	1.14248494	11.60985021	-1.39995521
H	3.04977272	11.15296430	0.18651479
H	1.03101620	9.96898389	3.50888959
H	4.56009721	9.80971273	1.11511864

H^+ at CH(=O), E = -1552.4069

1 1

C	0.00000000	0.00000000	0.00000000
C	1.54712224	0.00000000	0.00000000

C	1.98496970	1.50694229	0.00000000
C	1.39031871	2.42885137	1.12100083
C	-0.15525494	2.27201292	1.08531132
C	-0.62593418	0.81413268	1.12590202
H	-0.35108987	0.40394454	-0.95565468
H	-0.61368745	2.80613377	1.92434192
H	-0.54364677	2.73762347	0.16907239
H	-0.39593963	0.34743208	2.08949443
H	-1.71318468	0.78273200	1.01359588
H	1.54137918	1.90038703	-0.92961605
C	1.86811683	2.08738005	2.55532647
H	2.88256674	1.68864393	2.59680456
H	1.83354497	2.96575083	3.20579654
H	1.22193594	1.33980126	3.01744796
C	1.73478830	3.91254195	0.69406040
H	1.22644048	3.99846831	-0.27247173
C	3.49721949	1.73036672	-0.14285638
H	3.90737844	1.06755547	-0.90871246
H	4.02155905	1.47197111	0.78263495
C	3.80797940	3.17453499	-0.56218435
H	3.38721275	3.31791272	-1.56203320
H	4.89240136	3.29421896	-0.66120828
C	3.24466404	4.24907118	0.40414382
C	2.00521371	-0.66194028	-1.31984024
H	3.08183548	-0.84977821	-1.33272503
H	1.50785115	-1.62754144	-1.43941156
H	1.75583692	-0.04430274	-2.18991541
C	2.11895662	-0.83646683	1.16251959
H	1.73390116	-0.54295537	2.13934315
H	1.86622119	-1.88928660	1.01775022
H	3.20996817	-0.76099493	1.19599533
O	-0.45421800	-1.37122969	0.07909454
C	-1.65131372	-1.64983605	-0.49347167
O	-2.33931107	-0.82386530	-1.04089775
C	4.10846798	4.20507249	1.68879395
H	5.14457027	4.47974884	1.48211238
H	3.74845554	4.85422370	2.49182134
H	4.13822402	3.20065750	2.10402691
C	3.33276694	5.70694697	-0.27900716
C	1.09035683	4.98775032	1.58397449
H	0.02750815	4.77714258	1.72288139
H	1.52833854	4.97546623	2.59064226
C	2.68370578	6.77537538	0.68032838
C	1.22231734	6.39253299	0.97947473
H	0.73915497	7.11407542	1.64923635
H	0.64953887	6.45052785	0.04675033
C	2.61517402	5.72143723	-1.65812074
H	2.39319568	6.73444195	-1.99984626
H	3.25023517	5.27084507	-2.42414005
H	1.66968870	5.18134237	-1.66554001
H	3.27776702	6.69344515	1.61665380
C	2.84283918	8.25387917	0.18142318
H	2.23697982	8.31887658	-0.72784463
C	4.80538533	6.12093626	-0.52890140
H	5.36884449	6.12411652	0.40904177
H	5.29507513	5.39426227	-1.18193795
C	4.93854285	7.52143323	-1.15342456
H	4.46568028	7.54953943	-2.14187959
C	4.28468737	8.55073128	-0.26602014
H	5.98584628	7.80300208	-1.28183412
C	2.56642592	9.68366553	3.73822443
H	3.22257780	10.12976810	4.51253962
C	2.31317532	9.34589767	1.15963754

H	1.34066685	8.99451738	1.53174041
C	3.26580191	9.52974990	2.38416805
H	3.98833135	8.70784577	2.45582302
H	3.89253952	10.40768035	2.22992438
O	4.87216618	9.54915628	0.09719896
C	2.05470560	8.48502755	4.38581803
O	2.13933111	7.30399629	3.94509409
H	1.55941305	8.53815545	5.35826197
C	2.01522682	10.70516489	0.45231328
C	1.48900127	11.76731439	1.43298628
H	1.27549123	12.69785728	0.90013899
H	0.54889132	11.45057186	1.90519843
H	2.20985223	12.02300469	2.21664475
C	-1.98457294	-3.11279826	-0.34980817
H	-2.96378579	-3.30542776	-0.78581152
H	-1.22742485	-3.72082090	-0.85340132
H	-1.98029579	-3.39782033	0.70589275
C	1.00843689	10.56015034	-0.70061448
H	1.39092710	9.96744459	-1.53498898
H	0.07043986	10.10371342	-0.36132867
H	0.76220579	11.54569307	-1.10558958
H	2.96277777	11.06931200	0.04344344
H	1.72396181	10.39736078	3.72363183
H	2.56297954	7.23018096	3.05078579

tautomer 1, H^+ at CH(=O), E = -1552.3905

1 1

C	0.00000000	0.00000000	0.00000000
C	1.54683433	0.00000000	0.00000000
C	1.98874281	1.50489163	0.00000000
C	1.39372564	2.43194700	1.11816282
C	-0.15185582	2.27771654	1.07467179
C	-0.62586076	0.82076807	1.12054972
H	-0.35262342	0.39555049	-0.95865281
H	-0.61351000	2.81768349	1.90796498
H	-0.53306233	2.74087275	0.15418284
H	-0.39582671	0.35812932	2.08617602
H	-1.71321297	0.78962315	1.00825218
H	1.55072830	1.89966461	-0.93149386
C	1.86438892	2.08628709	2.55403438
H	2.87413458	1.67615737	2.59592117
H	1.83982606	2.96695505	3.20178823
H	1.20990708	1.34615972	3.01748216
C	1.74666818	3.91425578	0.69341605
H	1.24801628	4.00266134	-0.27830401
C	3.50289020	1.72032904	-0.14104758
H	3.91060613	1.05535830	-0.90661178
H	4.02596907	1.45990206	0.78525548
C	3.81548693	3.16315915	-0.55926856
H	3.38970344	3.31172289	-1.55662774
H	4.90003458	3.27933683	-0.66504660
C	3.26085215	4.23668972	0.41178936
C	2.00342926	-0.66606660	-1.31812405
H	3.08027183	-0.85227290	-1.33296107
H	1.50706525	-1.63280778	-1.43325697
H	1.75117535	-0.05222522	-2.18998778
C	2.11634581	-0.83580493	1.16414901
H	1.71936630	-0.54948773	2.13859143
H	1.87490709	-1.89052836	1.01379465
H	3.20627783	-0.74966189	1.20750182
O	-0.45071845	-1.37301331	0.09115665
C	-1.64607455	-1.66388377	-0.47576609
O	-2.34114479	-0.84946745	-1.03171588

C	4.12612307	4.18253818	1.69507900
H	5.16684363	4.42985831	1.47509021
H	3.78501049	4.85918761	2.48281945
H	4.13288875	3.18353646	2.12398610
C	3.36143327	5.69229228	-0.25785602
C	1.10299598	5.00139550	1.57201589
H	0.03983243	4.79195362	1.71345516
H	1.54634933	5.00638347	2.57661779
C	2.71882877	6.75489758	0.70001459
C	1.24167202	6.40206195	0.95759518
H	0.77342148	7.12117294	1.63827025
H	0.66923419	6.46146147	0.02548375
C	2.66201124	5.73045132	-1.64466479
H	2.44962949	6.75870126	-1.94633247
H	3.29917142	5.29264208	-2.41750628
H	1.71358499	5.19534023	-1.67142374
H	3.21855558	6.63542311	1.67696709
C	2.99223677	8.20638142	0.28549066
C	4.83108667	6.11979657	-0.47942278
H	5.35062595	6.19737185	0.47977754
H	5.36856942	5.37627926	-1.07368669
C	4.92800774	7.48263946	-1.17321293
H	4.70128582	7.40311285	-2.24512585
C	4.02052118	8.49621316	-0.54354583
H	5.96146053	7.85174917	-1.11074079
C	2.62172892	10.45078480	3.28353616
H	3.25836845	11.19373791	3.81361976
C	2.23378827	9.33731148	0.97282287
H	1.49360236	8.87455757	1.63508463
C	3.20917540	10.15417401	1.89737104
H	4.14657329	9.61012032	2.02674382
H	3.47126311	11.10538970	1.43083706
O	4.30887532	9.80686195	-0.81464338
C	2.55585247	9.35951945	4.24015311
O	3.24276582	8.29153391	4.05385769
H	1.95688225	9.43299707	5.15016852
C	1.42307408	10.26313980	0.01274626
C	0.66302251	11.36581846	0.76453298
H	0.08791680	11.97489647	0.06102386
H	-0.05629625	10.94334403	1.47911274
H	1.32462367	12.05542224	1.30023281
C	-1.96914701	-3.12803929	-0.31590959
H	-2.95243785	-3.33044017	-0.73813032
H	-1.21470104	-3.73507761	-0.82492096
H	-1.94905489	-3.40521703	0.74166366
C	0.43643220	9.45807719	-0.84367073
H	0.94322092	8.68903774	-1.43100984
H	-0.32237651	8.96619240	-0.22360487
H	-0.08873564	10.11822774	-1.54033572
H	2.14165913	10.74441007	-0.65685823
H	1.63743978	10.93870356	3.26959887
H	5.04074396	9.85175831	-1.44083807
H	3.15868856	7.64198319	4.77466353

tautomer 2, H^+ at CH(=O), E = -1552.3864

1 1

C	0.00000000	0.00000000	0.00000000
C	1.54677895	0.00000000	0.00000000
C	1.99018105	1.50473672	0.00000000
C	1.38909759	2.43672037	1.10980561
C	-0.15629414	2.27588296	1.07188166
C	-0.62588801	0.81800239	1.12250864
H	-0.35218860	0.39902761	-0.95724734

H	-0.61779349	2.81909067	1.90320543
H	-0.54186110	2.73325305	0.15052194
H	-0.39317850	0.35627132	2.08795766
H	-1.71340951	0.78379485	1.01227307
H	1.56099624	1.89755035	-0.93640310
C	1.86389556	2.10118239	2.54613567
H	2.89269300	1.74118326	2.59471454
H	1.78791321	2.97323418	3.20153900
H	1.24242414	1.32591745	2.99669211
C	1.72976643	3.91904244	0.67498204
H	1.20160604	4.01260195	-0.28026584
C	3.50633079	1.71937371	-0.12574829
H	3.92742608	1.03830313	-0.86975158
H	4.01779234	1.48281788	0.81338840
C	3.81556617	3.15306456	-0.57525889
H	3.41128937	3.26401788	-1.58533920
H	4.90052283	3.28281101	-0.66233077
C	3.23229516	4.25128598	0.35058123
C	2.00368007	-0.66521142	-1.31853182
H	3.08007127	-0.85425677	-1.33191373
H	1.50510131	-1.63049023	-1.43621221
H	1.75469229	-0.04921173	-2.18970423
C	2.11647474	-0.83675600	1.16332409
H	1.72460226	-0.54709554	2.13882930
H	1.86947130	-1.89068120	1.01574662
H	3.20691583	-0.75572148	1.20267722
O	-0.45148799	-1.37311282	0.08558052
C	-1.64472377	-1.66082522	-0.48785097
O	-2.33615472	-0.84382817	-1.04445085
C	4.09957284	4.25886012	1.63593444
H	5.13892710	4.51385002	1.41699361
H	3.74478402	4.95445811	2.40027874
H	4.11842195	3.27273566	2.09570985
C	3.28818098	5.68260655	-0.38072800
C	1.12770143	5.00069093	1.59141759
H	0.06824023	4.79987943	1.77057278
H	1.60511688	4.98294875	2.57891318
C	2.69445905	6.76808340	0.57764796
C	1.25602842	6.40569840	0.99149471
H	0.86480374	7.13574203	1.71032012
H	0.60109734	6.48510266	0.11569077
C	2.51106798	5.69124669	-1.72398511
H	2.59012354	6.67137336	-2.19936535
H	2.92963297	4.97467465	-2.43258484
H	1.44953175	5.46616293	-1.62185698
H	3.31828085	6.75656595	1.48070817
C	2.75736843	8.22124633	0.02348080
H	1.91171728	8.35708363	-0.66078510
C	4.74975740	6.07746098	-0.72377339
H	5.42747866	5.86131004	0.10975584
H	5.10891635	5.45810721	-1.55328210
C	4.89651876	7.52403074	-1.10487910
C	4.00436057	8.46669478	-0.78871577
H	5.76773366	7.80035569	-1.69771434
C	3.92836958	9.98857897	3.28443046
H	4.86254492	10.49085847	3.61959719
C	2.58079741	9.28994031	1.16871927
H	1.97194187	8.81179865	1.94811664
C	3.96322450	9.62181398	1.79984644
H	4.64192803	8.77292631	1.66759960
H	4.42353538	10.45295471	1.26241716
O	4.11640690	9.77626419	-1.18737587
C	3.76476654	8.94259529	4.27217491

O	3.75514868	7.71191678	3.93937821
H	3.66322044	9.18868342	5.33291463
C	1.77829879	10.53732230	0.68403299
C	1.83265080	11.74663895	1.63165470
H	1.26207878	12.57776266	1.20694555
H	1.37176630	11.52487990	2.60466176
H	2.84876814	12.12216438	1.79320910
C	-1.97080416	-3.12500526	-0.33451622
H	-2.95240966	-3.32411210	-0.76222739
H	-1.21506007	-3.73160770	-0.84209130
H	-1.95642596	-3.40587907	0.72216481
C	0.30262990	10.18122680	0.43833551
H	0.17427549	9.35584919	-0.26567308
H	-0.19411806	9.89905712	1.37487608
H	-0.23381576	11.04228402	0.02938971
H	2.22248036	10.85087957	-0.26550894
H	3.17772871	10.75675411	3.54031836
H	4.87189419	9.86223601	-1.78273188
H	3.65683302	7.09376290	4.68761667

tautomer 3, H^+ at C=O, E = -1552.4138

1 1

C	0.00000000	0.00000000	0.00000000
C	1.54731592	0.00000000	0.00000000
C	1.98823543	1.50589220	0.00000000
C	1.39535544	2.42930670	1.12113901
C	-0.15040599	2.27999331	1.07170381
C	-0.62674765	0.82419476	1.11762986
H	-0.35136938	0.39392765	-0.95986916
H	-0.61391295	2.82231493	1.90214403
H	-0.52763895	2.74125944	0.14840452
H	-0.40356736	0.36257625	2.08519326
H	-1.71374253	0.79689712	1.00131994
H	1.54599837	1.89962762	-0.93044767
C	1.86057504	2.07787855	2.55742071
H	2.86706957	1.65966104	2.60213834
H	1.83803420	2.95347998	3.21088820
H	1.20007997	1.33923035	3.01358929
C	1.74677271	3.91615579	0.70545309
H	1.23858370	4.01151035	-0.26100184
C	3.50142178	1.72238742	-0.14610586
H	3.90698875	1.05876461	-0.91369835
H	4.02968211	1.46489013	0.77757849
C	3.80981396	3.16603707	-0.56698558
H	3.37299017	3.31249928	-1.56010315
H	4.89413729	3.28504833	-0.68091159
C	3.26024369	4.23160083	0.41541687
C	2.00412513	-0.66274662	-1.32011602
H	3.08035708	-0.85383601	-1.33428955
H	1.50485253	-1.62741482	-1.43915598
H	1.75503604	-0.04593027	-2.19105527
C	2.11926101	-0.83766994	1.16144756
H	1.73279046	-0.54843728	2.13922476
H	1.86840599	-1.89030486	1.01310387
H	3.21027469	-0.76084781	1.19609443
O	-0.45055412	-1.37108755	0.09303873
C	-1.64363231	-1.66210341	-0.48232080
O	-2.33015032	-0.84682127	-1.04703324
C	4.12761112	4.17442148	1.69682129
H	5.18162802	4.36385811	1.47778822
H	3.81580038	4.86839218	2.47981297
H	4.09222534	3.18185123	2.13750307
C	3.35493785	5.70319403	-0.25563462

C	1.11835921	4.99185492	1.60633891
H	0.05291099	4.79288871	1.74380158
H	1.56022385	4.98628809	2.60852727
C	2.72562892	6.72936886	0.73801439
C	1.25605106	6.40437239	1.01656565
H	0.83556641	7.11494426	1.72914568
H	0.67019053	6.49362060	0.09319152
C	2.64478590	5.73931753	-1.63561716
H	2.42465792	6.75899923	-1.96863197
H	3.26810131	5.28571062	-2.41025188
H	1.69194197	5.21345604	-1.64224246
H	3.26470247	6.66096310	1.68528099
C	2.89342481	8.26767737	0.27241712
H	2.35341763	8.30418703	-0.68347187
C	4.82910225	6.09525806	-0.48328533
H	5.40064314	6.05659897	0.44653991
H	5.32235220	5.42789823	-1.19323363
C	4.95739156	7.54388629	-1.05807783
H	4.44296206	7.59699798	-2.02266468
C	4.29888282	8.45266930	-0.10352960
H	6.00653739	7.81860066	-1.21374382
C	2.12577748	8.44309214	3.65150639
H	1.06952150	8.66641635	3.80330689
C	2.28734471	9.34068658	1.22155083
H	1.22822652	9.07300047	1.28709805
C	2.87495657	9.30420697	2.67079220
H	3.92381910	8.99169474	2.65432782
H	2.89382782	10.33529560	3.04184128
O	4.98562299	9.36658281	0.49414180
C	2.69813134	7.47317737	4.37044259
O	2.11397161	6.66778988	5.28945302
H	3.75407797	7.23121564	4.27574215
C	2.30805064	10.79017970	0.63348504
C	1.23646390	11.64646129	1.32956299
H	1.31033565	12.68900540	1.00785600
H	0.23141283	11.29093150	1.07464295
H	1.32804711	11.63328909	2.41873802
C	-1.97203776	-3.12394954	-0.31836235
H	-2.95526204	-3.32407994	-0.74173623
H	-1.21910878	-3.73521513	-0.82464598
H	-1.95444637	-3.39798135	0.74000149
C	2.09040273	10.86078084	-0.88597607
H	2.90569133	10.41155630	-1.46529441
H	1.15638119	10.36796238	-1.18272927
H	2.01998865	11.90376633	-1.20665101
H	3.28679884	11.23393486	0.85492395
H	1.19287739	6.93009008	5.41676815
H	5.91070822	9.39541858	0.18934044

tautomer 4, H^+ at C=O, E = -1552.4120

1 1

C	0.00000000	0.00000000	0.00000000
C	1.54722523	0.00000000	0.00000000
C	1.98566884	1.50683402	0.00000000
C	1.38929273	2.42946433	1.12020154
C	-0.15632422	2.27389389	1.08083335
C	-0.62658089	0.81613036	1.12364645
H	-0.35090983	0.40123456	-0.95680621
H	-0.61568568	2.81099337	1.91685597
H	-0.54237066	2.73711360	0.16223376
H	-0.39820954	0.35137493	2.08837445
H	-1.71381791	0.78485748	1.01029588
H	1.54487662	1.89952977	-0.93147672

C	1.86387434	2.09031145	2.55541851
H	2.87978786	1.69559609	2.59871037
H	1.82296379	2.96827519	3.20500467
H	1.22093319	1.33881485	3.01558152
C	1.73075097	3.91530552	0.69404992
H	1.21906096	3.99932788	-0.27122897
C	3.49886933	1.72805784	-0.13963944
H	3.91156231	1.06150467	-0.90071366
H	4.02219059	1.47869833	0.78873916
C	3.80738167	3.17078789	-0.56647888
H	3.38447656	3.30843307	-1.56673740
H	4.89312768	3.29184137	-0.66565379
C	3.24186170	4.24088956	0.40148896
C	2.00339725	-0.66283522	-1.31993971
H	3.08124756	-0.84413716	-1.33941655
H	1.51176422	-1.63203299	-1.43360241
H	1.74324327	-0.04962679	-2.19013833
C	2.11885662	-0.83523381	1.16366268
H	1.73588501	-0.53743625	2.13976647
H	1.86263071	-1.88763049	1.02216009
H	3.21033256	-0.76320693	1.19534178
O	-0.45309130	-1.37095108	0.08343934
C	-1.64433258	-1.65545150	-0.49941317
O	-2.32248629	-0.83661790	-1.06914501
C	4.10544463	4.21473006	1.68721124
H	5.15337458	4.43835774	1.46924763
H	3.76865429	4.89786819	2.46962053
H	4.10321434	3.22130878	2.12854961
C	3.31620877	5.70465263	-0.28993459
C	1.09858202	4.99196793	1.59013495
H	0.03670707	4.78424395	1.74016364
H	1.55124663	4.99990530	2.58717645
C	2.67939597	6.73761313	0.69272600
C	1.21310033	6.40101870	0.98587327
H	0.79107668	7.11253979	1.69647627
H	0.62254349	6.47170068	0.06391897
C	2.60330914	5.71549048	-1.66936701
H	2.34787181	6.72623678	-2.00343217
H	3.24091315	5.28146552	-2.44342632
H	1.66945026	5.15764719	-1.67523848
H	3.22600435	6.68204184	1.63742811
C	2.83151287	8.27194501	0.19906555
H	2.29284901	8.28680164	-0.75627987
C	4.78570354	6.11041799	-0.53099224
H	5.36349114	6.08855302	0.39562213
H	5.28331759	5.44137852	-1.23624620
C	4.89712049	7.55590047	-1.12445098
H	4.37220983	7.59513278	-2.08295336
C	4.24102521	8.45205219	-0.15911249
H	5.94350337	7.83505253	-1.28883734
C	1.82561593	8.70651555	3.59865020
H	0.85968494	9.19259971	3.69855483
C	2.21806133	9.34801530	1.13583466
H	1.15772879	9.08327792	1.18377977
C	2.78270899	9.29667136	2.59800187
H	3.73266921	8.75034823	2.61135444
H	3.04672944	10.31636017	2.89979026
O	4.93488682	9.32877410	0.48674641
C	2.03965725	7.64159878	4.37562156
O	3.16800624	6.87546355	4.44985869
H	1.27860500	7.26132208	5.04868480
C	2.25627965	10.79446986	0.55012978
C	1.24545539	11.68024240	1.29738654

H	1.32929899	12.71949748	0.96792398
H	0.21927573	11.35053784	1.09847500
H	1.39572829	11.66823862	2.38028739
C	-1.98465852	-3.11434030	-0.33374476
H	-2.95103533	-3.31509352	-0.79400371
H	-1.21391378	-3.73549642	-0.79901686
H	-2.01379592	-3.37435038	0.72810609
C	1.97132861	10.86655349	-0.95808632
H	2.75634343	10.41062888	-1.57282942
H	1.02072436	10.38192736	-1.21228845
H	1.89661925	11.91001939	-1.27582820
H	3.25679455	11.20965374	0.72520530
H	3.91206343	7.35378429	4.06636889
H	5.86482245	9.35355463	0.19683132

Structures with hydrogen bonds and similar weak interactions

IV (Fig. 5S) E = -1552.4370

1 1

C	0.00000000	0.00000000	0.00000000
C	1.54689266	0.00000000	0.00000000
C	1.98581181	1.50654978	0.00000000
C	1.39154383	2.43005904	1.12089667
C	-0.15422359	2.27322435	1.08348265
C	-0.62503961	0.81506884	1.12518348
H	-0.35052811	0.40336609	-0.95620898
H	-0.61354353	2.80892048	1.92083808
H	-0.54190237	2.73701091	0.16590631
H	-0.39504000	0.34907525	2.08904451
H	-1.71238780	0.78406089	1.01355301
H	1.54424056	1.90062806	-0.93030333
C	1.86870323	2.08829399	2.55495843
H	2.88321905	1.68998805	2.59664148
H	1.83272519	2.96530209	3.20672551
H	1.22273015	1.33983868	3.01562400
C	1.73381039	3.91511033	0.69468843
H	1.22187991	4.00096871	-0.26962892
C	3.49926971	1.72446974	-0.14209768
H	3.90829438	1.05967149	-0.90664915
H	4.02387764	1.46727620	0.78357489
C	3.81103141	3.16637210	-0.56560592
H	3.39258628	3.30411652	-1.56735578
H	4.89660387	3.28733241	-0.66157106
C	3.24605263	4.24107632	0.39921670
C	2.00338123	-0.66215390	-1.32029905
H	3.08085224	-0.84574860	-1.33809164
H	1.51012006	-1.63014790	-1.43616846
H	1.74698320	-0.04765279	-2.19066218
C	2.12027560	-0.83649343	1.16152915
H	1.73459367	-0.54713213	2.13941191
H	1.87079511	-1.88974963	1.01431325
H	3.21106434	-0.75778138	1.19532875
O	-0.45231610	-1.37109980	0.08013698
C	-1.64523853	-1.65410423	-0.49991776
O	-2.33053314	-0.83092136	-1.05460038
C	4.10965029	4.20401828	1.68406970
H	5.14431615	4.49206974	1.48539697
H	3.73785869	4.83874935	2.49101078
H	4.15430629	3.19456422	2.08538235
C	3.32314332	5.70225062	-0.28938200
C	1.09705956	4.99153423	1.59174491
H	0.03385527	4.78440599	1.73231224
H	1.54140454	4.98391333	2.59371049

C	2.69568680	6.74138312	0.71417251
C	1.22815956	6.40451752	0.99991113
H	0.78626115	7.12290341	1.69638638
H	0.64190852	6.48451568	0.07694047
C	2.58212769	5.71678735	-1.65441904
H	2.40495513	6.72784822	-2.03019877
H	3.17357209	5.20933285	-2.41902472
H	1.60827676	5.23191527	-1.62665470
H	3.25104279	6.65997757	1.65351398
C	2.88777365	8.24088741	0.18400379
H	2.49617378	8.18662181	-0.82941048
C	4.79466543	6.09790888	-0.57597885
H	5.41658025	6.00529576	0.31622442
H	5.23360238	5.45475977	-1.34178636
C	4.96433323	7.58254846	-1.08780555
H	4.43893648	7.72300760	-2.03241293
C	4.35734662	8.34835389	0.01406222
H	6.02387727	7.81623591	-1.19806862
C	2.37868523	8.95376387	3.44570905
H	1.96264055	9.45704325	4.33075437
C	2.10725801	9.43822540	0.81634189
H	1.06188455	9.10579491	0.81913267
C	2.42589069	9.92447900	2.25305355
H	3.38270649	10.46083007	2.27162871
H	1.68039136	10.68932033	2.46864638
O	5.16839921	8.83987523	0.85835990
C	3.69364373	8.40729952	3.88948668
O	4.74280190	8.53586773	3.27800144
H	3.71164282	7.86523710	4.85013607
C	2.16969546	10.66876449	-0.16336002
C	1.32406052	11.85736021	0.33135836
H	1.29905294	12.63706829	-0.43412346
H	0.28747286	11.55395926	0.52137393
H	1.71705163	12.32059409	1.23837049
C	-1.97525699	-3.11746003	-0.35449840
H	-2.94961862	-3.31493194	-0.79901967
H	-1.21111241	-3.72448248	-0.84869044
H	-1.97998627	-3.39886181	0.70215765
C	1.72607367	10.32911889	-1.59985049
H	2.41816598	9.67284752	-2.13494179
H	0.73449102	9.86139208	-1.61211363
H	1.65848589	11.24552060	-2.19139181
H	3.21787278	11.00318485	-0.20367215
H	1.70310977	8.10020859	3.29692000
H	4.83243211	8.95763021	1.82708838

V (Fig. 5S) E = -1552.4458

1 1

C	0.00000000	0.00000000	0.00000000
C	1.54645791	0.00000000	0.00000000
C	1.98572726	1.50661995	0.00000000
C	1.39119044	2.43332591	1.11652447
C	-0.15502081	2.27685552	1.07492063
C	-0.62552460	0.81907472	1.12188796
H	-0.35164029	0.39933668	-0.95744553
H	-0.61750669	2.81648424	1.90767404
H	-0.53899615	2.73642104	0.15371004
H	-0.39650243	0.35653306	2.08764326
H	-1.71294567	0.78752969	1.00998568
H	1.54650464	1.89880200	-0.93229068
C	1.86324405	2.09106526	2.55203243
H	2.87685505	1.69032973	2.59537472
H	1.82590619	2.96788267	3.20316903

H	1.21521135	1.34304806	3.01090635
C	1.73566693	3.92013411	0.69403800
H	1.20641249	4.02168556	-0.26166204
C	3.49834922	1.72632051	-0.13968177
H	3.91345837	1.04963367	-0.89042244
H	4.02129209	1.49339581	0.79343625
C	3.79377628	3.16349118	-0.58830320
H	3.34931573	3.28272063	-1.58104411
H	4.87560719	3.28980684	-0.71373819
C	3.24307758	4.24720018	0.37439366
C	2.00264784	-0.66085760	-1.32127177
H	3.08054088	-0.84173556	-1.34124249
H	1.51078713	-1.62977749	-1.43697099
H	1.74330635	-0.04625081	-2.19067090
C	2.12180910	-0.83611291	1.16056782
H	1.73540618	-0.54788467	2.13843238
H	1.87549323	-1.89008643	1.01302405
H	3.21240768	-0.75349841	1.19369970
O	-0.45352514	-1.37132776	0.08463346
C	-1.64085192	-1.65828241	-0.50325413
O	-2.32207574	-0.84036510	-1.07100658
C	4.12895565	4.22688845	1.64584209
H	5.18346651	4.36528962	1.39397382
H	3.86201662	4.98545042	2.38529930
H	4.06339370	3.26749127	2.15282585
C	3.31896012	5.69587017	-0.32133438
C	1.12599958	4.99104831	1.61590215
H	0.07501020	4.76902546	1.81347809
H	1.62267171	5.00115961	2.59225793
C	2.65668441	6.75711291	0.62102082
C	1.21478441	6.38883965	0.99500505
H	0.80988126	7.12472172	1.69909276
H	0.57705946	6.43712615	0.10145042
C	2.64855233	5.69067505	-1.72069560
H	2.48920523	6.69811051	-2.12027410
H	3.26864629	5.16921881	-2.45323339
H	1.66966923	5.21171192	-1.72248715
H	3.24156134	6.74371933	1.54223266
C	2.71320529	8.20488564	0.05863853
H	1.85494441	8.31899597	-0.62118409
C	4.79290599	6.14347879	-0.50128055
H	5.28484155	6.19992600	0.47137134
H	5.35430650	5.41421793	-1.08985212
C	4.92281346	7.50706836	-1.19436325
H	4.93400863	7.42618638	-2.28937743
C	3.84152040	8.47119821	-0.87898772
H	5.87487125	8.00297477	-0.95612222
C	0.83187021	11.35453510	0.84147192
H	-0.22494611	11.62562414	0.96843999
C	2.54385565	9.41333450	1.09447997
H	3.11295018	10.25095062	0.67961838
C	1.05384653	9.86756391	1.14783970
H	0.45104624	9.29061274	0.44188844
H	0.62600156	9.65763217	2.13126014
O	3.87308023	9.56189257	-1.53278355
C	1.23424456	11.83339546	-0.52157593
O	1.95223522	11.25114574	-1.31918538
H	0.83550565	12.82159406	-0.81291086
C	3.18992897	9.17032836	2.48330688
C	2.95045719	10.35978122	3.43124189
H	3.44023198	10.17296917	4.39082478
H	3.37911835	11.28341251	3.02397747
H	1.89255618	10.53467375	3.64120598

C	-1.97273418	-3.12056072	-0.34929817
H	-2.94439697	-3.32064898	-0.79863412
H	-1.20587127	-3.73141810	-0.83451957
H	-1.98417802	-3.39460834	0.70922474
C	4.70381301	8.94185083	2.36196783
H	4.95947063	8.08484369	1.73248317
H	5.19717350	9.82867104	1.94593161
H	5.14658338	8.75619886	3.34432066
H	2.72807513	8.28708835	2.94101836
H	1.36305542	12.00748480	1.55307872
H	3.07923006	10.18141973	-1.36468195

VI (Fig. 5S) E = -1552.4394

1 1

C	0.00000000	0.00000000	0.00000000
C	1.54680701	0.00000000	0.00000000
C	1.98496023	1.50693096	0.00000000
C	1.39084640	2.43042236	1.12023535
C	-0.15515663	2.27408749	1.08154621
C	-0.62614925	0.81588444	1.12405755
H	-0.35064519	0.40215216	-0.95661805
H	-0.61503378	2.81084385	1.91777679
H	-0.54274395	2.73724618	0.16352070
H	-0.39679039	0.35065455	2.08823788
H	-1.71354295	0.78471447	1.01172936
H	1.54169125	1.89963171	-0.93015046
C	1.86552712	2.08681842	2.55460051
H	2.87992672	1.68735210	2.59677168
H	1.82865676	2.96290026	3.20735465
H	1.21885466	1.33789420	3.01365477
C	1.73306497	3.91618769	0.69581322
H	1.21213260	4.00564805	-0.26367989
C	3.49758604	1.72847942	-0.14424749
H	3.90824017	1.05926066	-0.90407851
H	4.02410664	1.48209288	0.78327831
C	3.79993587	3.16887843	-0.57882773
H	3.36845317	3.30028770	-1.57581578
H	4.88373307	3.29350872	-0.68907374
C	3.24156480	4.24463852	0.38783522
C	2.00364091	-0.66154564	-1.32072913
H	3.08178744	-0.84078755	-1.33966359
H	1.51382780	-1.63153942	-1.43520536
H	1.74339761	-0.04861232	-2.19106573
C	2.12137595	-0.83705000	1.16046536
H	1.73837071	-0.54728138	2.13922219
H	1.87072767	-1.89013371	1.01354028
H	3.21236743	-0.75914065	1.19145949
O	-0.45305424	-1.37112199	0.08193577
C	-1.63837382	-1.65844172	-0.51072353
O	-2.31723573	-0.84028506	-1.08062479
C	4.11691110	4.21611297	1.66488299
H	5.16654474	4.41576675	1.43536561
H	3.80373089	4.92269554	2.43662194
H	4.09472422	3.23118699	2.12444824
C	3.31140790	5.70251841	-0.30688046
C	1.10970594	4.99153605	1.60013459
H	0.05224845	4.77749888	1.77043795
H	1.57971245	4.99940881	2.58987619
C	2.67000919	6.74954185	0.66792363
C	1.21238281	6.39798117	0.99068990
H	0.78676442	7.11386410	1.69865775
H	0.60222303	6.46190638	0.08105737
C	2.59358736	5.69687613	-1.68409243

H	2.35278635	6.70250164	-2.04009500
H	3.22769839	5.24420171	-2.44960588
H	1.65307731	5.14870034	-1.68009221
H	3.23269675	6.70453879	1.60604384
C	2.81323369	8.25405352	0.13941552
H	2.37647430	8.23107113	-0.86379576
C	4.77944880	6.12217871	-0.55398894
H	5.36251442	6.08004248	0.36890250
H	5.26812397	5.45719946	-1.26968789
C	4.90399989	7.57325331	-1.11480059
H	4.38289072	7.64944746	-2.07253277
C	4.26373694	8.46413981	-0.11293219
H	5.95044592	7.85044733	-1.24868636
C	3.97542256	9.40405702	2.76068516
H	4.14272147	9.87479405	3.73444844
C	2.06743190	9.35309289	0.93412062
H	1.01750343	9.04284706	0.93929237
C	2.47413467	9.41687276	2.42358728
H	2.01299561	10.29834423	2.87141638
H	2.02491855	8.56504339	2.94255432
O	5.06720833	9.23291838	0.49326818
C	4.86734210	10.13269262	1.79008949
O	6.11700232	10.24871471	2.27841525
H	4.45950920	11.04602624	1.35558436
C	2.06037909	10.70770074	0.14008976
C	1.60652253	11.90705421	0.98626753
H	1.51863185	12.79606754	0.35573780
H	0.62355461	11.72698837	1.43594679
H	2.30120787	12.15297865	1.79478369
C	-1.97011406	-3.12082170	-0.35806196
H	-2.94028077	-3.32131702	-0.81038304
H	-1.20150442	-3.73124617	-0.84105173
H	-1.98464557	-3.39495494	0.70038945
C	1.15217620	10.60509518	-1.09965817
H	1.44277336	9.80499616	-1.78667173
H	0.11208948	10.42859005	-0.80388421
H	1.17943160	11.53785720	-1.66950528
H	3.07448014	10.92424868	-0.22694582
H	4.36969824	8.38663287	2.85604178
H	6.63428532	10.88688548	1.76990815

VII (Fig. 6S) input structure

1 1

C	0.00000000	0.00000000	0.00000000
C	1.54720649	0.00000000	0.00000000
C	1.98377350	1.50722698	0.00000000
C	1.38913102	2.42931875	1.12111756
C	-0.15647352	2.27224026	1.08480863
C	-0.62583875	0.81401746	1.12582413
H	-0.35095345	0.40354907	-0.95589768
H	-0.61386822	2.80680726	1.92354951
H	-0.54538984	2.73724041	0.16833514
H	-0.39500580	0.34806164	2.08941729
H	-1.71320600	0.78188349	1.01430165
H	1.53972302	1.89972985	-0.93006175
C	1.86621141	2.09049087	2.55617575
H	2.87843364	1.68564816	2.59833210
H	1.83609107	2.97094306	3.20282316
H	1.21655811	1.34696265	3.01989908
C	1.72928264	3.91477672	0.69370593
H	1.21815792	3.99704230	-0.27230373
C	3.49622822	1.72989577	-0.14416566
H	3.90715535	1.06503277	-0.90788758

H	4.02126811	1.47824643	0.78241691
C	3.80325661	3.17327410	-0.56868506
H	3.37738441	3.31182348	-1.56754128
H	4.88816296	3.29501687	-0.67108897
C	3.24011134	4.24239000	0.40187259
C	2.00518735	-0.66215205	-1.31980874
H	3.08169776	-0.85116806	-1.33268735
H	1.50722761	-1.62742476	-1.43937198
H	1.75609670	-0.04479065	-2.19029937
C	2.11952001	-0.83569503	1.16268304
H	1.73695669	-0.53952488	2.13952606
H	1.86443626	-1.88817055	1.01959068
H	3.21078726	-0.76234102	1.19430944
O	-0.45426514	-1.37117809	0.07914463
C	-1.64708209	-1.65174225	-0.50131031
O	-2.32876552	-0.82880880	-1.06110934
C	4.10331122	4.21255762	1.68727870
H	5.14947283	4.44578410	1.47342766
H	3.76051816	4.89105293	2.47052829
H	4.10702426	3.21614505	2.12185471
C	3.31717955	5.70618112	-0.28602638
C	1.09439384	4.98921258	1.59025840
H	0.03103034	4.78224353	1.73156971
H	1.53991570	4.99870830	2.58936018
C	2.68059530	6.74003889	0.69868346
C	1.21455860	6.39936071	0.99058793
H	0.78458781	7.10613923	1.70227526
H	0.62160461	6.47645559	0.07031515
C	2.60237735	5.71760084	-1.66442611
H	2.36709612	6.72916621	-2.00976247
H	3.23209199	5.26661148	-2.43493756
H	1.65821275	5.17686945	-1.66466393
H	3.22325430	6.69127161	1.64508877
C	2.82792004	8.26696354	0.18816279
H	2.29220268	8.26791147	-0.76648697
C	4.78655822	6.11298936	-0.52632916
H	5.36390576	6.09266149	0.40015437
H	5.28308874	5.44160544	-1.22999977
C	4.90699267	7.55661632	-1.12148227
H	4.38724687	7.59595464	-2.08297748
C	4.24581217	8.45129221	-0.16197466
H	5.94941826	7.84808221	-1.25803341
C	4.44933698	9.73405578	2.41247371
H	4.91260840	9.60053346	3.40042747
C	2.20342200	9.36284133	1.10228681
H	1.18572593	9.01533502	1.29868651
C	2.92842550	9.51360123	2.47975163
H	2.53080744	10.36661018	3.03979430
H	2.75906194	8.60968106	3.08550057
O	5.02235605	9.29354076	0.42564576
C	4.84206905	11.12285251	1.95489689
O	4.23988872	12.12935415	2.24532432
H	5.75841867	11.16868085	1.33255676
C	2.04618382	10.73486818	0.36808976
C	1.28533366	11.74868670	1.23822525
H	1.21224992	12.70764879	0.71856259
H	0.26345171	11.40465773	1.43537031
H	1.77091426	11.93896025	2.19859022
C	-1.98532863	-3.11251373	-0.34737195
H	-2.95219524	-3.31061831	-0.80777765
H	-1.21441559	-3.72873688	-0.81884067
H	-2.01262392	-3.38139646	0.71232938
C	1.34288802	10.61473224	-0.99438352

H	1.93693910	10.08617990	-1.74558417
H	0.37906551	10.10130775	-0.89904757
H	1.14248494	11.60985021	-1.39995521
H	3.04977272	11.15296430	0.18651479
H	4.95336616	9.00400331	1.76800334
H	4.84372884	12.87565706	2.24231142

VII (Fig. 6S) output structure, E = -1552.4307

1 1

C	0.00000000	0.00000000	0.00000000
C	1.54721270	0.00000000	0.00000000
C	1.98454475	1.50652849	0.00000000
C	1.39282700	2.42843576	1.12291722
C	-0.15331389	2.27056608	1.08896801
C	-0.62340046	0.81247255	1.12796638
H	-0.35175796	0.40486695	-0.95498631
H	-0.60992159	2.80418516	1.92883832
H	-0.54399142	2.73641299	0.17375162
H	-0.39188691	0.34359230	2.09012038
H	-1.71098173	0.78197875	1.01828018
H	1.53992640	1.90045229	-0.92892247
C	1.87119975	2.08437479	2.55627267
H	2.88550345	1.68496469	2.59714501
H	1.83555549	2.96029897	3.20941465
H	1.22572209	1.33461182	3.01595632
C	1.73395387	3.91431273	0.69806001
H	1.21584472	4.00145998	-0.26335979
C	3.49669993	1.72599338	-0.14787403
H	3.90404964	1.06026553	-0.91261919
H	4.02589131	1.47312314	0.77640904
C	3.80279402	3.16797558	-0.57576920
H	3.37446153	3.30407133	-1.57355782
H	4.88724391	3.29092223	-0.68195270
C	3.24343158	4.24117474	0.39312489
C	2.00578259	-0.66372716	-1.31870245
H	3.08258763	-0.85151733	-1.33199090
H	1.50914743	-1.62995245	-1.43579904
H	1.75510399	-0.04865295	-2.19034877
C	2.11921495	-0.83464050	1.16376411
H	1.72879179	-0.54534683	2.13985057
H	1.87354637	-1.88870222	1.01612757
H	3.20964048	-0.75195904	1.20052108
O	-0.45344274	-1.37131780	0.07798382
C	-1.64796674	-1.65135973	-0.49959123
O	-2.33242334	-0.82714136	-1.05390031
C	4.11579996	4.20884315	1.67255897
H	5.16171872	4.43697788	1.45297038
H	3.78265441	4.89226721	2.45633685
H	4.11603489	3.21543415	2.11442170
C	3.31569441	5.70160470	-0.29691813
C	1.10613534	4.99090345	1.59900868
H	0.04654249	4.77965868	1.75897388
H	1.56621824	4.99569093	2.59335929
C	2.68026969	6.74502456	0.68769946
C	1.21744365	6.39925768	0.99395232
H	0.78179166	7.11634874	1.69410976
H	0.62078112	6.46448859	0.07585472
C	2.59069739	5.70957341	-1.67022161
H	2.37897919	6.72061138	-2.02853203
H	3.20589384	5.23418771	-2.43726387
H	1.63438109	5.19038604	-1.65692060
H	3.23979720	6.69194714	1.62788837
C	2.83732090	8.25229940	0.16933928

H	2.37219741	8.24834481	-0.82001056
C	4.78568491	6.10947103	-0.55455877
H	5.37740094	6.06096993	0.36218756
H	5.26340082	5.44571663	-1.27856016
C	4.92081636	7.56755128	-1.11493685
H	4.39889555	7.64917361	-2.07072172
C	4.28303479	8.41925133	-0.09469058
H	5.96974512	7.83905469	-1.24157630
C	3.79242448	9.57635385	3.01324482
H	4.39876795	8.71593020	3.28649534
C	2.11671409	9.36303716	0.99170877
H	1.05445583	9.11589752	0.87909304
C	2.37712132	9.33898923	2.52729189
H	1.72066842	10.08647896	2.97829800
H	2.04928616	8.37460619	2.92493602
O	5.07209589	9.13353628	0.60566073
C	4.29873083	10.76946674	3.38801308
O	3.61033423	11.91448418	3.25152935
H	5.29332703	10.84628718	3.82286230
C	2.27899469	10.78773332	0.36757038
C	1.20029506	11.74397976	0.90461268
H	1.31637559	12.73270309	0.45167699
H	0.19696667	11.38217950	0.65217494
H	1.25696600	11.87785713	1.98581305
C	-1.98253299	-3.11355013	-0.35167071
H	-2.95796568	-3.30842004	-0.79507816
H	-1.22082688	-3.72395856	-0.84538954
H	-1.98762303	-3.39304572	0.70550034
C	2.22692181	10.79160692	-1.16975075
H	3.07302443	10.27621481	-1.63768630
H	1.30400544	10.33122918	-1.54238559
H	2.24843585	11.81987801	-1.54004738
H	3.25327344	11.19775861	0.66577571
H	4.64217886	9.55300744	1.43258090
H	4.12547170	12.66414407	3.57131913

VIII (Fig. 7S) input structure

1 1

C	0.00000000	0.00000000	0.00000000
C	1.54677895	0.00000000	0.00000000
C	1.99018105	1.50473672	0.00000000
C	1.38909759	2.43672037	1.10980561
C	-0.15629414	2.27588296	1.07188166
C	-0.62588801	0.81800239	1.12250864
H	-0.35218860	0.39902761	-0.95724734
H	-0.61779349	2.81909067	1.90320543
H	-0.54186110	2.73325305	0.15052194
H	-0.39317850	0.35627132	2.08795766
H	-1.71340951	0.78379485	1.01227307
H	1.56099624	1.89755035	-0.93640310
C	1.86389556	2.10118239	2.54613567
H	2.89269300	1.74118326	2.59471454
H	1.78791321	2.97323418	3.20153900
H	1.24242414	1.32591745	2.99669211
C	1.72976643	3.91904244	0.67498204
H	1.20160604	4.01260195	-0.28026584
C	3.50633079	1.71937371	-0.12574829
H	3.92742608	1.03830313	-0.86975158
H	4.01779234	1.48281788	0.81338840
C	3.81556617	3.15306456	-0.57525889
H	3.41128937	3.26401788	-1.58533920
H	4.90052283	3.28281101	-0.66233077
C	3.23229516	4.25128598	0.35058123

C	2.00368007	-0.66521142	-1.31853182
H	3.08007127	-0.85425677	-1.33191373
H	1.50510131	-1.63049023	-1.43621221
H	1.75469229	-0.04921173	-2.18970423
C	2.11647474	-0.83675600	1.16332409
H	1.72460226	-0.54709554	2.13882930
H	1.86947130	-1.89068120	1.01574662
H	3.20691583	-0.75572148	1.20267722
O	-0.45148799	-1.37311282	0.08558052
C	-1.64472377	-1.66082522	-0.48785097
O	-2.33615472	-0.84382817	-1.04445085
C	4.09957284	4.25886012	1.63593444
H	5.13892710	4.51385002	1.41699361
H	3.74478402	4.95445811	2.40027874
H	4.11842195	3.27273566	2.09570985
C	3.28818098	5.68260655	-0.38072800
C	1.12770143	5.00069093	1.59141759
H	0.06824023	4.79987943	1.77057278
H	1.60511688	4.98294875	2.57891318
C	2.69445905	6.76808340	0.57764796
C	1.25602842	6.40569840	0.99149471
H	0.86480374	7.13574203	1.71032012
H	0.60109734	6.48510266	0.11569077
C	2.51106798	5.69124669	-1.72398511
H	2.59012354	6.67137336	-2.19936535
H	2.92963297	4.97467465	-2.43258484
H	1.44953175	5.46616293	-1.62185698
H	3.31828085	6.75656595	1.48070817
C	2.75736843	8.22124633	0.02348080
H	1.91171728	8.35708363	-0.66078510
C	4.74975740	6.07746098	-0.72377339
H	5.42747866	5.86131004	0.10975584
H	5.10891635	5.45810721	-1.55328210
C	4.89651876	7.52403074	-1.10487910
C	4.00436057	8.46669478	-0.78871577
H	5.76773366	7.80035569	-1.69771434
C	5.12291727	8.74699394	1.32076006
H	6.00941118	8.81389545	1.98930904
C	2.58079741	9.28994031	1.16871927
H	1.97194187	8.81179865	1.94811664
C	3.96322450	9.62181397	1.79984643
H	4.21502570	10.66944574	1.60541956
H	3.90385309	9.53023449	2.88594011
O	4.11640690	9.77626419	-1.18737587
C	5.69932133	8.98273718	0.01359887
O	5.97486147	10.16135229	-0.38685062
H	5.92769979	8.14696106	-0.65375507
C	1.77829879	10.53732230	0.68403299
C	1.83265080	11.74663895	1.63165470
H	1.26207878	12.57776266	1.20694555
H	1.37176630	11.52487990	2.60466176
H	2.84876814	12.12216438	1.79320910
C	-1.97080416	-3.12500526	-0.33451622
H	-2.95240966	-3.32411210	-0.76222739
H	-1.21506007	-3.73160770	-0.84209130
H	-1.95642596	-3.40587907	0.72216481
C	0.30262990	10.18122680	0.43833551
H	0.17427549	9.35584919	-0.26567308
H	-0.19411806	9.89905712	1.37487608
H	-0.23381576	11.04228402	0.02938971
H	2.22248036	10.85087957	-0.26550894
H	4.91522643	7.66335727	1.36112679
H	4.87189419	9.86223601	-1.78273188

H	6.37581445	10.20074509	-1.27529078

VIII (Fig. 7S) output structure, E = -1552.4407

1 1

C	0.00000000	0.00000000	0.00000000
C	1.54692122	0.00000000	0.00000000
C	1.98692614	1.50621933	0.00000000
C	1.39214637	2.43202759	1.11690389
C	-0.15391215	2.27863757	1.07330519
C	-0.62649132	0.82138488	1.12014685
H	-0.35193473	0.39618458	-0.95859536
H	-0.61639877	2.81938007	1.90528961
H	-0.53609427	2.73877815	0.15171611
H	-0.39918532	0.35936267	2.08639898
H	-1.71366295	0.79119418	1.00655864
H	1.54635669	1.89868334	-0.93181777
C	1.86097625	2.08621076	2.55263370
H	2.87857468	1.69526327	2.59910008
H	1.81119201	2.95932516	3.20798060
H	1.21913027	1.32881093	3.00447615
C	1.73824962	3.92026230	0.69872306
H	1.21154998	4.02348594	-0.25824525
C	3.49981044	1.72679580	-0.14179527
H	3.91265819	1.05177749	-0.89532897
H	4.02501685	1.49098097	0.78929374
C	3.79326679	3.16542844	-0.58809688
H	3.34134702	3.28667066	-1.57734618
H	4.87458632	3.29314340	-0.72090360
C	3.24757538	4.24408915	0.38223913
C	2.00487416	-0.66246317	-1.31981571
H	3.08167141	-0.85098186	-1.33375713
H	1.50726637	-1.62808998	-1.43867733
H	1.75478376	-0.04520925	-2.19012188
C	2.11796907	-0.83732963	1.16258004
H	1.72439410	-0.55151090	2.13818505
H	1.87336819	-1.89113891	1.01127353
H	3.20826568	-0.75459331	1.20371212
O	-0.45196195	-1.37123245	0.09000845
C	-1.64327075	-1.65995761	-0.48966721
O	-2.32454702	-0.84469567	-1.06084705
C	4.13059659	4.21139410	1.65439370
H	5.18878643	4.32305985	1.40309594
H	3.87868855	4.98019472	2.38851089
H	4.04195258	3.25674231	2.16523158
C	3.32596374	5.69807553	-0.30010946
C	1.12864058	4.98935721	1.62273758
H	0.07851870	4.76519977	1.82125387
H	1.62661091	4.99952230	2.59849935
C	2.65591508	6.75466276	0.63362498
C	1.21454727	6.38793289	1.00268248
H	0.81248464	7.12862234	1.70239527
H	0.58046116	6.43623987	0.10715881
C	2.66970770	5.70915086	-1.70337989
H	2.52042364	6.72508997	-2.09230932
H	3.28805622	5.19092660	-2.43936257
H	1.68243413	5.24783977	-1.71633191
H	3.23626185	6.75264300	1.56182832
C	2.72657659	8.21004301	0.07306992
H	1.75899992	8.51476562	-0.33248403
C	4.79825819	6.15643310	-0.46095240
H	5.28523642	6.16643067	0.51465554
H	5.36053949	5.45615962	-1.08341422
C	4.92933799	7.55630800	-1.09816784

C	3.71167792	8.38742023	-0.98758801
H	5.18105561	7.47933056	-2.16109719
C	5.75692504	8.90532873	0.97248687
H	6.65595172	9.42342637	1.32015227
C	3.10823483	9.32616324	1.18826572
H	2.98219575	8.78558534	2.13339754
C	4.56489948	9.87131848	1.14994765
H	4.64424215	10.66054560	0.39421521
H	4.71898573	10.37396102	2.10616023
O	3.59358510	9.42185025	-1.74559796
C	6.05507235	8.45051190	-0.46209044
O	6.20635285	9.60303109	-1.30699320
H	6.96891257	7.84881587	-0.47084727
C	2.08770613	10.50194459	1.15137439
C	2.45509137	11.62085698	2.14441672
H	1.66790861	12.37951563	2.14680942
H	2.53646208	11.23335902	3.16677253
H	3.38924831	12.12680134	1.89430258
C	-1.97894283	-3.11968622	-0.32049890
H	-2.95367973	-3.32063649	-0.76269467
H	-1.21692576	-3.73805851	-0.80380984
H	-1.98583001	-3.38332468	0.74071029
C	0.64708748	10.05093407	1.43831513
H	0.26477047	9.30882483	0.73209401
H	0.56232408	9.62837977	2.44607577
H	-0.02738893	10.90982809	1.38680473
H	2.12243423	10.92947246	0.13903602
H	5.65017304	8.02404401	1.61393417
H	4.46542705	9.61689708	-2.16807831
H	7.01741442	9.52681622	-1.82188754

IX (Fig. 7S) input structure

1 1

C	0.00000000	0.00000000	0.00000000
C	1.54677919	0.00000000	0.00000000
C	1.99018096	1.50473706	0.00000000
C	1.38909712	2.43672081	1.10980516
C	-0.15629440	2.27588345	1.07188097
C	-0.62588813	0.81800290	1.12250917
H	-0.35218870	0.39902685	-0.95724757
H	-0.61779406	2.81909129	1.90320495
H	-0.54186082	2.73325305	0.15052140
H	-0.39317832	0.35627150	2.08795800
H	-1.71340917	0.78379423	1.01227347
H	1.56099625	1.89755018	-0.93640354
C	1.86389585	2.10118351	2.54613500
H	2.89269282	1.74118420	2.59471426
H	1.78791262	2.97323575	3.20153851
H	1.24242382	1.32591821	2.99669231
C	1.72976599	3.91904326	0.67498061
H	1.20160509	4.01260188	-0.28026669
C	3.50633020	1.71937402	-0.12574871
H	3.92742601	1.03830328	-0.86975177
H	4.01779243	1.48281816	0.81338792
C	3.81556607	3.15306489	-0.57525940
H	3.41128878	3.26401826	-1.58534000
H	4.90052274	3.28281137	-0.66233208
C	3.23229503	4.25128631	0.35057993
C	2.00368013	-0.66521115	-1.31853102
H	3.08007149	-0.85425687	-1.33191277
H	1.50510131	-1.63049039	-1.43621120
H	1.75469220	-0.04921244	-2.18970429
C	2.11647527	-0.83675583	1.16332505

H	1.72460299	-0.54709433	2.13883021
H	1.86947164	-1.89068034	1.01574804
H	3.20691650	-0.75572026	1.20267721
O	-0.45148721	-1.37311305	0.08558127
C	-1.64472385	-1.66082615	-0.48785024
O	-2.33615423	-0.84382869	-1.04445022
C	4.09957228	4.25886168	1.63593327
H	5.13892669	4.51385128	1.41699216
H	3.74478352	4.95445907	2.40027668
H	4.11842128	3.27273684	2.09570868
C	3.28817996	5.68260643	-0.38072956
C	1.12770095	5.00069164	1.59141595
H	0.06823950	4.79987971	1.77057159
H	1.60511646	4.98295000	2.57891135
C	2.69445756	6.76808390	0.57764578
C	1.25602769	6.40569902	0.99149263
H	0.86480250	7.13574310	1.71031796
H	0.60109609	6.48510244	0.11568925
C	2.51106745	5.69124608	-1.72398666
H	2.59012261	6.67137298	-2.19936726
H	2.92963252	4.97467378	-2.43258608
H	1.44953036	5.46616223	-1.62185885
H	3.31828033	6.75656688	1.48070597
C	2.75736695	8.22124722	0.02347788
H	1.91171557	8.35708317	-0.66078784
C	4.74975692	6.07746153	-0.72377537
H	5.42747786	5.86131131	0.10975420
H	5.10891545	5.45810729	-1.55328374
C	4.89651716	7.52403101	-1.10488207
C	4.00435911	8.46669504	-0.78871850
H	5.76773240	7.80035548	-1.69771698
C	5.12291640	8.74699515	1.32075675
H	6.00940963	8.81389700	1.98930568
C	2.58079599	9.28994117	1.16871580
H	1.97194035	8.81180005	1.94811407
C	3.96322284	9.62181520	1.79984323
H	4.21502396	10.66944710	1.60541583
H	3.90385219	9.53023642	2.88593653
O	4.11640502	9.77626399	-1.18737898
C	5.69931934	8.98273784	0.01359552
O	6.41393537	8.09962378	-0.56506734
H	5.51709158	9.92290509	-0.51453169
C	1.77829748	10.53732297	0.68402903
C	1.83264860	11.74663942	1.63165037
H	1.26207701	12.57776339	1.20694079
H	1.37176457	11.52488090	2.60465732
H	2.84876582	12.12216533	1.79320467
C	-1.97080409	-3.12500619	-0.33451490
H	-2.95240923	-3.32411306	-0.76222552
H	-1.21505900	-3.73160835	-0.84208906
H	-1.95642463	-3.40587955	0.72216688
C	0.30262799	10.18122649	0.43833182
H	0.17427428	9.35584850	-0.26567591
H	-0.19411985	9.89905749	1.37487255
H	-0.23381773	11.04228431	0.02938589
H	2.22247849	10.85088013	-0.26551289
H	4.91522532	7.66335824	1.36112456
H	4.87189268	9.86223631	-1.78273538
H	6.73517835	8.35830475	-1.44910854

IX (Fig. 7S) output structure, E = -1552.4319

1 1

C	0.00000000	0.00000000	0.00000000

C	1.54684660	0.00000000	0.00000000
C	1.98413832	1.50725901	0.00000000
C	1.39160640	2.42979502	1.12110066
C	-0.15434737	2.27653448	1.07947604
C	-0.62755663	0.81911982	1.12109736
H	-0.35114676	0.39813756	-0.95809134
H	-0.61581718	2.81427981	1.91398111
H	-0.53740452	2.74022595	0.16006762
H	-0.40352271	0.35379459	2.08673395
H	-1.71462484	0.78939207	1.00468157
H	1.54025403	1.89990483	-0.92999311
C	1.86302826	2.07814105	2.55464794
H	2.87511098	1.67266753	2.59720454
H	1.82782484	2.95085047	3.21142741
H	1.21244361	1.32984130	3.00896889
C	1.73373579	3.92070807	0.70845421
H	1.19683859	4.03291232	-0.24213640
C	3.49603462	1.73227614	-0.14670384
H	3.90944722	1.05876593	-0.90143269
H	4.02557903	1.49941336	0.78272161
C	3.78179579	3.17119468	-0.59474850
H	3.32344371	3.29064177	-1.58140742
H	4.86147490	3.30432332	-0.73328836
C	3.23888949	4.24805010	0.37919601
C	2.00384471	-0.66153740	-1.32044450
H	3.08034788	-0.85103768	-1.33456695
H	1.50561872	-1.62672145	-1.43971744
H	1.75369902	-0.04402037	-2.19054960
C	2.12191326	-0.83564244	1.16095839
H	1.72683851	-0.55352015	2.13694902
H	1.88439030	-1.89082151	1.00831449
H	3.21173272	-0.74466801	1.20082753
O	-0.45273859	-1.37126355	0.08715885
C	-1.63869745	-1.66106846	-0.50278309
O	-2.31679788	-0.84617221	-1.07832298
C	4.12998214	4.21475522	1.64715034
H	5.19049122	4.28201856	1.39106755
H	3.91097129	5.00875954	2.36432456
H	4.01025045	3.27730448	2.18337357
C	3.30900760	5.70487434	-0.30530375
C	1.13586848	4.98328058	1.64885418
H	0.08758123	4.76012728	1.85857301
H	1.64583460	4.98556743	2.61822019
C	2.65055131	6.75330259	0.64312323
C	1.21572446	6.38494369	1.03550524
H	0.82414635	7.12406768	1.74381996
H	0.56661245	6.43772378	0.15128261
C	2.62612313	5.72270837	-1.69524894
H	2.54224795	6.73668029	-2.10790361
H	3.19580067	5.14836040	-2.42782567
H	1.60978562	5.32878334	-1.68150650
H	3.24577028	6.76347515	1.56053543
C	2.68166176	8.20778546	0.07261189
H	1.73176744	8.42976029	-0.41957776
C	4.78053896	6.14612452	-0.49490822
H	5.34240504	6.02597456	0.42844403
H	5.27439568	5.51381546	-1.23621357
C	4.96034495	7.62199837	-0.96163766
C	3.71536065	8.38684764	-0.95111324
H	5.39380283	7.66855129	-1.96894657
C	5.46523876	8.76946797	1.36902034
H	6.32245544	9.17705885	1.91267441
C	2.87313898	9.32593151	1.20557987

H	2.58691684	8.81049301	2.13021698
C	4.33321357	9.81480902	1.37232716
H	4.55081027	10.57943218	0.61241852
H	4.38176415	10.34387236	2.32550493
O	3.50136625	9.33092499	-1.81413966
C	6.00756109	8.41357680	-0.01378779
O	7.13906771	7.61549732	0.16648468
H	6.25838022	9.34303854	-0.54594661
C	1.89137256	10.51411810	0.99793602
C	2.13554123	11.64702285	2.01185288
H	1.37419877	12.42158122	1.88693177
H	2.06044589	11.27979053	3.04209775
H	3.10845314	12.12726545	1.89050373
C	-1.97298604	-3.12170181	-0.33867549
H	-2.94352472	-3.32399497	-0.78940989
H	-1.20564865	-3.73788870	-0.81635788
H	-1.98831231	-3.38712826	0.72200005
C	0.41851161	10.08426920	1.08800487
H	0.13459176	9.31744810	0.36139659
H	0.18539173	9.69832263	2.08689887
H	-0.23279183	10.94405717	0.90952261
H	2.07283097	10.92133423	-0.00754396
H	5.18112016	7.85900772	1.90621199
H	4.22068709	9.41122338	-2.46560607
H	7.66993918	7.59407267	-0.63905355

X (Fig. 8S), E = -1552.4359

1 1

C	0.00000000	0.00000000	0.00000000
C	1.54673259	0.00000000	0.00000000
C	1.98550759	1.50708823	0.00000000
C	1.39141921	2.43060888	1.11977499
C	-0.15413609	2.27565861	1.07923394
C	-0.62568147	0.81783406	1.12255158
H	-0.35106994	0.39846893	-0.95781373
H	-0.61497020	2.81353204	1.91397515
H	-0.53946814	2.73813099	0.16017605
H	-0.39607212	0.35282910	2.08705921
H	-1.71315011	0.78617770	1.01044174
H	1.54306141	1.90028094	-0.93032180
C	1.86276380	2.08468734	2.55504443
H	2.87534483	1.68120411	2.59806732
H	1.82855415	2.96195778	3.20598269
H	1.21267467	1.33840314	3.01387201
C	1.73754571	3.91775049	0.70138885
H	1.22184221	4.01449601	-0.26149942
C	3.49810830	1.72844728	-0.14353892
H	3.90859363	1.06130829	-0.90565338
H	4.02326848	1.47835018	0.78400912
C	3.80086117	3.17126987	-0.57132750
H	3.36474863	3.30898277	-1.56567855
H	4.88437953	3.29366949	-0.68744968
C	3.24867992	4.24523198	0.40092363
C	2.00301153	-0.66275428	-1.32010037
H	3.07960359	-0.85137283	-1.33534368
H	1.50502874	-1.62826468	-1.43861879
H	1.75164815	-0.04531600	-2.18992010
C	2.11993220	-0.83604200	1.16228372
H	1.73091355	-0.54783077	2.13922982
H	1.87488998	-1.89024966	1.01421715
H	3.21035021	-0.75251506	1.19801766
O	-0.45283086	-1.37187152	0.08638243
C	-1.64009187	-1.66113300	-0.49973341

O	-2.32111613	-0.84652953	-1.07240303
C	4.11912652	4.20033514	1.68090660
H	5.17348300	4.37299548	1.44989858
H	3.82096367	4.92284502	2.44391456
H	4.07305564	3.22150386	2.15072003
C	3.33680346	5.70379613	-0.27626173
C	1.10939672	4.99043643	1.60832899
H	0.05338080	4.77009090	1.77910603
H	1.58120915	4.99914094	2.59743590
C	2.67444179	6.75549589	0.66948926
C	1.21906864	6.38965382	0.99180252
H	0.78359950	7.12146296	1.68106099
H	0.61784424	6.44169194	0.07464790
C	2.66239852	5.71541318	-1.67456484
H	2.41357652	6.72769394	-2.00491364
H	3.32062377	5.28699855	-2.43521457
H	1.73000714	5.15306549	-1.70371897
H	3.23062386	6.70998915	1.61569449
C	2.77881234	8.23642545	0.18922744
H	2.07265643	8.39112006	-0.63247370
C	4.81274973	6.14138059	-0.45839143
H	5.32667145	6.14857435	0.50769027
H	5.35423889	5.43018086	-1.08769332
C	4.93782815	7.53090942	-1.09441006
H	4.52817962	7.49838737	-2.11116698
C	4.18748600	8.61183543	-0.32023926
H	5.99300662	7.80983393	-1.20538838
C	4.89789742	9.15945559	0.94266646
H	5.18537325	8.33947481	1.60493270
C	2.47243843	9.22698574	1.36385725
H	2.28515288	8.61867798	2.25429596
C	3.78072093	10.02166638	1.64652656
H	3.74604514	11.01512792	1.19353972
H	3.98566430	10.14426971	2.71060079
O	4.09484584	9.82121815	-1.18365719
H	3.84971339	9.55944576	-2.08158114
C	6.06257915	10.02082215	0.69116970
O	6.18175268	10.69843503	-0.35738050
H	6.85447778	10.15390011	1.43388248
C	1.21987188	10.12533815	1.19328113
H	0.39107980	9.42746721	1.01340500
C	1.26453263	11.09292261	0.00264481
H	2.03163608	11.86575103	0.12419946
H	1.44996769	10.58432381	-0.94705875
H	0.30549062	11.61103777	-0.08573062
C	0.90883196	10.88064917	2.49422983
H	-0.03837571	11.42029407	2.40905457
H	0.82808083	10.19919298	3.34743031
H	1.68201152	11.62202832	2.72743359
C	-1.97335684	-3.12234798	-0.33616726
H	-2.94688468	-3.32378104	-0.78085785
H	-1.20928398	-3.73736588	-0.82055842
H	-1.98138493	-3.38999432	0.72401445
H	5.32227279	10.45430962	-0.96279832

XI (Fig. 8S), E = -1552.4116

1 1

C	0.00000000	0.00000000	0.00000000
C	1.54678531	0.00000000	0.00000000
C	1.98527864	1.50705346	0.00000000
C	1.39210963	2.43035150	1.12048443
C	-0.15330914	2.27528312	1.08052393
C	-0.62576949	0.81764067	1.12270238

H	-0.35148407	0.39821062	-0.95775045
H	-0.61362382	2.81260139	1.91588165
H	-0.53852187	2.73901093	0.16210966
H	-0.39719445	0.35168175	2.08715325
H	-1.71323371	0.78647212	1.00983391
H	1.54245810	1.90055927	-0.92990494
C	1.86411911	2.08360925	2.55539729
H	2.87304652	1.67085562	2.59723036
H	1.84037023	2.96284294	3.20413692
H	1.20819191	1.34468898	3.01816553
C	1.73764109	3.91768023	0.70232276
H	1.22246048	4.01475728	-0.26081146
C	3.49754112	1.72876746	-0.14493644
H	3.90714629	1.06277452	-0.90872401
H	4.02422890	1.47657282	0.78139613
C	3.80022271	3.17196526	-0.57071845
H	3.36541184	3.31145193	-1.56527276
H	4.88367280	3.29414305	-0.68613252
C	3.24825292	4.24654017	0.40104284
C	2.00371638	-0.66340045	-1.31947784
H	3.07999524	-0.85391539	-1.33254594
H	1.50414048	-1.62796709	-1.43921523
H	1.75562422	-0.04522115	-2.18965253
C	2.11978332	-0.83491329	1.16281218
H	1.72894160	-0.54600399	2.13873409
H	1.87656356	-1.88960908	1.01511892
H	3.21001443	-0.74885386	1.19917783
O	-0.45280367	-1.37248616	0.08652561
C	-1.63978367	-1.66257430	-0.49861908
O	-2.32268953	-0.84909381	-1.07076010
C	4.11896176	4.19992799	1.68128018
H	5.17271302	4.37582830	1.45026699
H	3.81907260	4.92085458	2.44515945
H	4.07552038	3.22029110	2.15026353
C	3.33788109	5.70505101	-0.27610334
C	1.10784579	4.98998594	1.60925960
H	0.05124633	4.76991302	1.77764516
H	1.57735700	4.99654646	2.59974389
C	2.67571425	6.75686335	0.67148015
C	1.22117150	6.39015831	0.99539087
H	0.78765352	7.12179599	1.68677093
H	0.61842866	6.44620502	0.07943596
C	2.65880401	5.71838210	-1.67186295
H	2.43070337	6.73289239	-2.00789363
H	3.30768527	5.27428647	-2.43131796
H	1.71600280	5.17285784	-1.69501960
H	3.23101551	6.70739788	1.61934580
C	2.78614032	8.23067405	0.18502044
H	2.08461034	8.38348899	-0.64059503
C	4.81404569	6.14105876	-0.46691900
H	5.33250173	6.13777732	0.49761201
H	5.34672532	5.42538369	-1.09941219
C	4.94625791	7.53310246	-1.09533136
H	4.54368411	7.51742423	-2.11440311
C	4.19942665	8.63029036	-0.33166126
H	6.00110719	7.81198705	-1.20468315
C	4.88081185	9.08864904	0.97625024
H	5.01063294	8.26068047	1.68064199
C	2.47198689	9.23477402	1.34405744
H	2.29114649	8.64315056	2.24784181
C	3.77214938	10.04801811	1.62596303
H	3.78068494	10.99996072	1.09547697
H	3.95171796	10.21804938	2.68812563

O	4.12923537	9.82529692	-1.11132586
H	3.72808402	9.61096967	-1.96143210
C	6.09566013	9.85558927	0.97603880
O	6.52959204	10.49299036	-0.04260316
H	6.66147930	9.95596262	1.90729694
C	1.22200354	10.13627910	1.16195600
H	0.39739405	9.43174105	0.98696921
C	1.26975291	11.08999924	-0.03877185
H	2.03357876	11.86607954	0.07903452
H	1.46834014	10.57119731	-0.97921464
H	0.30781426	11.60096872	-0.13861773
C	0.90344922	10.90352164	2.45404487
H	-0.04831873	11.43396963	2.36142363
H	0.82727617	10.23203294	3.31572838
H	1.66941036	11.65528082	2.67839255
C	-1.97139796	-3.12438135	-0.33500617
H	-2.94376309	-3.32739637	-0.78154668
H	-1.20542923	-3.73872918	-0.81722575
H	-1.98144832	-3.39134896	0.72535525
H	7.32714068	11.02048507	0.14528565

XII (Fig. 8S), input structure

1 1

C	0.00000000	0.00000000	0.00000000
C	1.54673259	0.00000000	0.00000000
C	1.98550759	1.50708823	0.00000000
C	1.39141921	2.43060888	1.11977499
C	-0.15413609	2.27565861	1.07923394
C	-0.62568147	0.81783406	1.12255158
H	-0.35106994	0.39846893	-0.95781373
H	-0.61497020	2.81353204	1.91397515
H	-0.53946814	2.73813099	0.16017605
H	-0.39607212	0.35282910	2.08705921
H	-1.71315011	0.78617770	1.01044174
H	1.54306141	1.90028094	-0.93032180
C	1.86276380	2.08468734	2.55504443
H	2.87534483	1.68120411	2.59806732
H	1.82855415	2.96195778	3.20598269
H	1.21267467	1.33840314	3.01387201
C	1.73754571	3.91775049	0.70138885
H	1.22184221	4.01449601	-0.26149942
C	3.49810830	1.72844728	-0.14353892
H	3.90859363	1.06130829	-0.90565338
H	4.02326848	1.47835018	0.78400912
C	3.80086117	3.17126987	-0.57132750
H	3.36474863	3.30898277	-1.56567855
H	4.88437953	3.29366949	-0.68744968
C	3.24867992	4.24523198	0.40092363
C	2.00301153	-0.66275428	-1.32010037
H	3.07960359	-0.85137283	-1.33534368
H	1.50502874	-1.62826468	-1.43861879
H	1.75164815	-0.04531600	-2.18992010
C	2.11993220	-0.83604200	1.16228372
H	1.73091355	-0.54783077	2.13922982
H	1.87488998	-1.89024966	1.01421715
H	3.21035021	-0.75251506	1.19801766
O	-0.45283086	-1.37187152	0.08638243
C	-1.64009187	-1.66113300	-0.49973341
O	-2.32111613	-0.84652953	-1.07240303
C	4.11912652	4.20033514	1.68090660
H	5.17348300	4.37299548	1.44989858
H	3.82096367	4.92284502	2.44391456

H	4.07305564	3.22150386	2.15072003
C	3.33680346	5.70379613	-0.27626173
C	1.10939672	4.99043643	1.60832899
H	0.05338080	4.77009090	1.77910603
H	1.58120915	4.99914094	2.59743590
C	2.67444179	6.75549589	0.66948926
C	1.21906864	6.38965382	0.99180252
H	0.78359950	7.12146296	1.68106099
H	0.61784424	6.44169194	0.07464790
C	2.66239852	5.71541318	-1.67456484
H	2.41357652	6.72769394	-2.00491364
H	3.32062377	5.28699855	-2.43521457
H	1.73000714	5.15306549	-1.70371897
H	3.23062386	6.70998915	1.61569449
C	2.77881234	8.23642545	0.18922744
H	2.07265643	8.39112006	-0.63247370
C	4.81274973	6.14138059	-0.45839143
H	5.32667145	6.14857435	0.50769027
H	5.35423889	5.43018086	-1.08769332
C	4.93782815	7.53090942	-1.09441006
H	4.52817962	7.49838737	-2.11116698
C	4.18748600	8.61183543	-0.32023926
H	5.99300662	7.80983393	-1.20538838
C	4.89789742	9.15945559	0.94266646
H	5.18537325	8.33947481	1.60493270
C	2.47243843	9.22698574	1.36385725
H	2.28515288	8.61867798	2.25429596
C	3.78072093	10.02166638	1.64652656
H	3.74604514	11.01512792	1.19353972
H	3.98566430	10.14426971	2.71060079
O	4.09484584	9.82121815	-1.18365719
C	6.06257915	10.02082215	0.69116970
O	6.18175268	10.69843503	-0.35738050
H	6.85447778	10.15390011	1.43388248
C	1.21987188	10.12533815	1.19328113
H	0.39107980	9.42746721	1.01340500
C	1.26453263	11.09292261	0.00264481
H	2.03163608	11.86575103	0.12419946
H	1.44996769	10.58432381	-0.94705875
H	0.30549062	11.61103777	-0.08573062
C	0.90883196	10.88064917	2.49422983
H	-0.03837571	11.42029407	2.40905457
H	0.82808083	10.19919298	3.34743031
H	1.68201152	11.62202832	2.72743359
C	-1.97335684	-3.12234798	-0.33616726
H	-2.94688468	-3.32378104	-0.78085785
H	-1.20928398	-3.73736588	-0.82055842
H	-1.98138493	-3.38999432	0.72401445
H	6.51166302	11.57276550	-0.13759561
H	4.27027675	10.68959045	-0.81385515

XII (Fig. 8S), output structure, E = -1552.4262

1 1

C	0.00000000	0.00000000	0.00000000
C	1.54711864	0.00000000	0.00000000
C	1.98535583	1.50649876	0.00000000
C	1.39315907	2.42881824	1.12177631
C	-0.15248338	2.27649274	1.07919263
C	-0.62713223	0.81964614	1.12090823
H	-0.35147287	0.39767540	-0.95820569
H	-0.61373532	2.81455656	1.91370341
H	-0.53442883	2.74083084	0.15950369
H	-0.40278001	0.35434219	2.08649226

H	-1.71415421	0.79076169	1.00471328
H	1.54151548	1.90019478	-0.92953682
C	1.86463710	2.08199932	2.55654680
H	2.87158606	1.66451586	2.59829417
H	1.84483054	2.96140592	3.20533795
H	1.20597921	1.34624056	3.02023561
C	1.73948171	3.91559934	0.70413887
H	1.22590524	4.01156529	-0.26029074
C	3.49746182	1.72780629	-0.14790944
H	3.90497775	1.06395341	-0.91445060
H	4.02766080	1.47390661	0.77577083
C	3.79973605	3.17151272	-0.57251530
H	3.36131728	3.31281835	-1.56547464
H	4.88307579	3.29228850	-0.69065561
C	3.25063572	4.24243087	0.40505084
C	2.00439176	-0.66333466	-1.31930271
H	3.08007242	-0.85705994	-1.33088864
H	1.50268308	-1.62660710	-1.43962644
H	1.75885531	-0.04469630	-2.18990067
C	2.12018930	-0.83470843	1.16286040
H	1.72745615	-0.54770517	2.13850268
H	1.87811490	-1.88931453	1.01346529
H	3.21039204	-0.74815038	1.20132910
O	-0.45238577	-1.37166282	0.08756583
C	-1.64433146	-1.65922350	-0.49060868
O	-2.32888278	-0.84230367	-1.05564045
C	4.12721677	4.19436836	1.68103928
H	5.17914876	4.37379676	1.44498859
H	3.83075605	4.91069718	2.45038372
H	4.08708739	3.21266343	2.14575342
C	3.34167796	5.70237590	-0.26760058
C	1.10600493	4.98917485	1.60705059
H	0.04762561	4.77235304	1.76606172
H	1.56695500	4.99623151	2.60130910
C	2.68790931	6.74586987	0.69322873
C	1.22553354	6.38916530	0.99332468
H	0.78034985	7.12062651	1.67708551
H	0.63669733	6.44276282	0.06799832
C	2.65694028	5.72692148	-1.66146115
H	2.42695331	6.74304992	-1.99679795
H	3.30119457	5.28350899	-2.42509893
H	1.71339709	5.18393523	-1.68332779
H	3.23689996	6.68742772	1.63877957
C	2.80237801	8.23512941	0.23802839
H	2.01609016	8.40947062	-0.51716780
C	4.81655371	6.13683937	-0.45826427
H	5.32973309	6.18342238	0.50436009
H	5.35949455	5.41669235	-1.07480894
C	4.92831461	7.50927888	-1.12373722
H	4.61053198	7.45642175	-2.17731958
C	4.04505274	8.54406659	-0.52834758
H	5.95161504	7.89391405	-1.16201659
C	5.00027697	9.02856732	1.94761239
H	4.94759505	8.08462750	2.47975527
C	2.51791145	9.24053655	1.42800957
H	2.17200472	8.58947422	2.23608776
C	3.80226649	9.93041918	1.96164692
H	4.03864258	10.85020476	1.41882283
H	3.59930010	10.24740850	2.99053541
O	4.25875832	9.73482368	-0.98923443
C	6.20290609	9.36332349	1.44206712
O	6.40545899	10.53590941	0.81071686
H	7.05545582	8.69187450	1.51329179

C	1.34498157	10.22221958	1.15587920
H	0.48483690	9.59498013	0.88681137
C	1.57882300	11.18859057	-0.01661009
H	2.43958803	11.85079486	0.15218814
H	1.69298984	10.67270573	-0.98006156
H	0.71944507	11.85244242	-0.14477885
C	0.96166291	11.00054276	2.42394270
H	0.03446801	11.55725854	2.26242692
H	0.80102609	10.32828934	3.27277599
H	1.73123142	11.72547636	2.70789521
C	-1.97709144	-3.12049514	-0.32815969
H	-2.94989833	-3.32217611	-0.77427596
H	-1.21181951	-3.73545344	-0.81060241
H	-1.98732824	-3.38787899	0.73212856
H	7.32206969	10.61428772	0.52269771
H	3.60262303	10.38600438	-0.67552134

XIII (Fig.9S) E = -1552.4231

1 1

C	-5.16159100	0.44168100	-0.22684200
C	-4.63840600	-0.98008600	0.08695000
C	-3.07532700	-0.90665100	-0.02903200
C	-2.34169100	0.19350200	0.81421700
C	-3.01114900	1.55601000	0.48332300
C	-4.53732900	1.54030400	0.62449300
H	-4.96344800	0.66447000	-1.28078300
H	-2.61204900	2.34533700	1.12850600
H	-2.76041600	1.83977400	-0.54809200
H	-4.84029800	1.41243900	1.66910600
H	-4.93992200	2.50310900	0.29747100
H	-2.91259100	-0.59336400	-1.07368500
C	-2.42023000	-0.01242800	2.34818100
H	-2.46778900	-1.06006900	2.64850300
H	-1.56253200	0.43723200	2.85491900
H	-3.30665100	0.46831900	2.76440500
C	-0.85395700	0.24739900	0.27665000
H	-0.99706700	0.52130900	-0.77572500
C	-2.36469400	-2.26108000	0.10382900
H	-2.89606100	-3.02618000	-0.46765200
H	-2.36866600	-2.61058200	1.14138600
C	-0.93161200	-2.18369900	-0.44087900
H	-1.00621700	-1.97303200	-1.51241700
H	-0.45894900	-3.16894500	-0.34938000
C	-0.05365800	-1.10832100	0.24947800
C	-5.18676900	-1.92186200	-1.00938900
H	-5.00524000	-2.97419200	-0.77599500
H	-6.26842800	-1.79370400	-1.09679100
H	-4.74209500	-1.70666500	-1.98762200
C	-5.15772500	-1.49486400	1.44445100
H	-4.96234300	-0.81215400	2.27170300
H	-6.23894700	-1.64141100	1.39366300
H	-4.70472500	-2.45988100	1.69130300
O	-6.59743700	0.44488500	-0.04950100
C	-7.30498700	1.31113700	-0.81579300
O	-6.80263100	2.06233800	-1.61483400
C	0.27560800	-1.62374200	1.67246000
H	0.79166500	-2.58672200	1.63757000
H	0.88773000	-0.93756500	2.26181900
H	-0.63211800	-1.79273500	2.24575100
C	1.30651400	-0.88831400	-0.58313600
C	-0.00516200	1.38118900	0.87858900
H	-0.55740600	2.32273700	0.84865200
H	0.21749400	1.19365100	1.93511400

C	2.12571300	0.26935800	0.07054000
C	1.31308100	1.57058100	0.11864100
H	1.89132300	2.36907200	0.59582600
H	1.10928300	1.91393000	-0.90416000
C	1.01394000	-0.58436500	-2.07766400
H	1.86707000	-0.12292800	-2.58484300
H	0.77495200	-1.49811600	-2.62831500
H	0.17900700	0.10045300	-2.21734100
H	2.31698300	-0.03257800	1.10611600
C	3.53653200	0.52981300	-0.54802100
H	3.40819300	1.12812300	-1.46585200
C	2.19971700	-2.15282900	-0.52176400
H	2.47528100	-2.37861900	0.51042300
H	1.67173300	-3.02655300	-0.91058100
C	3.48646400	-1.97811600	-1.33008900
H	3.25616100	-1.94342500	-2.40766100
C	4.19828800	-0.69787500	-1.06658600
H	4.17987600	-2.81913900	-1.20863600
C	5.20807800	-0.80850000	1.37830500
H	4.38830700	-0.92843300	2.08016800
C	4.44706700	1.39713100	0.41820000
H	3.78290300	1.62696900	1.25809800
C	5.62235100	0.58845500	1.02707300
H	6.47781800	0.59100900	0.34468100
H	5.95495200	1.10985100	1.93080600
O	5.36318700	-0.52076100	-1.61998300
C	5.80081800	-1.94841800	0.96859900
O	6.85955700	-2.06017700	0.13700800
H	5.42685300	-2.92630000	1.25545500
C	4.86456400	2.76914500	-0.17442400
H	3.93050900	3.23811700	-0.51251500
C	5.80019000	2.69096800	-1.38936900
H	6.78057400	2.27869800	-1.12749700
H	5.38900200	2.08480300	-2.20231200
H	5.97287600	3.69432300	-1.78832300
C	5.45867100	3.68321000	0.90880500
H	5.59275800	4.69539500	0.51747900
H	4.80587100	3.75088400	1.78542200
H	6.44146000	3.33537800	1.24411700
C	-8.78018300	1.19498900	-0.52840100
H	-9.32501000	1.91864900	-1.13298300
H	-9.12818700	0.18316500	-0.75571300
H	-8.97120300	1.37295500	0.53350200
H	7.33368100	-1.21838300	0.08688300
H	5.74387300	-1.36108400	-1.93385400

(Fig.11) input structure

1 1

C	-5.12453800	0.46620500	-0.25168100
C	-4.60888700	-0.95240200	0.08778600
C	-3.04478500	-0.88674100	-0.01643400
C	-2.31357000	0.22389500	0.81517200
C	-2.97440400	1.58319400	0.45513100
C	-4.50165900	1.57679300	0.58507600
H	-4.91897700	0.67150700	-1.30782300
H	-2.57671400	2.38245400	1.08892900
H	-2.71417600	1.84678600	-0.57933500
H	-4.81284900	1.46891700	1.62945000
H	-4.89740900	2.53540800	0.23809900
H	-2.87221100	-0.59078700	-1.06460300
C	-2.40559100	0.04389100	2.35149000
H	-2.46069800	-0.99849500	2.66831200
H	-1.54947300	0.49770900	2.85724600

H	-3.29269800	0.53584100	2.75299500
C	-0.82116100	0.26294500	0.28975200
H	-0.95395700	0.52169700	-0.76784300
C	-2.34045900	-2.24167200	0.14295800
H	-2.87080100	-3.01364200	-0.42020600
H	-2.35297700	-2.57489900	1.18582100
C	-0.90365100	-2.17734700	-0.39313300
H	-0.97025700	-1.98059600	-1.46784200
H	-0.43525700	-3.16285600	-0.28567700
C	-0.02596800	-1.09629300	0.28878100
C	-5.15193500	-1.90850500	-0.99878500
H	-4.97638700	-2.95785600	-0.74819200
H	-6.23234600	-1.77757800	-1.09704300
H	-4.69835800	-1.70979300	-1.97639700
C	-5.14093700	-1.44517300	1.44848100
H	-4.94912600	-0.75124400	2.26711400
H	-6.22234900	-1.58794900	1.39124300
H	-4.69402800	-2.40830600	1.71336100
O	-6.56172100	0.47732700	-0.08386700
C	-7.26081500	1.33598100	-0.86597300
O	-6.75046100	2.07542900	-1.67094900
C	0.28969100	-1.59487200	1.72078300
H	0.79647400	-2.56296200	1.69958400
H	0.90659300	-0.90774400	2.30404400
H	-0.62271100	-1.74613700	2.29170900
C	1.34236000	-0.89547000	-0.53555900
C	0.02687900	1.40346200	0.88064400
H	-0.52140000	2.34653300	0.83094300
H	0.24071600	1.23241700	1.94181900
C	2.15844200	0.26884900	0.11086100
C	1.35144200	1.57434300	0.12748200
H	1.92840900	2.38106700	0.59337900
H	1.15578700	1.89876600	-0.90319800
C	1.06215500	-0.60966600	-2.03626500
H	1.92389900	-0.16758400	-2.54599300
H	0.81837100	-1.52957500	-2.57399800
H	0.23539400	0.08151600	-2.19220900
H	2.33734400	-0.01546800	1.15298800
C	3.57388400	0.51184100	-0.50206800
H	3.43523800	1.09746200	-1.42769600
C	2.23261400	-2.16084200	-0.45401100
H	2.49935900	-2.37544800	0.58279200
H	1.70250000	-3.03682900	-0.83499700
C	3.52586600	-2.00437000	-1.25522900
H	3.31344000	-1.99369600	-2.33623600
C	4.23757700	-0.72399900	-1.01121100
H	4.22691700	-2.83291300	-1.11885400
C	5.12604900	-0.80517000	1.58460700
H	4.49429900	-0.88545300	2.46291200
C	4.48954800	1.38320100	0.45193000
H	3.80495600	1.70105700	1.24365300
C	5.59574100	0.55348800	1.15775400
H	6.49394900	0.44625200	0.54272500
H	5.91630400	1.11961600	2.03924500
O	5.40481400	-0.66606000	-1.56815800
C	5.54774200	-1.96431600	1.04376600
O	5.97038300	-1.93854600	-0.29733600
H	5.21960300	-2.92512000	1.43355800
C	5.00799300	2.69506700	-0.19902600
H	4.11640600	3.22161800	-0.56438900
C	5.93239800	2.48639500	-1.40948800
H	6.85170300	1.94497100	-1.14555100
H	5.43295500	1.97992400	-2.24701700

H	6.26393600	3.44824000	-1.81022700
C	5.68116600	3.60618800	0.83895500
H	5.88745900	4.58860500	0.40535400
H	5.04057300	3.75781200	1.71353900
H	6.63532400	3.19673900	1.18583700
C	-8.73844000	1.22839300	-0.58761500
H	-9.27654600	1.94589100	-1.20542300
H	-9.08821500	0.21482700	-0.80419700
H	-8.93641800	1.42079600	0.47049500
H	5.40598100	-1.53572000	-0.96700400
H	5.35984800	-0.46474200	-2.52231400

(Fig.11) output structure

1 1

C	-5.16895600	0.42213800	-0.29251900
C	-4.65388100	-0.98108000	0.10507800
C	-3.08769700	-0.91209100	0.02659900
C	-2.37560300	0.23166200	0.82939100
C	-3.03771100	1.57423600	0.41287500
C	-4.56684100	1.56257200	0.51823700
H	-4.94442500	0.59162000	-1.35096000
H	-2.65471500	2.39649200	1.02580000
H	-2.76289800	1.80563100	-0.62537600
H	-4.89428000	1.48456200	1.56031400
H	-4.96277700	2.50742900	0.13530200
H	-2.89755000	-0.65355800	-1.02832500
C	-2.49366800	0.10520600	2.36948900
H	-2.53515400	-0.92621200	2.72223800
H	-1.65533600	0.59328500	2.87295100
H	-3.39705000	0.59425800	2.73664600
C	-0.87392000	0.25947600	0.32879600
H	-0.98866300	0.48331300	-0.73853200
C	-2.38051200	-2.25656300	0.24988300
H	-2.89947900	-3.05254000	-0.29001000
H	-2.40837200	-2.54658000	1.30525200
C	-0.93535800	-2.20580600	-0.26524900
H	-0.98609800	-2.05038100	-1.34745100
H	-0.46333200	-3.18388400	-0.11347600
C	-0.07280200	-1.09542900	0.38791800
C	-5.17323300	-1.97595700	-0.95821700
H	-4.99299300	-3.01544000	-0.67212400
H	-6.25302400	-1.85711700	-1.07726200
H	-4.70598600	-1.80401600	-1.93449400
C	-5.20955400	-1.42956400	1.47201800
H	-5.03291500	-0.70939800	2.27138300
H	-6.28973700	-1.57612000	1.40094800
H	-4.76644800	-2.38273700	1.77596500
O	-6.60933700	0.43236500	-0.15201800
C	-7.29803700	1.25331800	-0.98179300
O	-6.77757400	1.96062800	-1.80892800
C	0.22011800	-1.53482700	1.84365600
H	0.72557200	-2.50363300	1.87151000
H	0.82882700	-0.82341400	2.40621000
H	-0.70087200	-1.66252800	2.40617400
C	1.30689300	-0.91609600	-0.42324900
C	-0.04017200	1.41981600	0.89997000
H	-0.59337500	2.35814600	0.82001300
H	0.16021200	1.27593500	1.96775200
C	2.11083700	0.27984300	0.17897800
C	1.29292100	1.57848200	0.15995600
H	1.86190800	2.39644100	0.61543100
H	1.10925500	1.87906100	-0.87988900
C	1.04375200	-0.69864700	-1.93774500

H	1.90305000	-0.25330200	-2.44669200
H	0.83225400	-1.64501000	-2.44277600
H	0.20239900	-0.03532300	-2.13412600
H	2.28673200	0.03042100	1.23423600
C	3.52396100	0.50740600	-0.44227900
H	3.40539100	0.96220100	-1.43065000
C	2.20424100	-2.17126700	-0.27366800
H	2.41636600	-2.36029900	0.78310300
H	1.69214800	-3.06199300	-0.64703100
C	3.53263900	-2.03514600	-1.02718100
H	3.32954700	-1.96309100	-2.10246300
C	4.32883100	-0.79912600	-0.61587600
H	4.14288200	-2.93754300	-0.89750900
C	5.12633600	-0.91438800	0.70703000
H	4.47301900	-1.24075800	1.51960700
C	4.39705100	1.42661800	0.47845700
H	3.79317900	1.66126300	1.36066000
C	5.59173300	0.56664400	0.98443300
H	6.50004800	0.77706800	0.41516400
H	5.81362000	0.72156600	2.04087400
O	5.40670800	-0.58602300	-1.62032900
C	6.31677100	-1.77564100	0.65630800
O	6.95811100	-1.97197000	-0.40346500
H	6.73193800	-2.24179900	1.55450100
C	4.82011400	2.79292400	-0.12106300
H	3.88007000	3.27702000	-0.41810800
C	5.70475300	2.71360600	-1.37283100
H	6.69285900	2.29226500	-1.15693400
H	5.25403100	2.11716200	-2.17038800
H	5.87011200	3.71904200	-1.77004300
C	5.47391300	3.67957400	0.94947800
H	5.66384900	4.68188100	0.55579400
H	4.83494400	3.78438200	1.83242000
H	6.43823200	3.27449000	1.27785300
C	-8.77998900	1.15003500	-0.72465500
H	-9.31017300	1.83998300	-1.37959900
H	-9.12186400	0.12708500	-0.90713800
H	-8.99704500	1.38302400	0.32147000
H	6.42094500	-1.44042200	-1.17331000
H	5.04633100	-0.69948500	-2.51033500

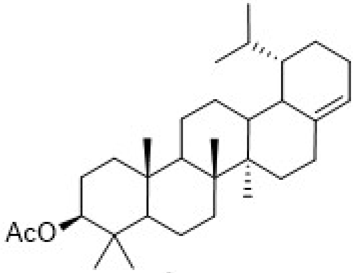

2

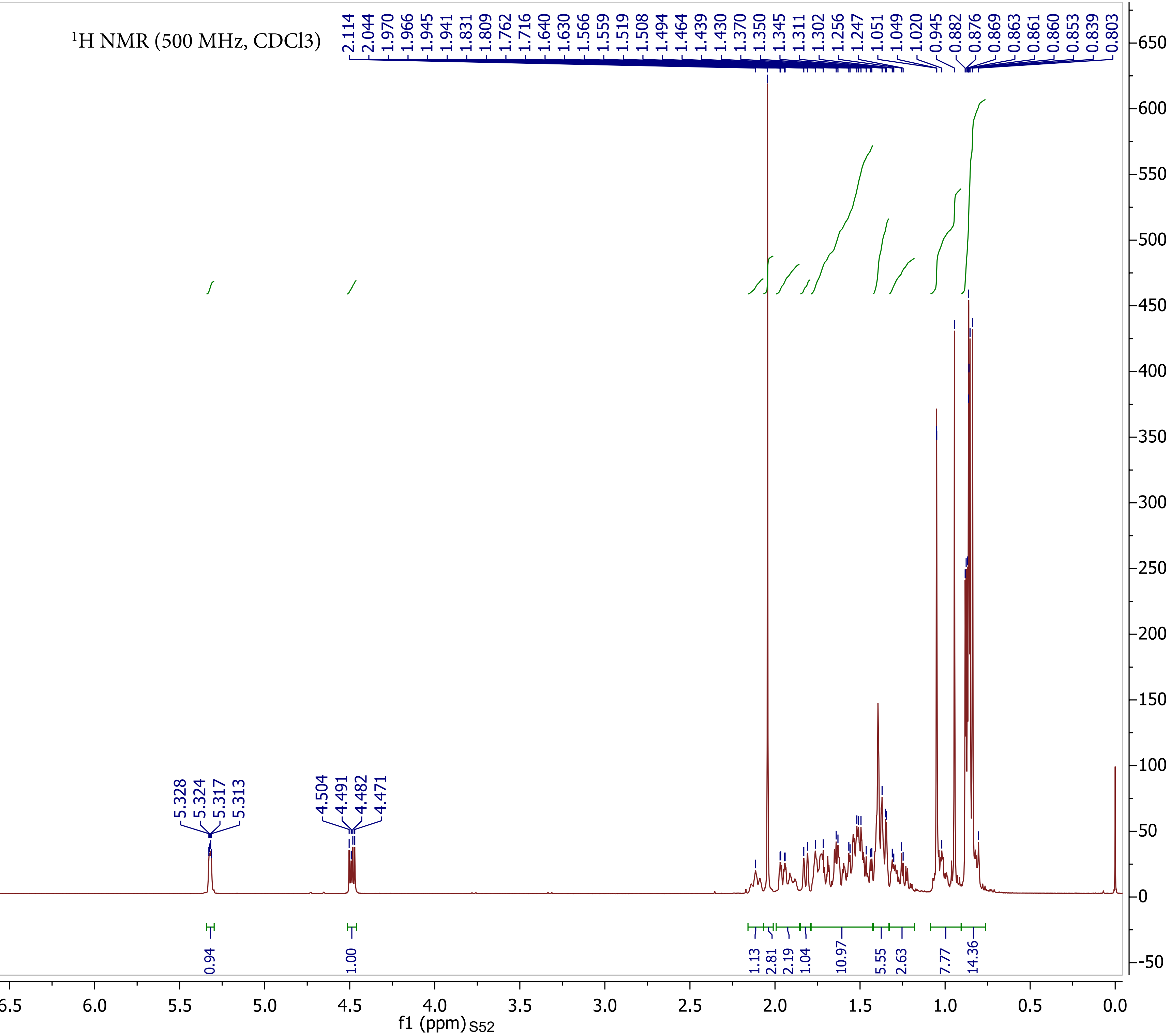

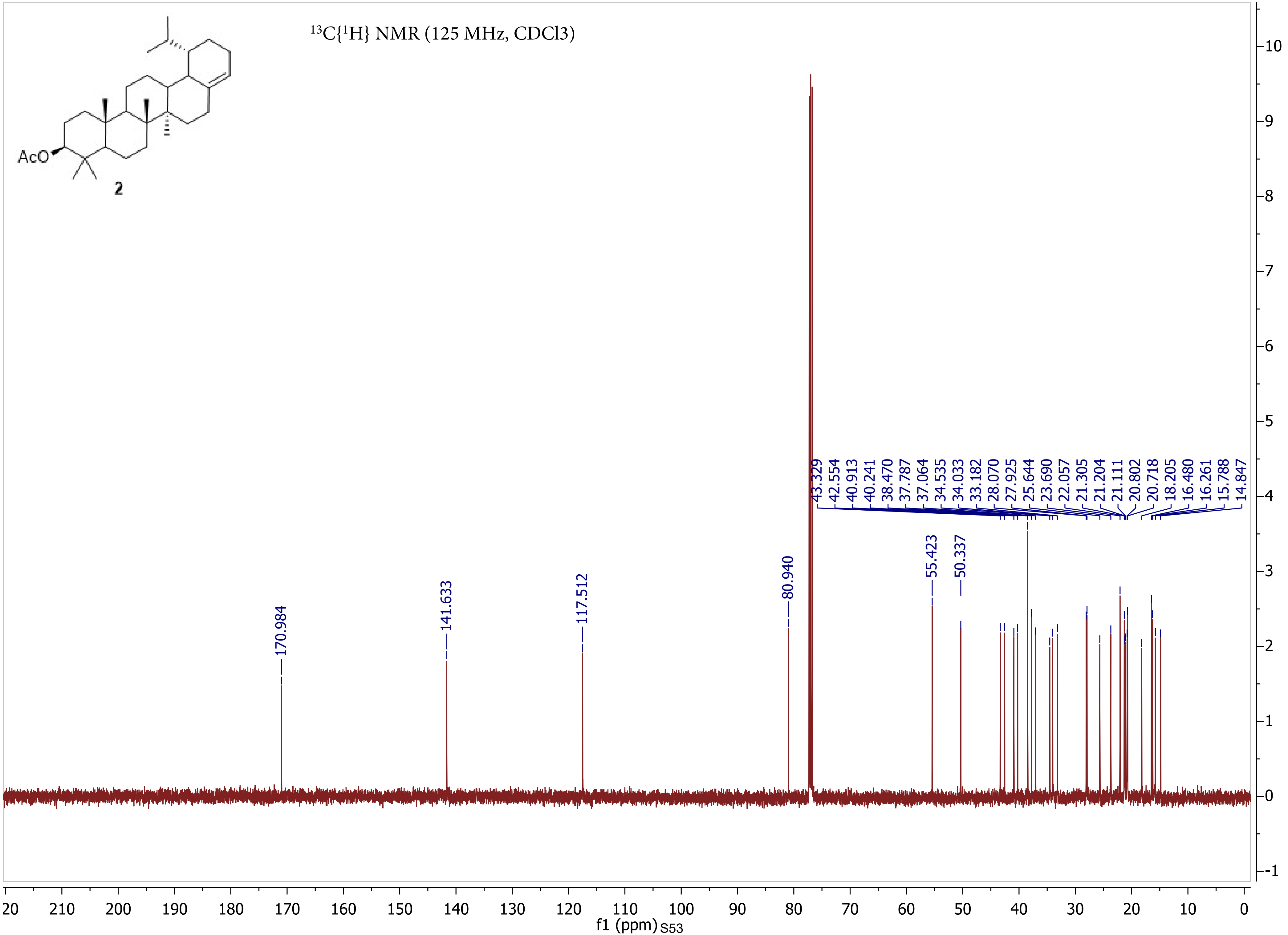
13C{1H} NMR (125 MHz, CDCl3)
AcO
2
170.984
141.633
117.512
80.940
55.423
50.337
43.329
42.554
40.913
40.241
38.470
37.787
37.064
34.535
34.033
33.182
28.070
27.925
25.644
23.690
22.057
21.305
21.204
21.111
20.802
20.718
18.205
16.480
16.261
15.788
14.847
f1 (ppm)

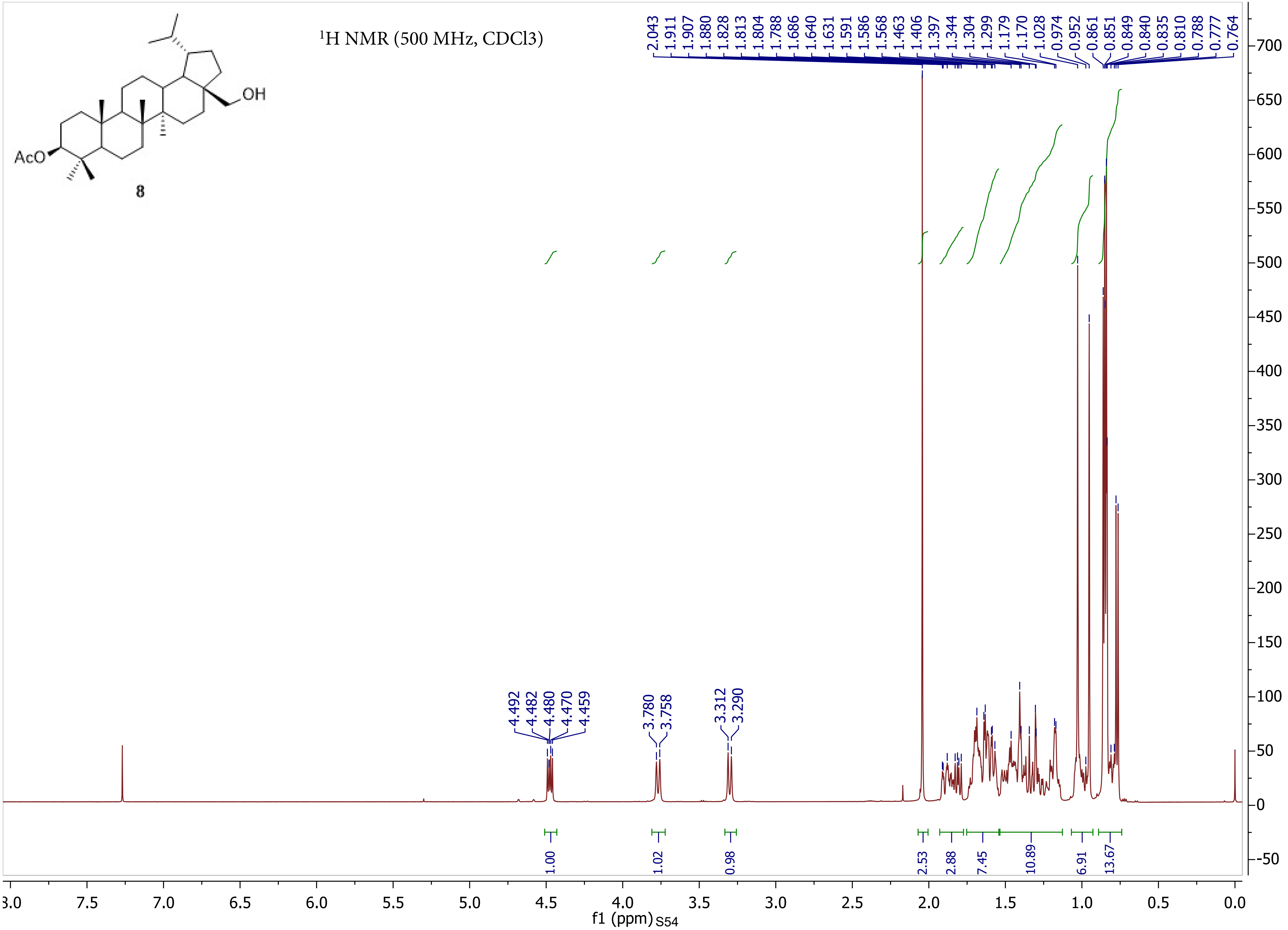

^{1}H NMR (500 MHz, CDCl3)
AcO
OH
8
2.043 1.911 1.907 1.880 1.828 1.813 1.804 1.788 1.686 1.640 1.631 1.591 1.586 1.568 1.463 1.406 1.397 1.344 1.304 1.299 1.179 1.170 1.028 0.974 0.952 0.861 0.851 0.849 0.840 0.835 0.810 0.788 0.777 0.764
4.492 4.482 4.480 4.470 4.459
3.780 3.758
3.312 3.290
1.00 1.02 0.98 2.53 2.88 7.45 10.89 6.91 13.67
f1 (ppm)

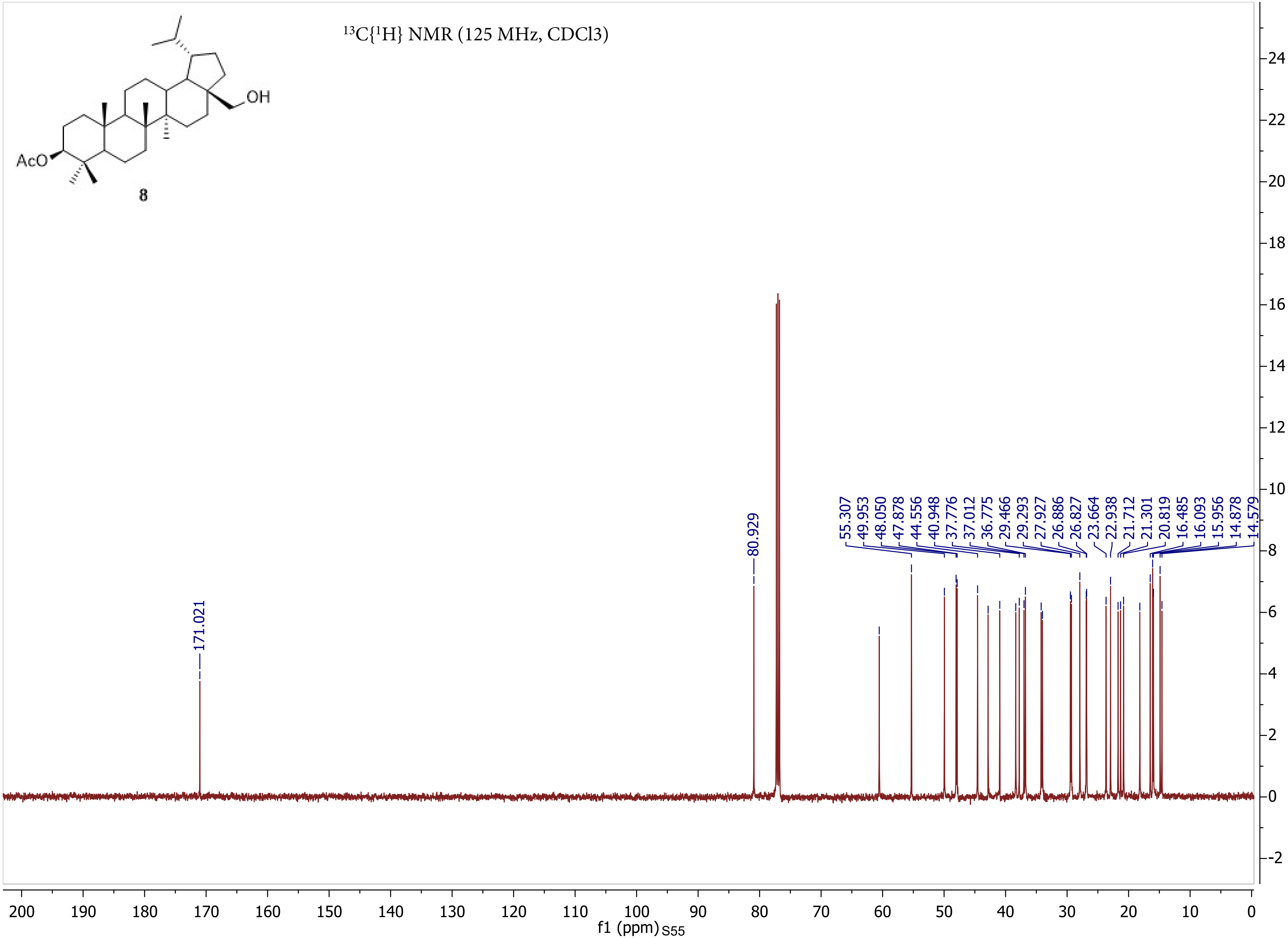
13C{1H} NMR (125 MHz, CDCl3)
AcO
OH
8
171.021
80.929
55.307
49.953
48.050
47.878
44.556
40.948
37.776
37.012
36.775
29.466
29.293
27.927
26.886
26.827
23.664
22.938
21.712
21.301
20.819
16.485
16.093
15.956
14.878
14.579
f1 (ppm)

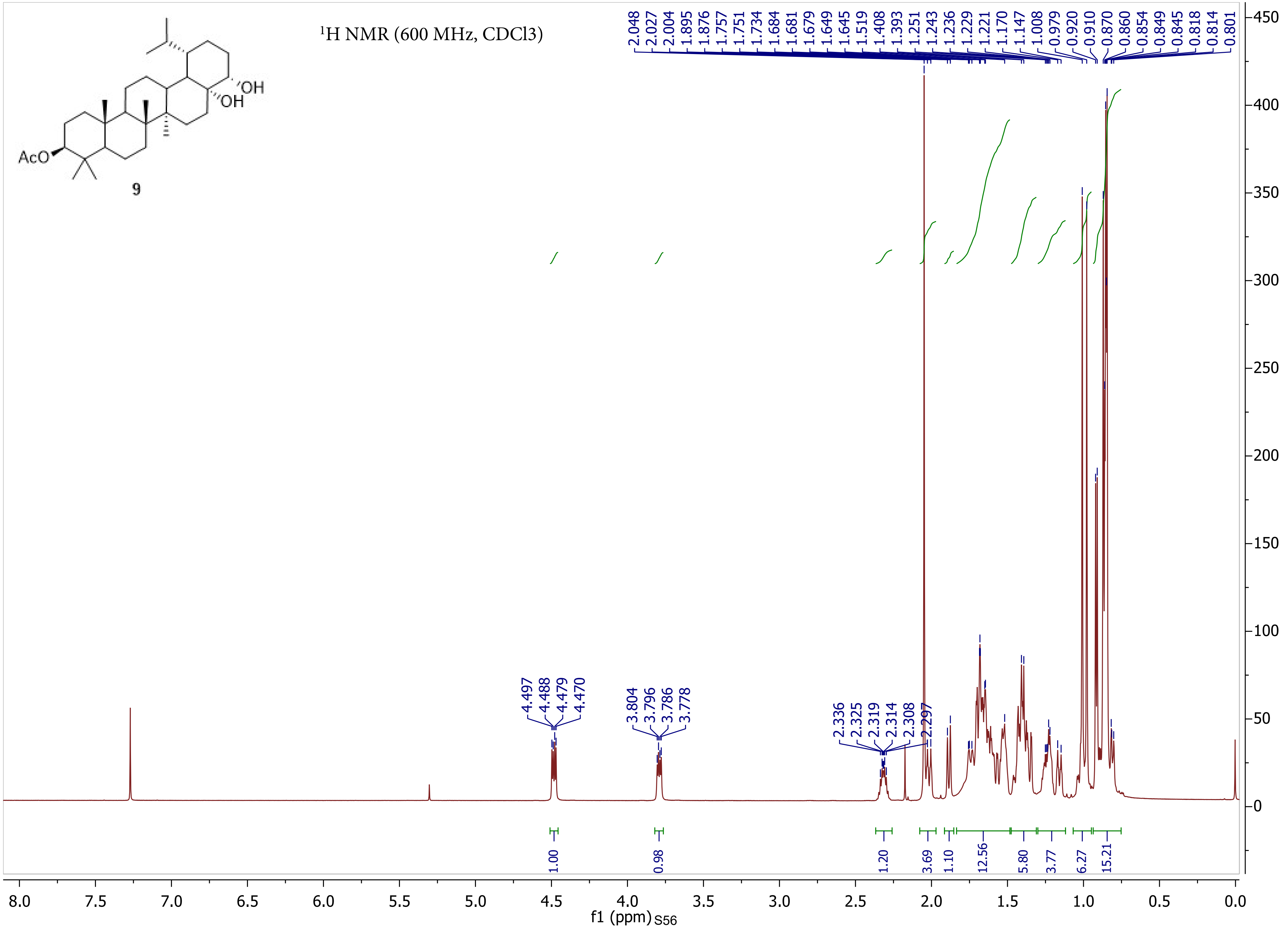

¹H NMR (600 MHz, CDCl3)
AcO
OH
OH
9
f1 (ppm)

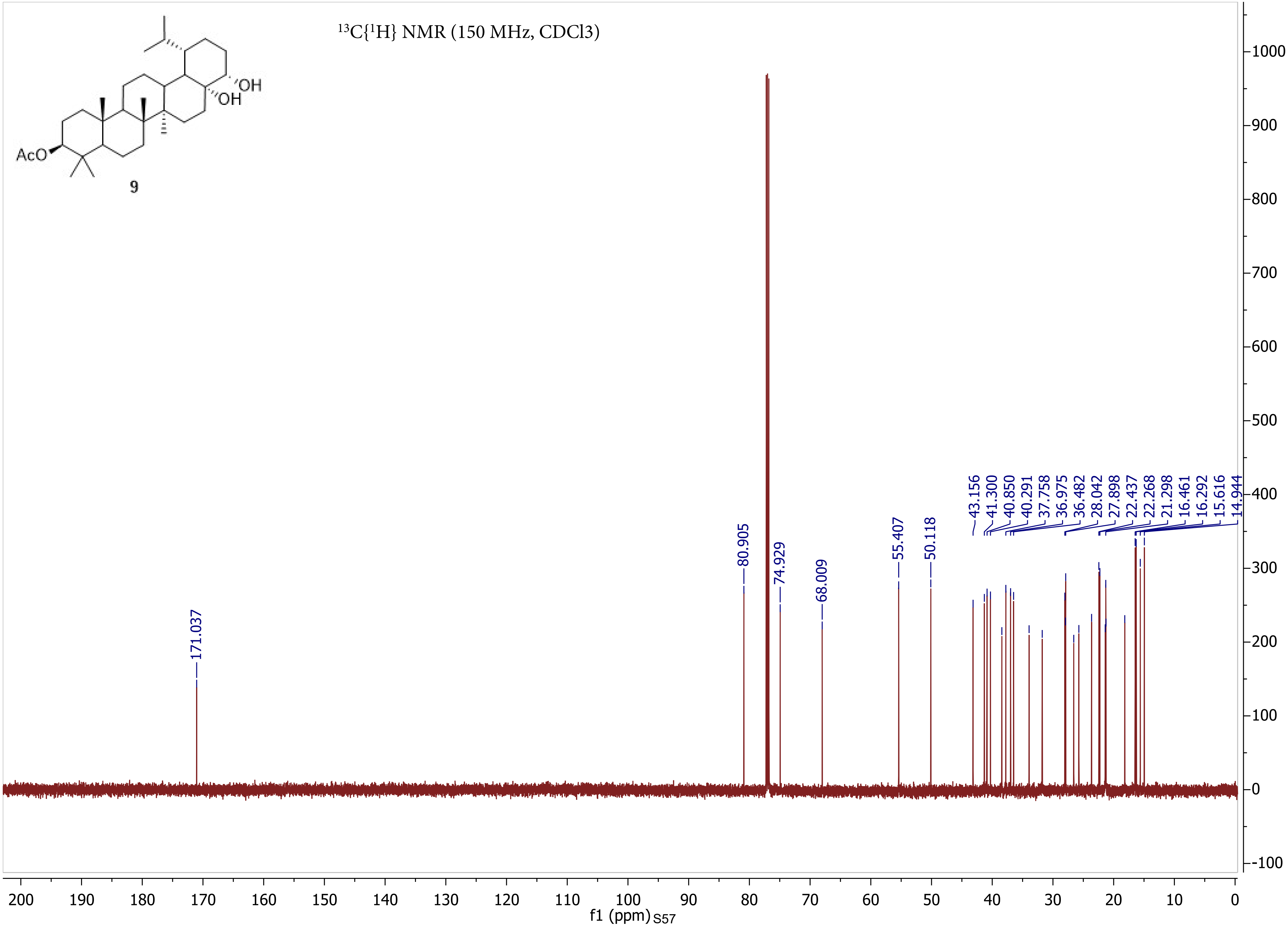

¹³C{¹H} NMR (150 MHz, CDCl3)
9
171.037
80.905
74.929
68.009
55.407
50.118
43.156
41.300
40.850
40.291
37.758
36.975
36.482
28.042
27.898
22.437
22.268
21.298
16.461
16.292
15.616
14.944
f1 (ppm)

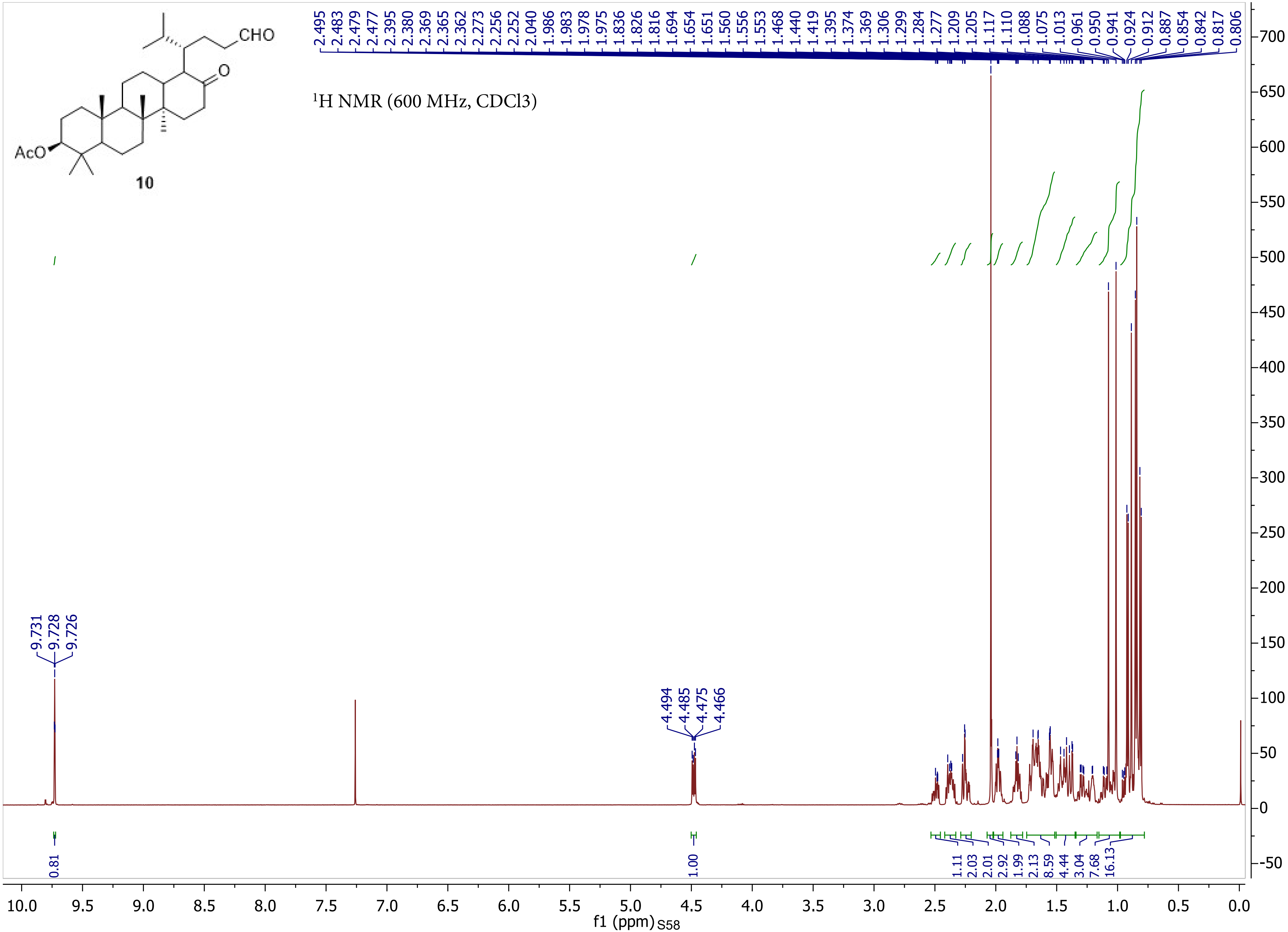
CHO
O
AcO
10
1H NMR (600 MHz, CDCl3)
2.495 2.483 2.479 2.477 2.395 2.380 2.369 2.365 2.362 2.273 2.256 2.252 2.040 1.986 1.983 1.978 1.975 1.836 1.826 1.816 1.694 1.654 1.651 1.560 1.556 1.553 1.468 1.440 1.419 1.395 1.374 1.369 1.306 1.299 1.284 1.277 1.209 1.205 1.117 1.110 1.088 1.075 1.013 0.961 0.950 0.941 0.924 0.912 0.887 0.854 0.842 0.817 0.806
9.731 9.728 9.726
4.494 4.485 4.475 4.466
0.81
1.00
1.11 2.03 2.01 2.92 1.99 2.13 8.59 4.44 3.04 7.68 16.13
f1 (ppm)

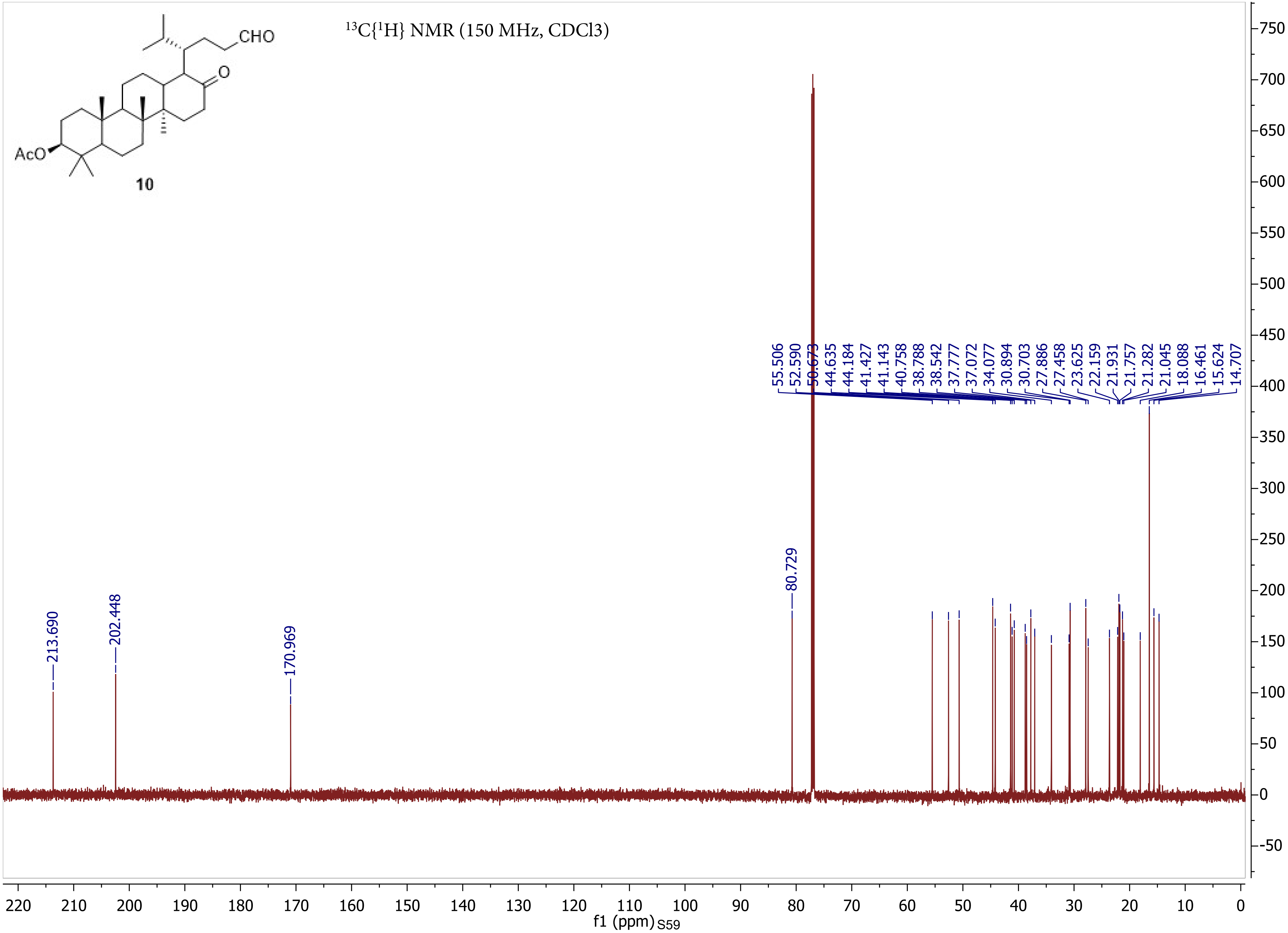

CHO
O
AcO
10
^{13}C{^{1}H} NMR (150 MHz, CDCl3)
55.506
52.590
50.673
44.635
44.184
41.427
41.143
40.758
38.788
38.542
37.777
37.072
34.077
30.894
30.703
27.886
27.458
23.625
22.159
21.931
21.757
21.282
21.045
18.088
16.461
15.624
14.707
80.729
213.690
202.448
170.969
f1 (ppm)

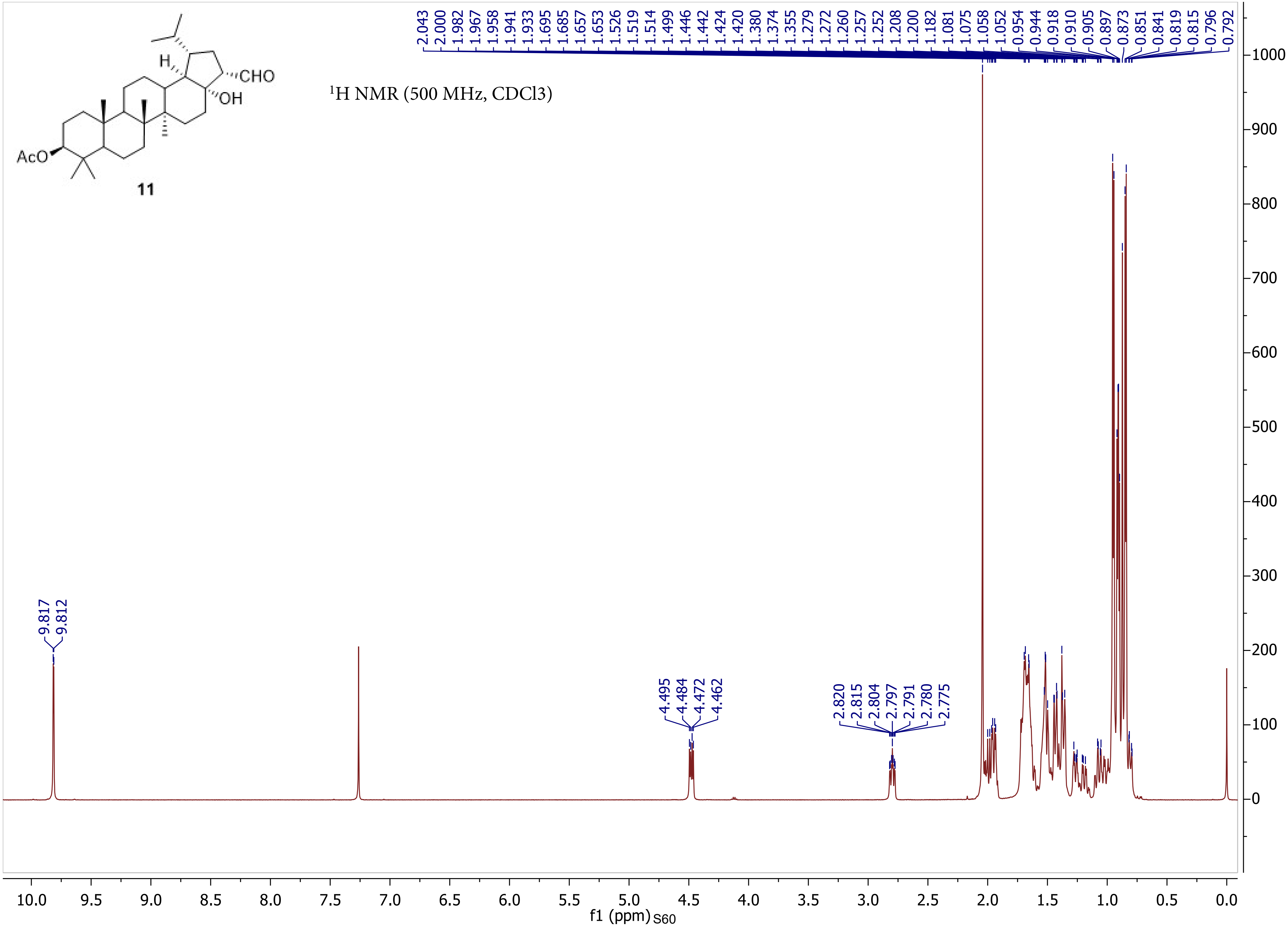
AcO
OH
CHO
H
11
1H NMR (500 MHz, CDCl3)
2.043 2.000 1.982 1.967 1.958 1.941 1.933 1.695 1.685 1.657 1.653 1.526 1.519 1.514 1.499 1.446 1.442 1.424 1.420 1.380 1.374 1.355 1.279 1.272 1.260 1.257 1.252 1.208 1.200 1.182 1.081 1.075 1.058 1.052 0.954 0.944 0.918 0.910 0.905 0.897 0.873 0.851 0.841 0.819 0.815 0.796 0.792
9.817
9.812
4.495
4.484
4.472
4.462
2.820
2.815
2.804
2.797
2.791
2.780
2.775
f1 (ppm)

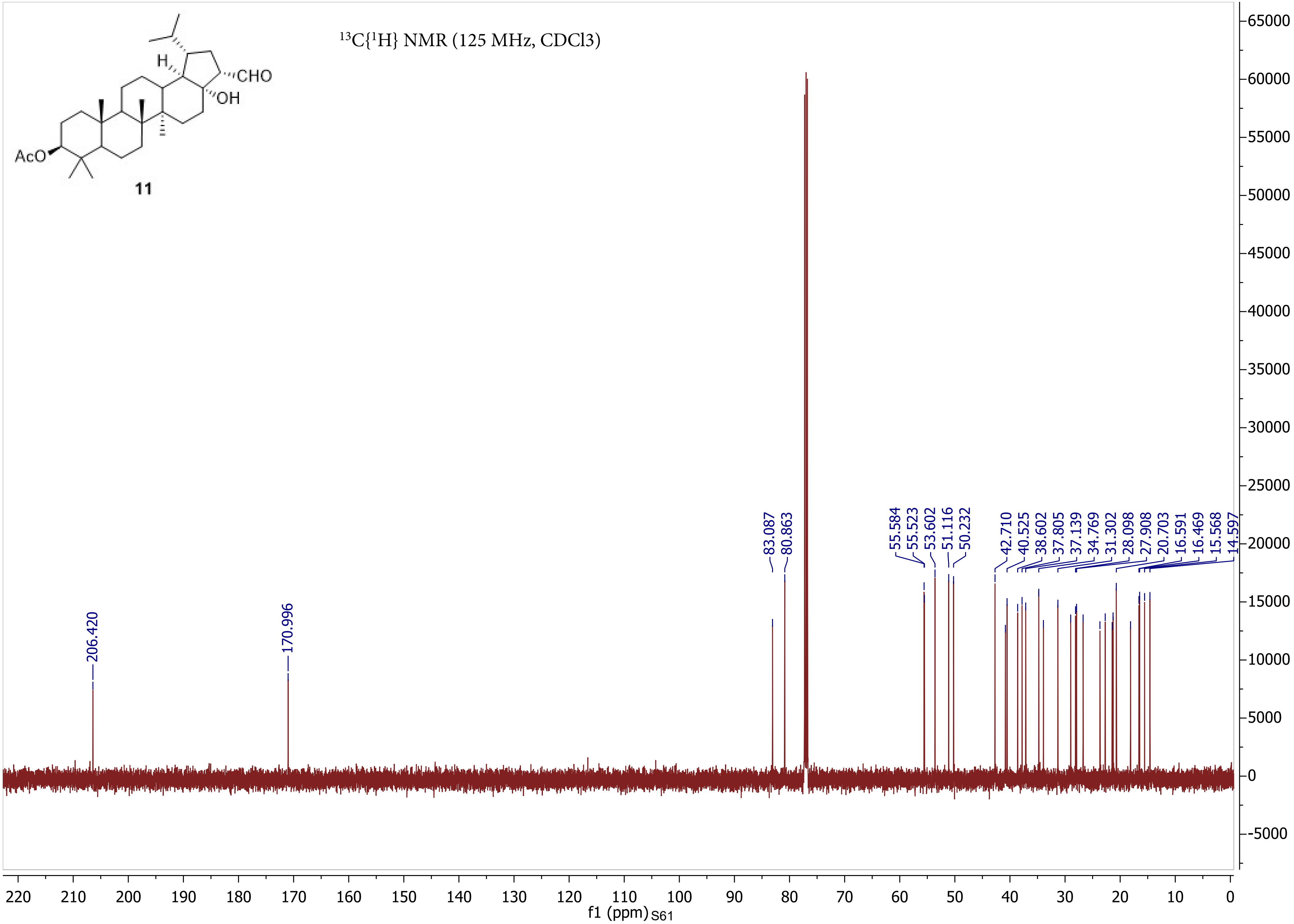

AcO
H
CHO
OH
11
^{13}C{^{1}H} NMR (125 MHz, CDCl3)
206.420
170.996
83.087
80.863
55.584
55.523
53.602
51.116
50.232
42.710
40.525
38.602
37.805
37.139
34.769
31.302
28.098
27.908
20.703
16.591
16.469
15.568
14.597
f1 (ppm)

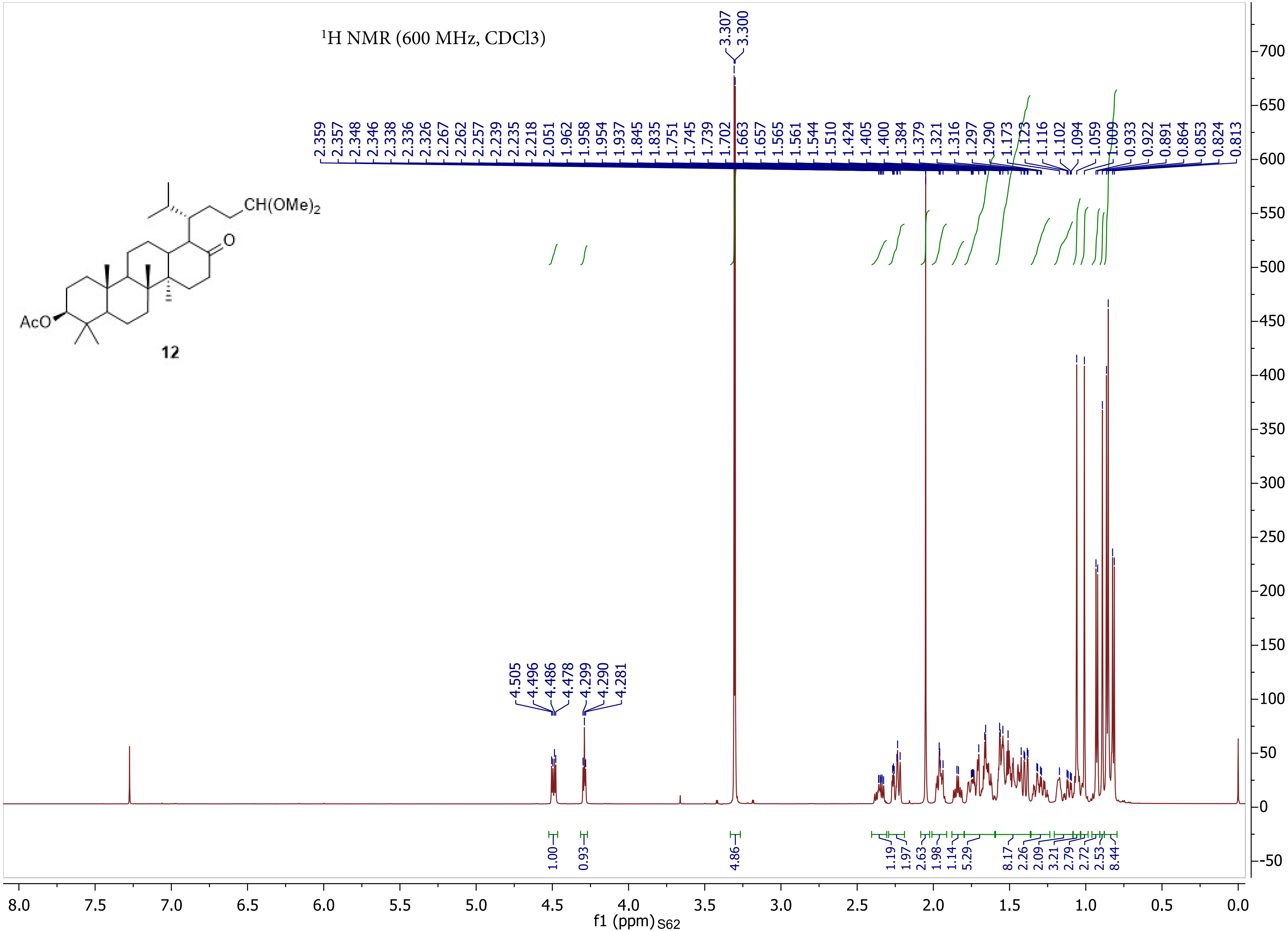

1H NMR (600 MHz, CDCl3)
CH(OMe)2
O
AcO
12
3.307
3.300
2.359 2.357 2.348 2.346 2.338 2.336 2.326 2.267 2.262 2.257 2.239 2.235 2.218 2.051 1.962 1.958 1.954 1.937 1.845 1.835 1.751 1.745 1.739 1.702 1.663 1.657 1.565 1.561 1.544 1.510 1.424 1.405 1.400 1.384 1.379 1.321 1.316 1.297 1.290 1.173 1.123 1.116 1.102 1.094 1.059 1.009 0.933 0.922 0.891 0.864 0.853 0.824 0.813
4.505 4.496 4.486 4.478 4.299 4.290 4.281
1.00 0.93 4.86 1.19 1.97 2.63 1.98 1.14 5.29 8.17 2.26 2.09 3.21 2.79 2.72 2.53 8.44
f1 (ppm)

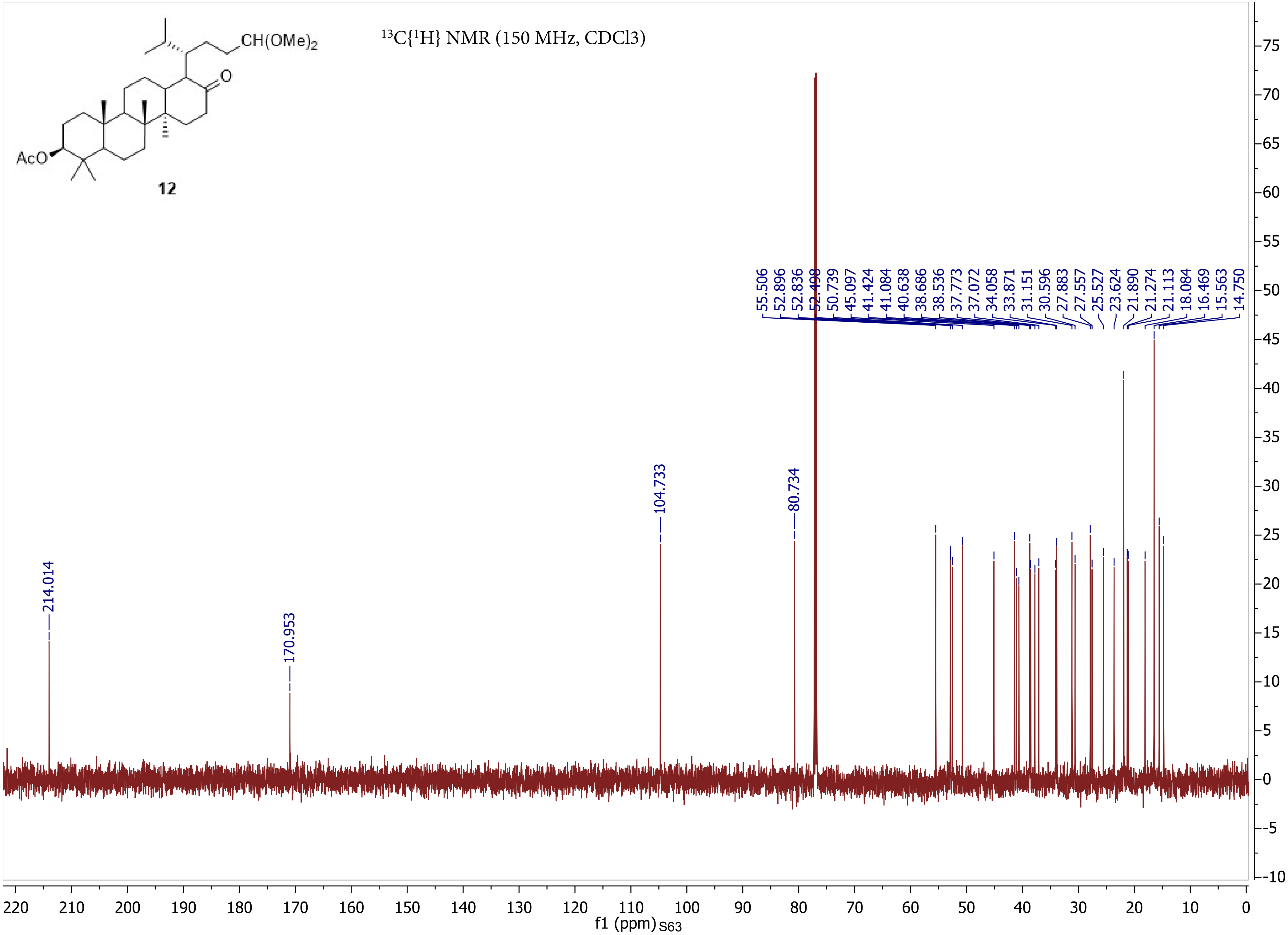

CH(OMe)2
O
AcO
12
13C{1H} NMR (150 MHz, CDCl3)
214.014
170.953
104.733
80.734
55.506
52.896
52.836
52.498
50.739
45.097
41.424
41.084
40.638
38.686
38.536
37.773
37.072
34.058
33.871
31.151
30.596
27.883
27.557
25.527
23.624
21.890
21.274
21.113
18.084
16.469
15.563
14.750
f1 (ppm)

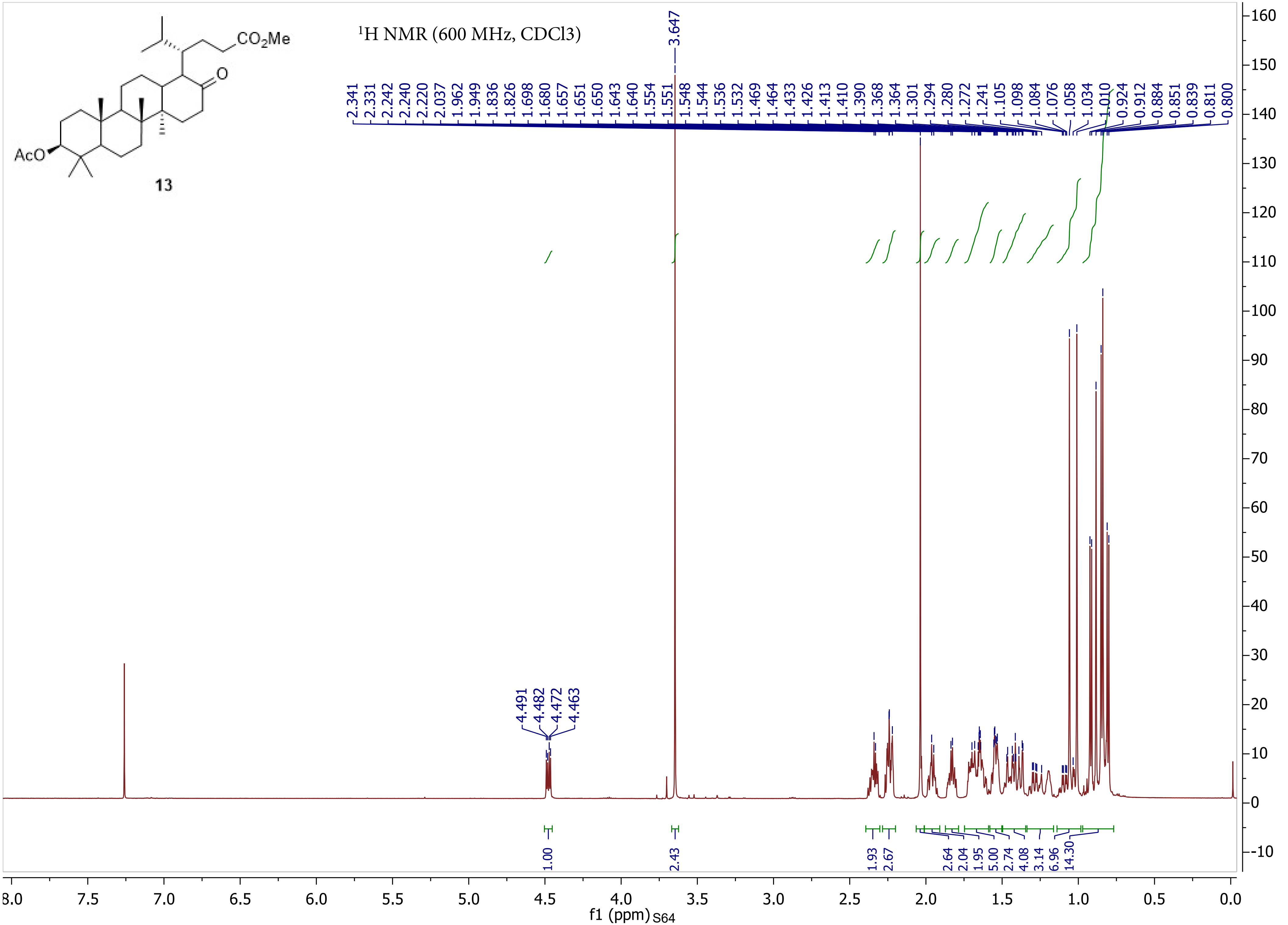

CO2Me
O
AcO
13
1H NMR (600 MHz, CDCl3)
3.647
2.341 2.331 2.242 2.240 2.220 2.037 1.962 1.949 1.836 1.826 1.698 1.680 1.657 1.651 1.650 1.643 1.640 1.554 1.551 1.548 1.544 1.536 1.532 1.469 1.464 1.433 1.426 1.413 1.410 1.390 1.368 1.364 1.301 1.294 1.280 1.272 1.241 1.105 1.098 1.084 1.076 1.058 1.034 1.010 0.924 0.912 0.884 0.851 0.839 0.811 0.800
4.491 4.482 4.472 4.463
1.00 2.43 1.93 2.67 2.64 2.04 1.95 5.00 2.74 4.08 3.14 6.96 14.30
f1 (ppm)

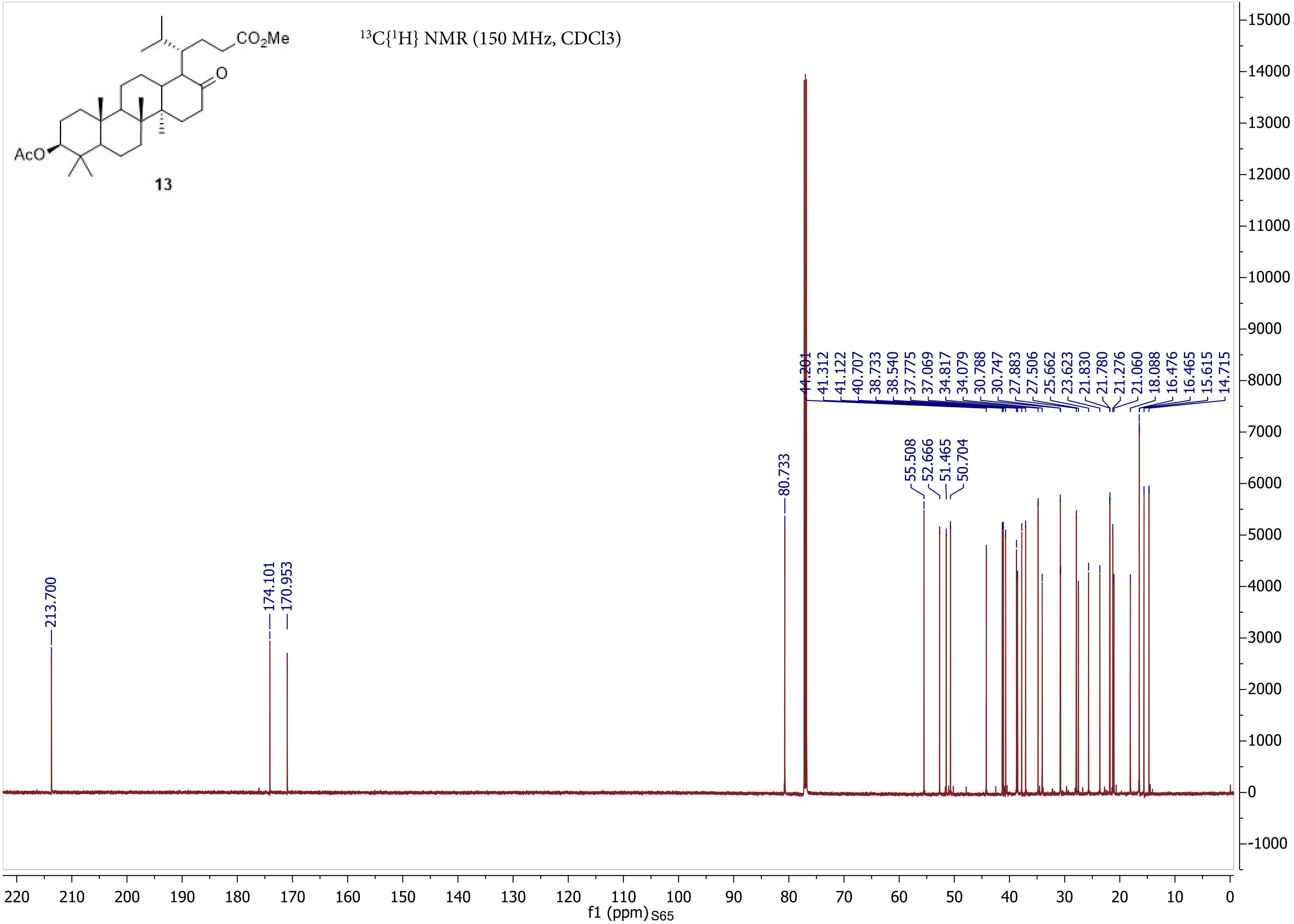
CO2Me
O
AcO
13
13C{1H} NMR (150 MHz, CDCl3)
213.700
174.101
170.953
80.733
55.508
52.666
51.465
50.704
44.201
41.312
41.122
40.707
38.733
38.540
37.775
37.069
34.817
34.079
30.788
30.747
27.883
27.506
25.662
23.623
21.830
21.780
21.276
21.060
18.088
16.476
16.465
15.615
14.715
f1 (ppm)

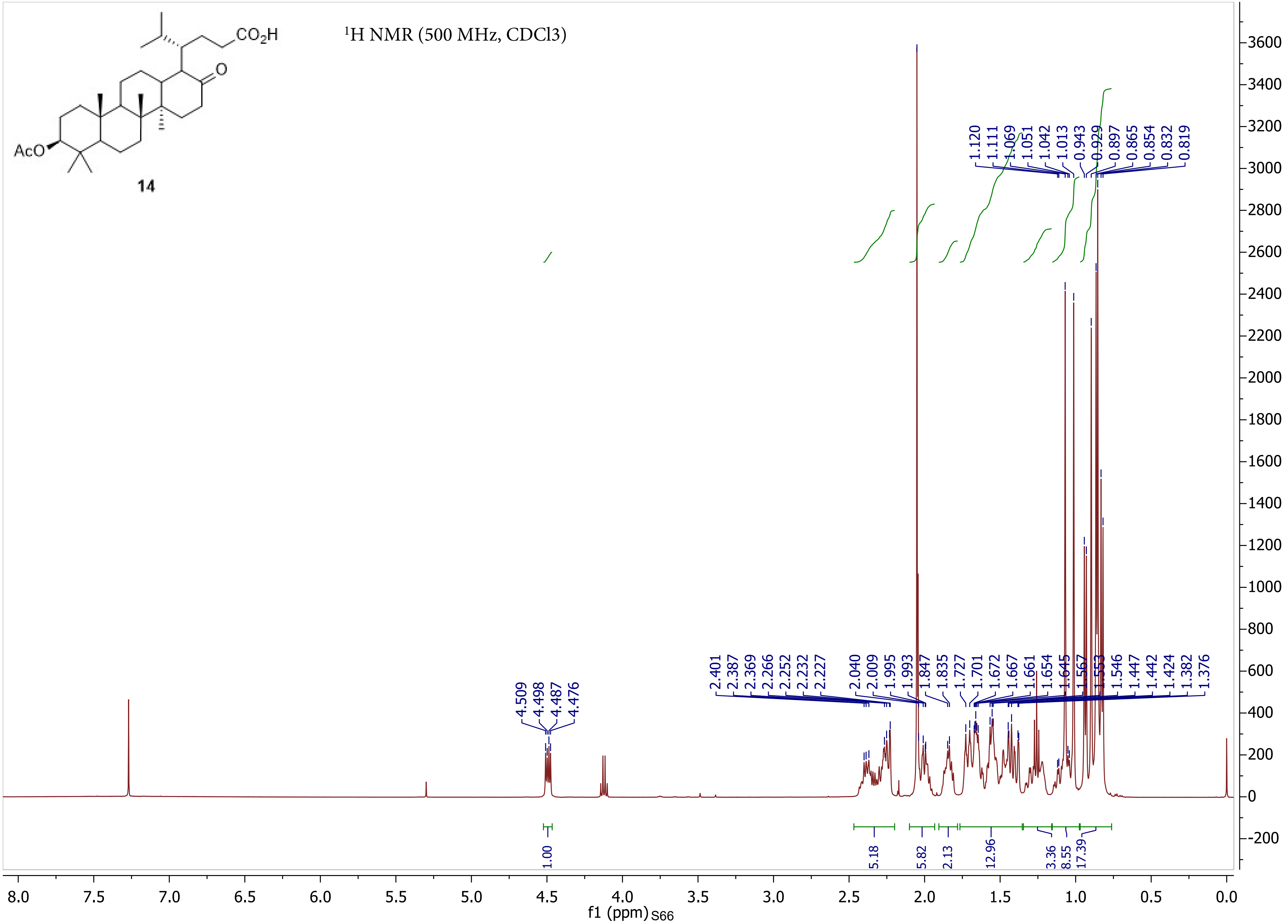

^{1}H NMR (500 MHz, CDCl3)
CO_2H
O
AcO
14
1.120 1.111 1.069 1.051 1.042 1.013 0.943 0.929 0.897 0.865 0.854 0.832 0.819
4.509 4.498 4.487 4.476
2.401 2.387 2.369 2.266 2.252 2.232 2.227 2.040 2.009 1.995 1.993 1.847 1.835 1.727 1.701 1.672 1.667 1.661 1.654 1.645 1.567 1.553 1.546 1.447 1.442 1.424 1.382 1.376
1.00 5.18 5.82 2.13 12.96 3.36 8.55 17.39
f1 (ppm)

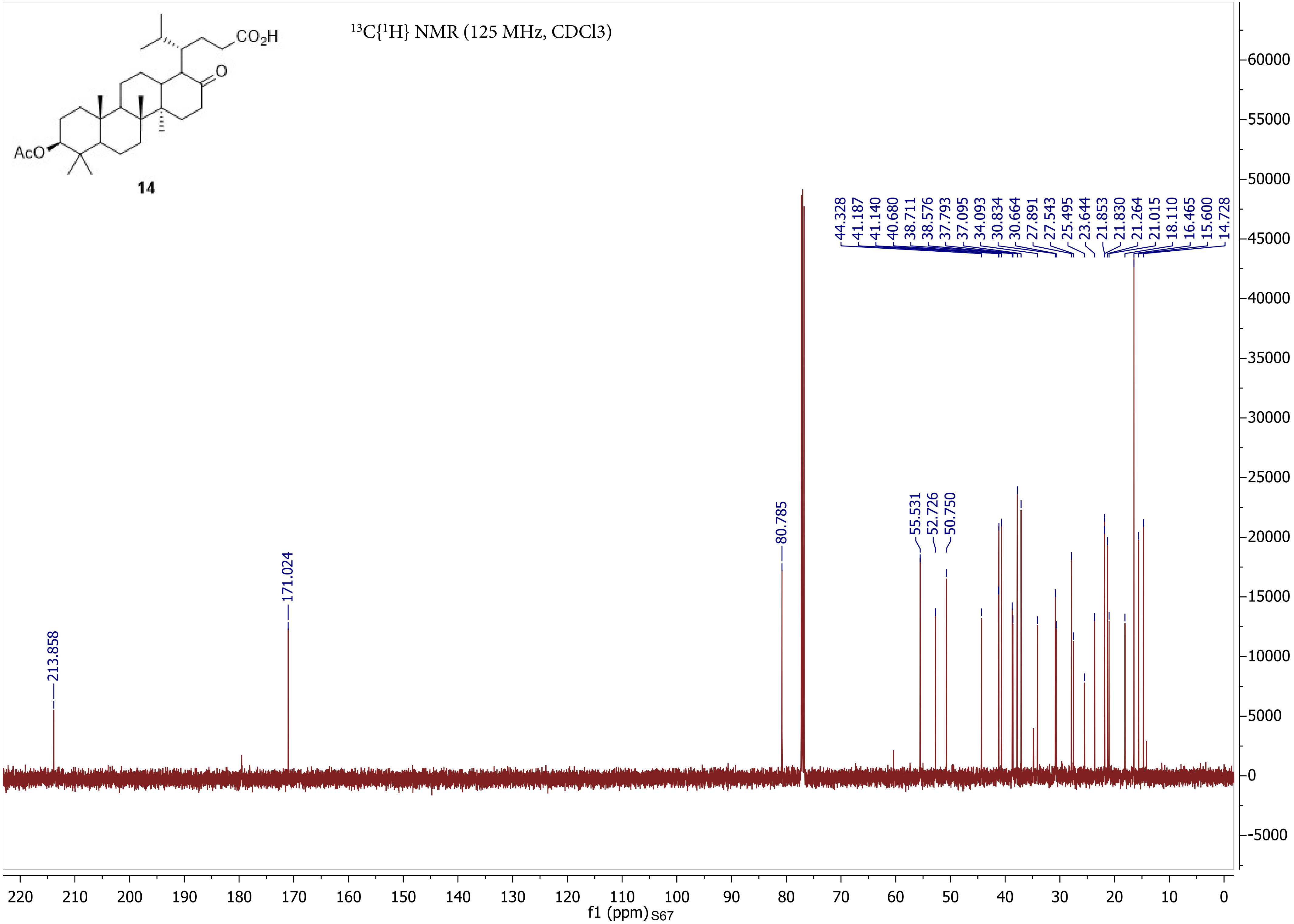

AcO
CO_2H
O
14
$^{13}C\{^1H\}$ NMR (125 MHz, CDCl3)
213.858
171.024
80.785
55.531
52.726
50.750
44.328
41.187
41.140
40.680
38.711
38.576
37.793
37.095
34.093
30.834
30.664
27.891
27.543
25.495
23.644
21.853
21.830
21.264
21.015
18.110
16.465
15.600
14.728
f1 (ppm)

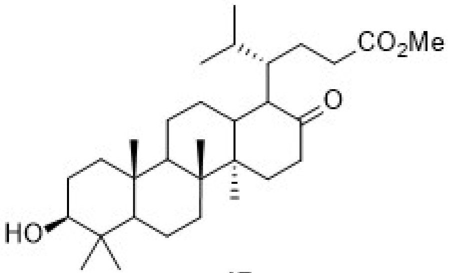

17

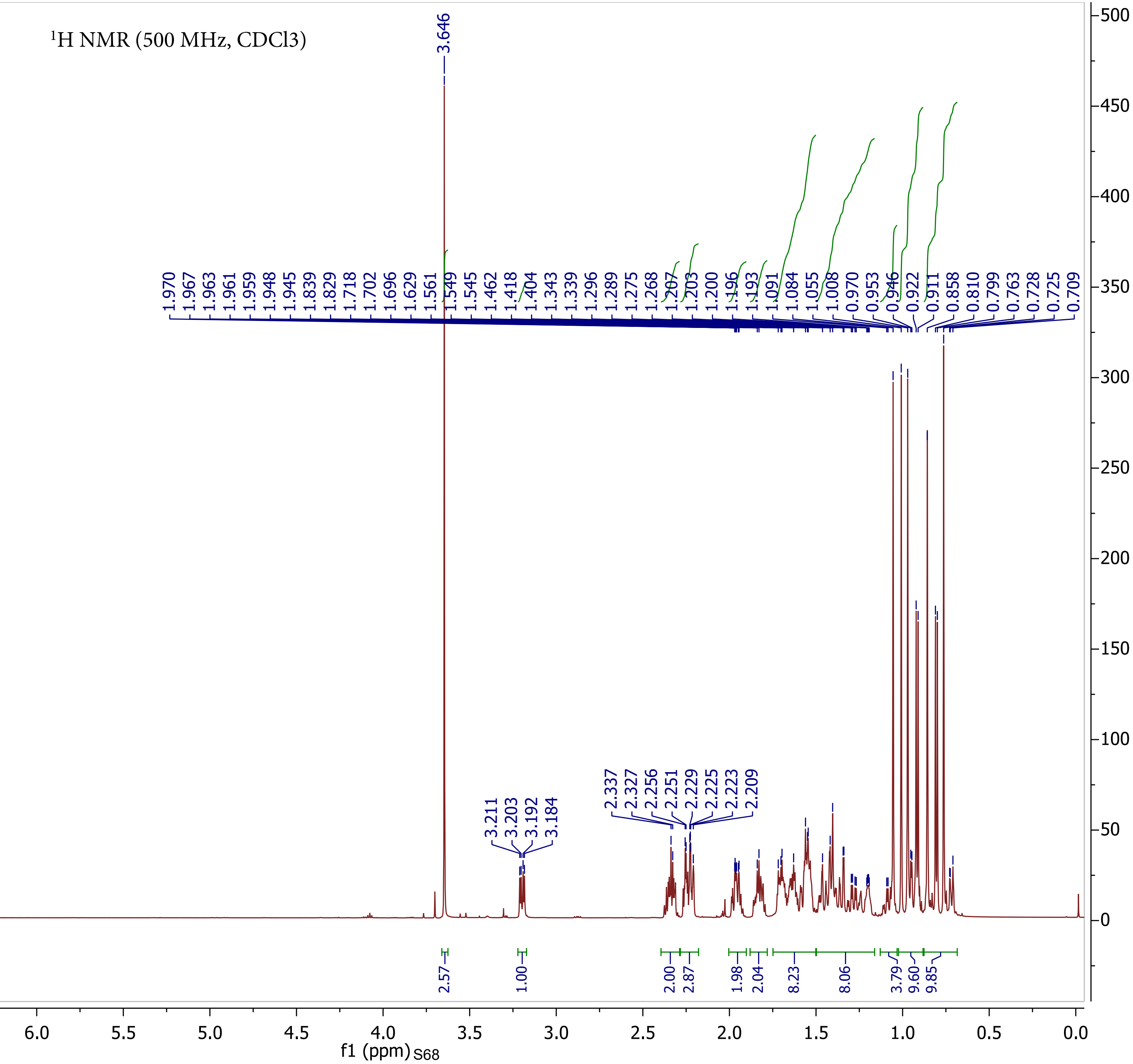

¹H NMR (500 MHz, CDCl3)
3.646
1.970
1.967
1.963
1.961
1.959
1.948
1.945
1.839
1.829
1.718
1.702
1.696
1.629
1.561
1.549
1.545
1.462
1.418
1.404
1.343
1.339
1.296
1.289
1.275
1.268
1.207
1.203
1.200
1.196
1.193
1.091
1.084
1.055
1.008
0.970
0.953
0.946
0.922
0.911
0.858
0.810
0.799
0.763
0.728
0.725
0.709
3.211
3.203
3.192
3.184
2.337
2.327
2.256
2.251
2.229
2.225
2.223
2.209
2.57
1.00
2.00
2.87
1.98
2.04
8.23
8.06
3.79
9.60
9.85
f1 (ppm)

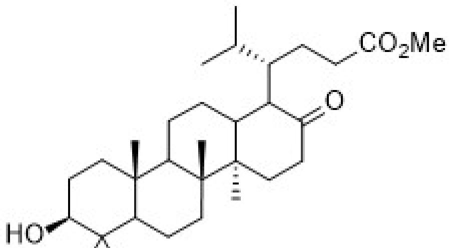

17

$^{13}C\{^1H\}$ NMR (125 MHz, CDCl3)

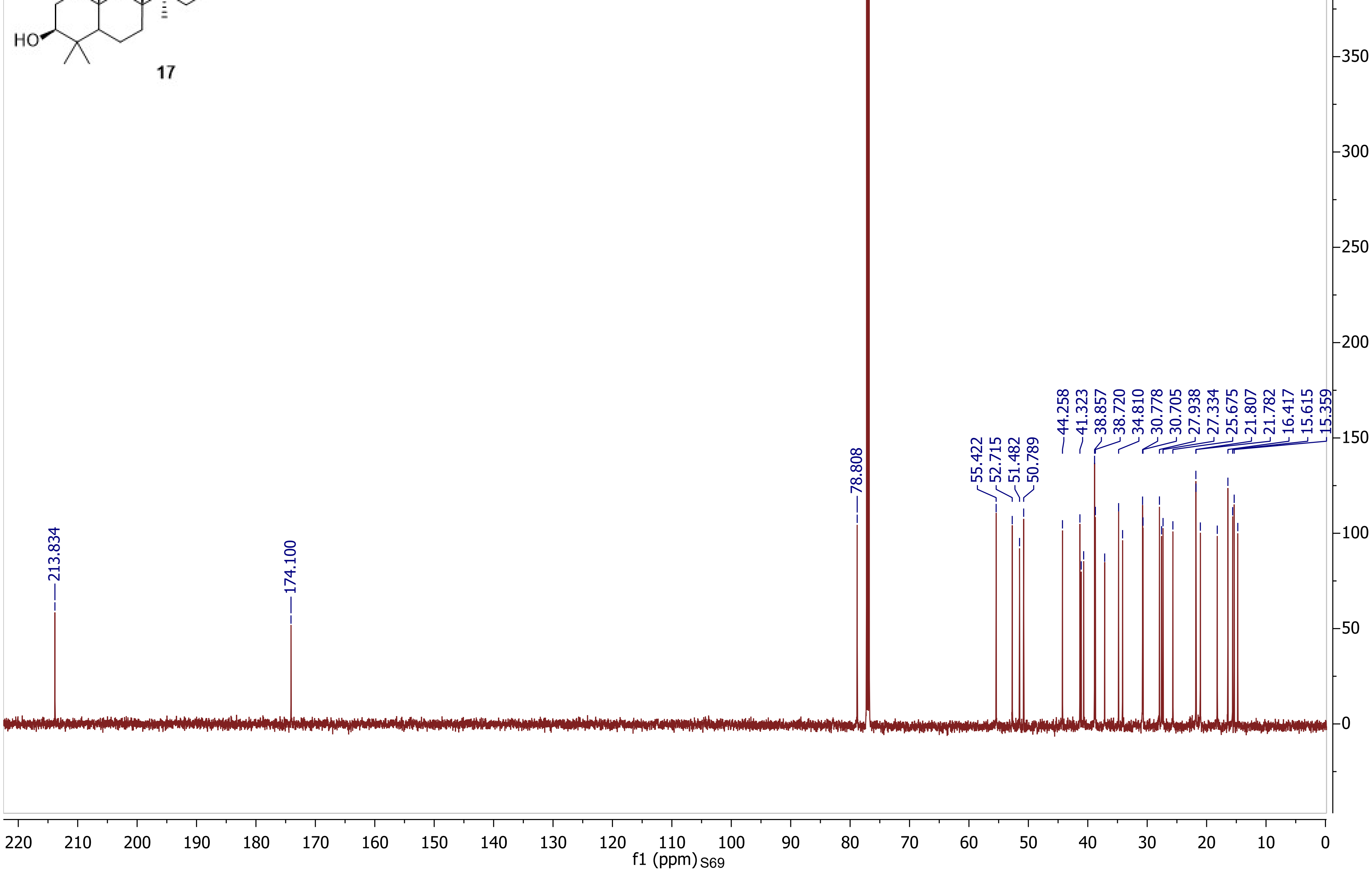

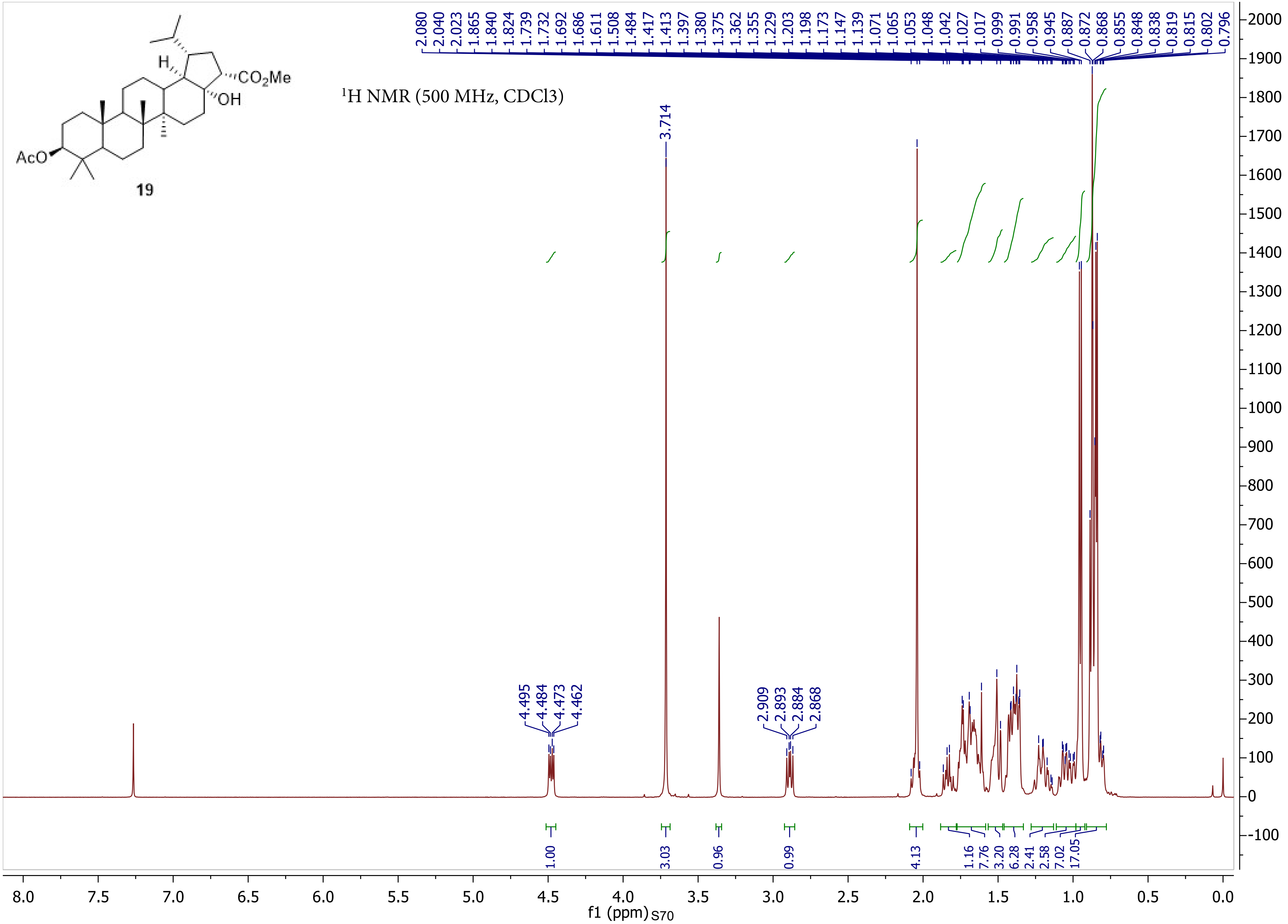

AcO
CO2Me
OH
H
19
1H NMR (500 MHz, CDCl3)
2.080 2.040 2.023 1.865 1.840 1.824 1.739 1.732 1.692 1.686 1.611 1.508 1.484 1.417 1.413 1.397 1.380 1.375 1.362 1.355 1.229 1.203 1.198 1.173 1.147 1.139 1.071 1.065 1.053 1.048 1.042 1.027 1.017 0.999 0.991 0.958 0.945 0.887 0.872 0.868 0.855 0.848 0.838 0.819 0.815 0.802 0.796
3.714
4.495 4.484 4.473 4.462
2.909 2.893 2.884 2.868
1.00 3.03 0.96 0.99 4.13 1.16 7.76 3.20 6.28 2.41 2.58 7.02 17.05
f1 (ppm)

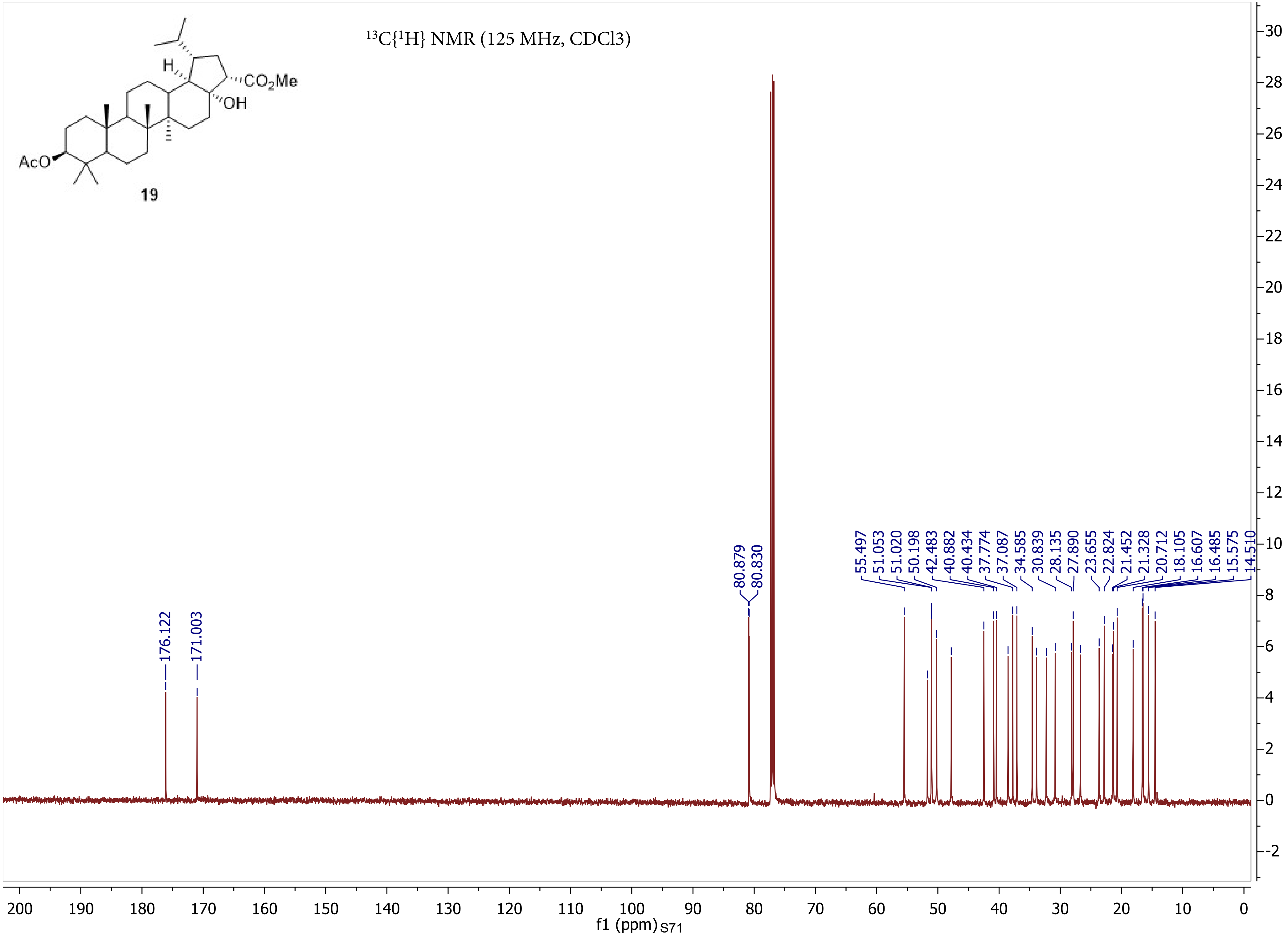

$^{13}C\{^1H\}$ NMR (125 MHz, CDCl3)
H
CO_2Me
OH
AcO
19
176.122
171.003
80.879
80.830
55.497
51.053
51.020
50.198
42.483
40.882
40.434
37.774
37.087
34.585
30.839
28.135
27.890
23.655
22.824
21.452
21.328
20.712
18.105
16.607
16.485
15.575
14.510
f1 (ppm)

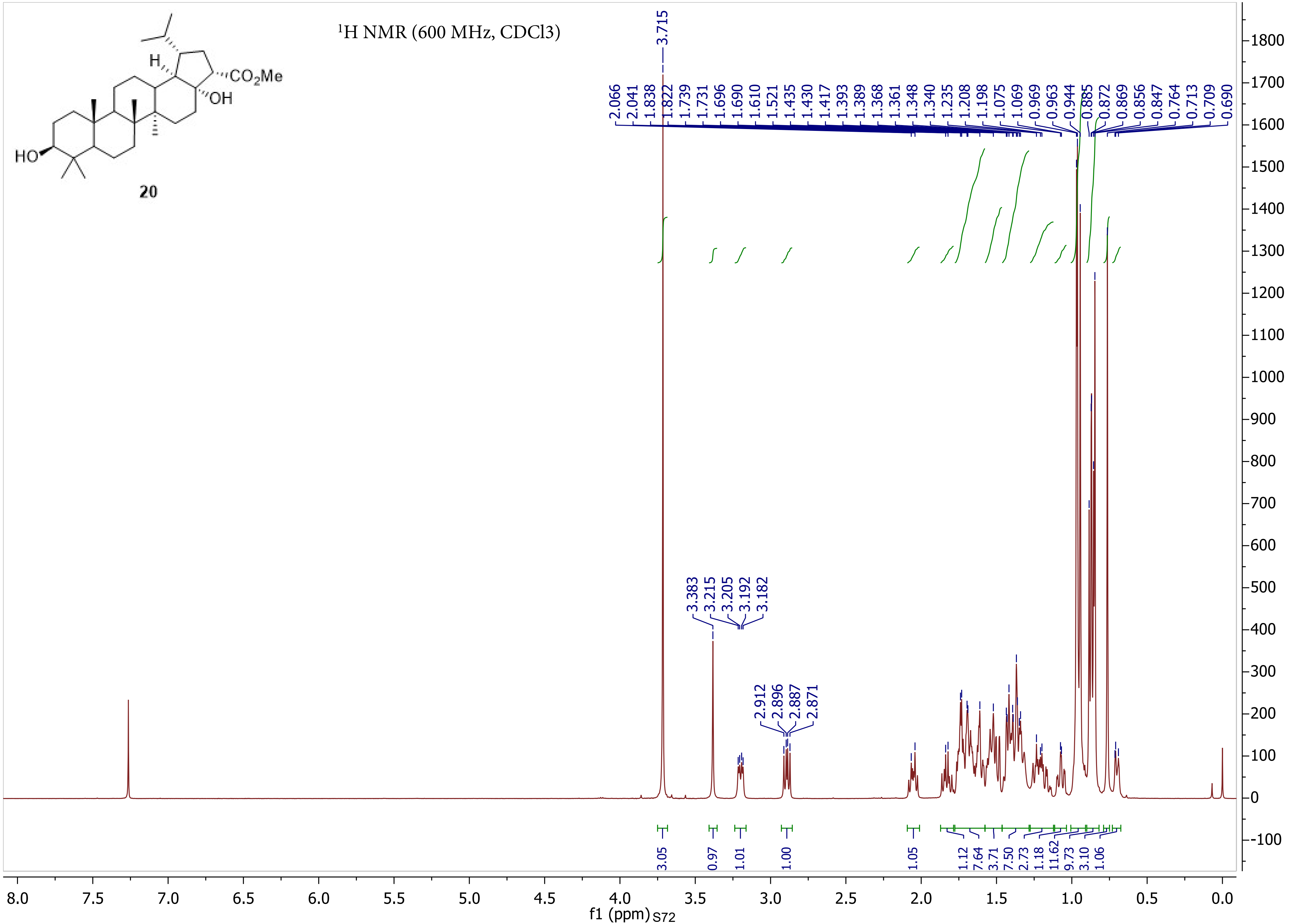

1H NMR (600 MHz, CDCl3)
20
3.715
2.066 2.041 1.838 1.822 1.739 1.731 1.696 1.690 1.610 1.521 1.435 1.430 1.417 1.393 1.389 1.368 1.361 1.348 1.340 1.235 1.208 1.198 1.075 1.069 0.969 0.963 0.944 0.885 0.872 0.869 0.856 0.847 0.764 0.713 0.709 0.690
3.383 3.215 3.205 3.192 3.182
2.912 2.896 2.887 2.871
3.05 0.97 1.01 1.00 1.05 1.12 7.64 3.71 7.50 2.73 1.18 11.62 9.73 3.10 1.06
f1 (ppm)

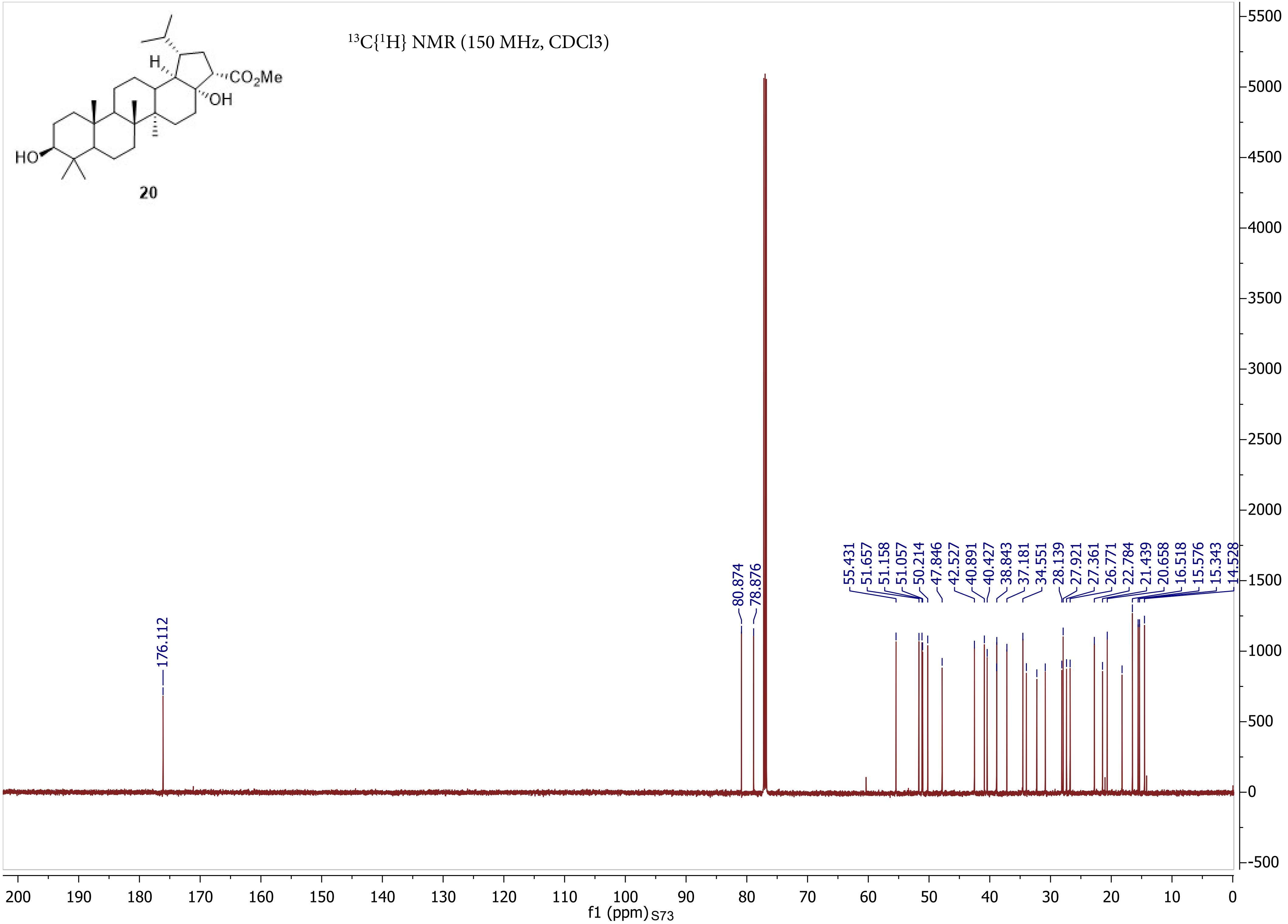

$^{13}C\{^1H\}$ NMR (150 MHz, CDCl3)
H
CO_2Me
OH
HO
20
176.112
80.874
78.876
55.431
51.657
51.158
51.057
50.214
47.846
42.527
40.891
40.427
38.843
37.181
34.551
28.139
27.921
27.361
26.771
22.784
21.439
20.658
16.518
15.576
15.343
14.528
f1 (ppm)

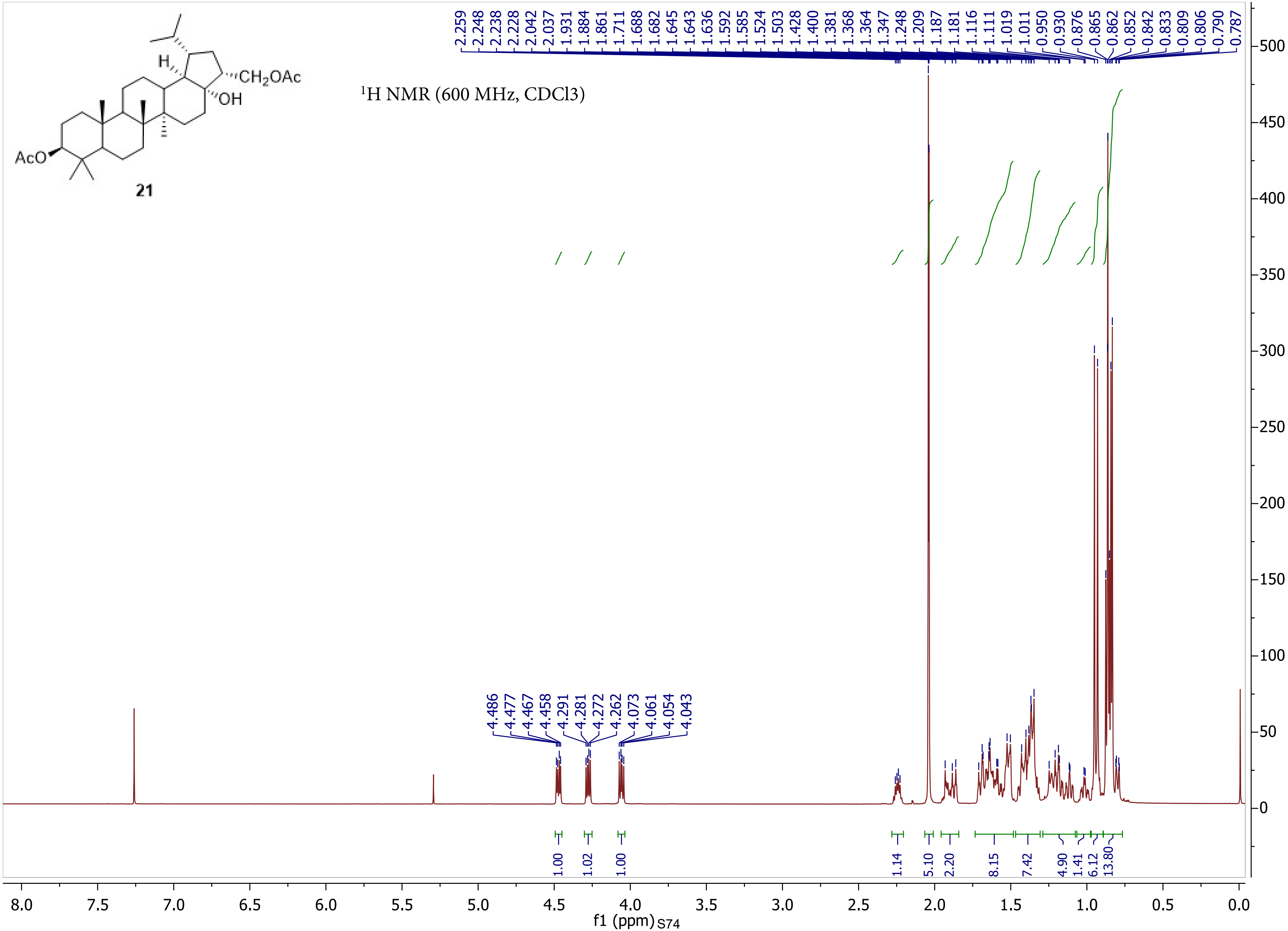

21

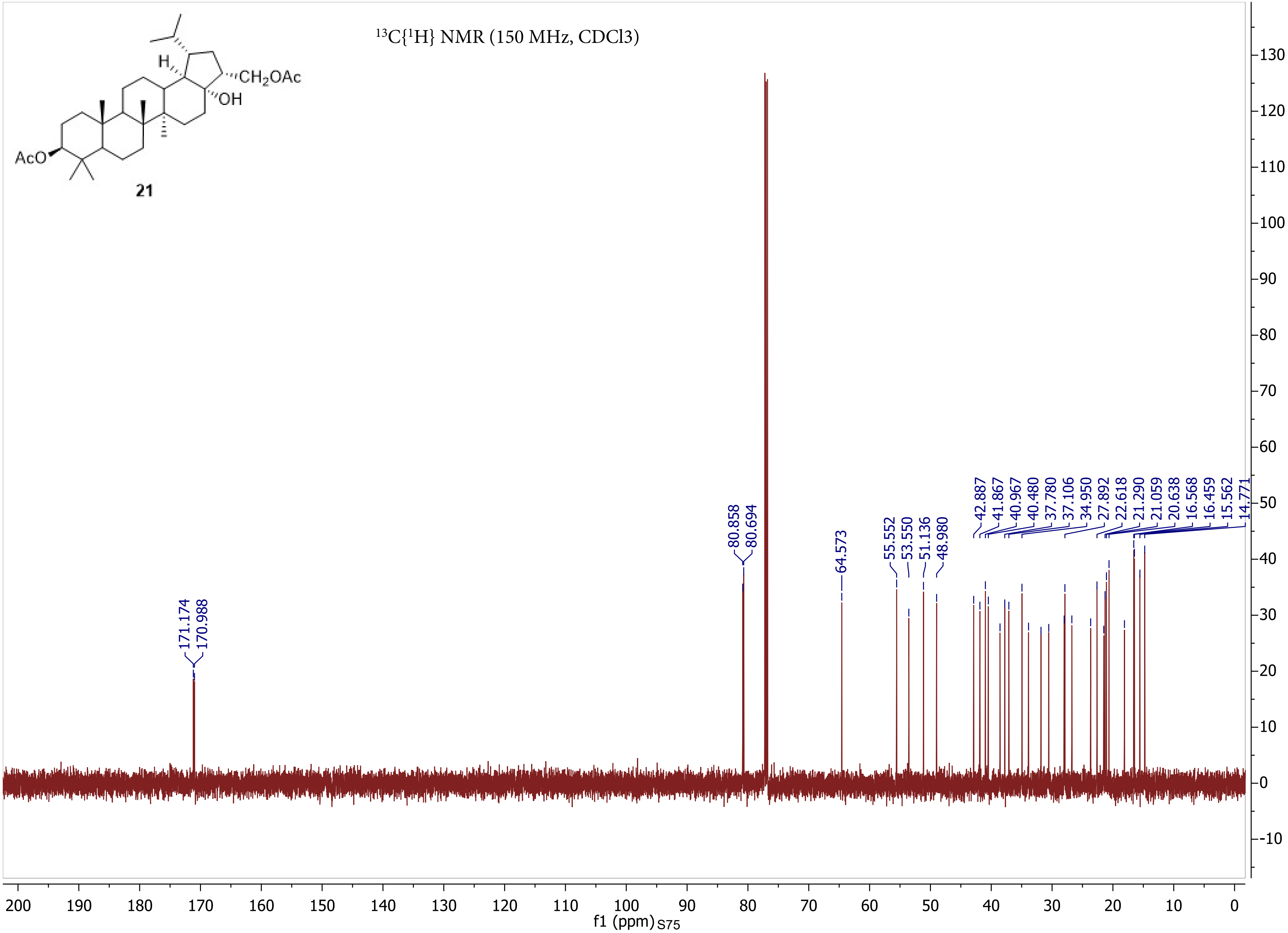

21
13C{1H} NMR (150 MHz, CDCl3)
171.174
170.988
80.858
80.694
64.573
55.552
53.550
51.136
48.980
42.887
41.867
40.967
40.480
37.780
37.106
34.950
27.892
22.618
21.290
21.059
20.638
16.568
16.459
15.562
14.771
f1 (ppm)

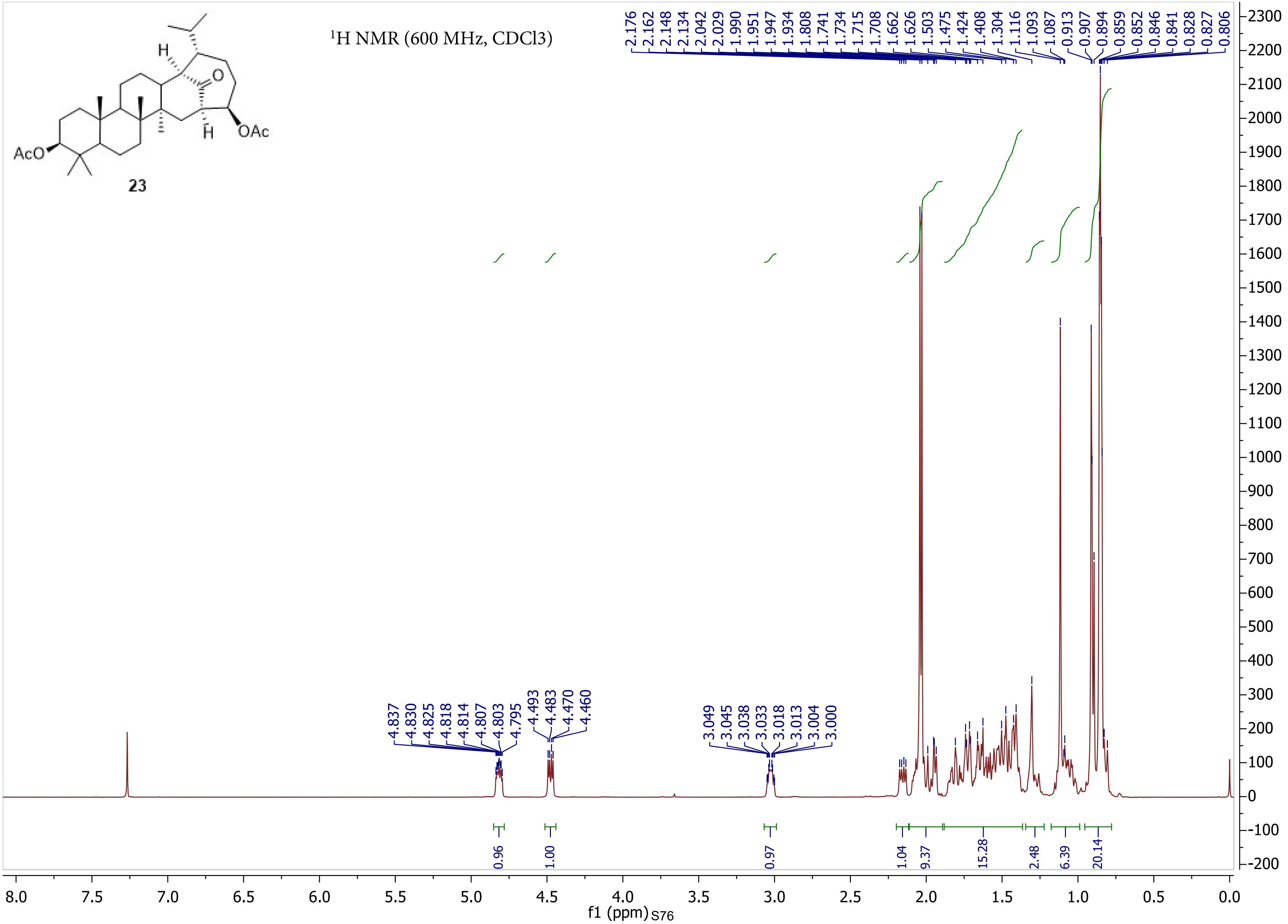
^{1}H NMR (600 MHz, CDCl3)
AcO
OAc
23
f1 (ppm)

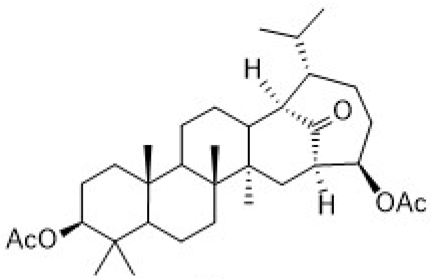
AcO
OAc
H
H
O

23

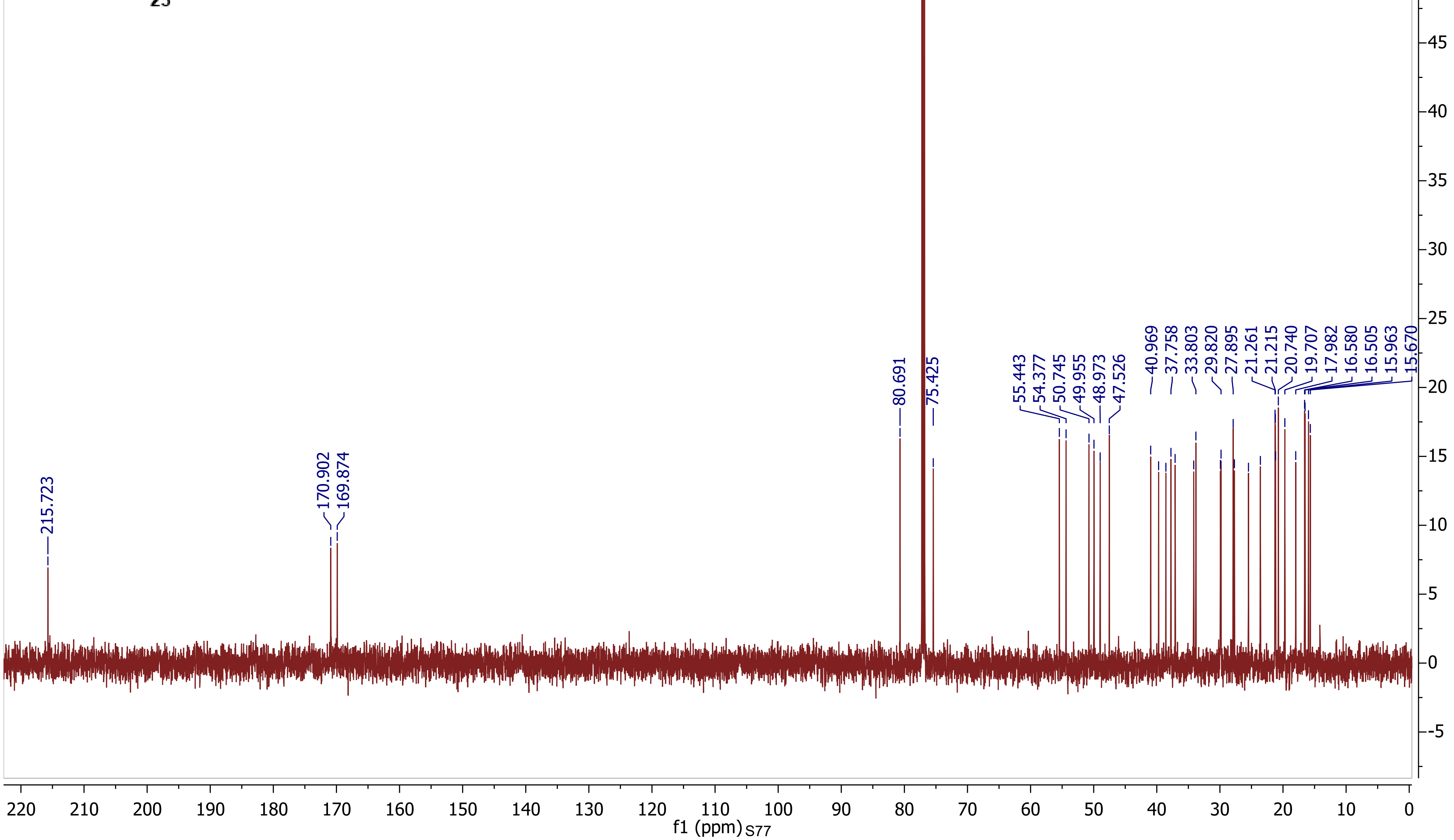
$^{13}C\{^1H\}$ NMR (150 MHz, CDCl3)
215.723
170.902
169.874
80.691
75.425
55.443
54.377
50.745
49.955
48.973
47.526
40.969
37.758
33.803
29.820
27.895
21.261
21.215
20.740
19.707
17.982
16.580
16.505
15.963
15.670
f1 (ppm)

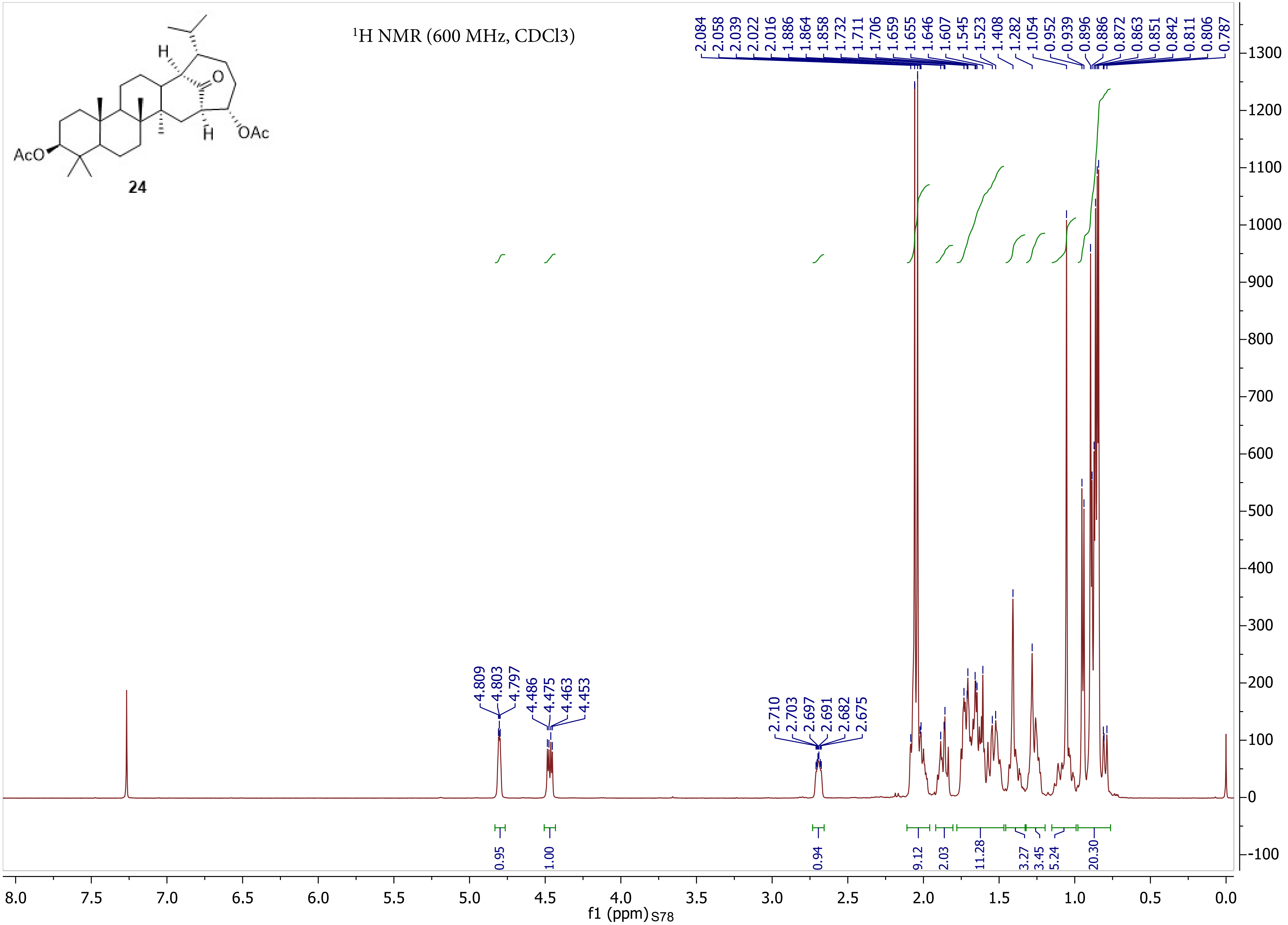

¹H NMR (600 MHz, CDCl3)
AcO
OAc
H
O
24
2.084 2.058 2.039 2.022 2.016 1.886 1.864 1.858 1.732 1.711 1.706 1.659 1.655 1.646 1.607 1.545 1.523 1.408 1.282 1.054 0.952 0.939 0.896 0.886 0.872 0.863 0.851 0.842 0.811 0.806 0.787
4.809 4.803 4.797
4.486 4.475 4.463 4.453
2.710 2.703 2.697 2.691 2.682 2.675
0.95
1.00
0.94
9.12
2.03
11.28
3.27
3.45
5.24
20.30
f1 (ppm)

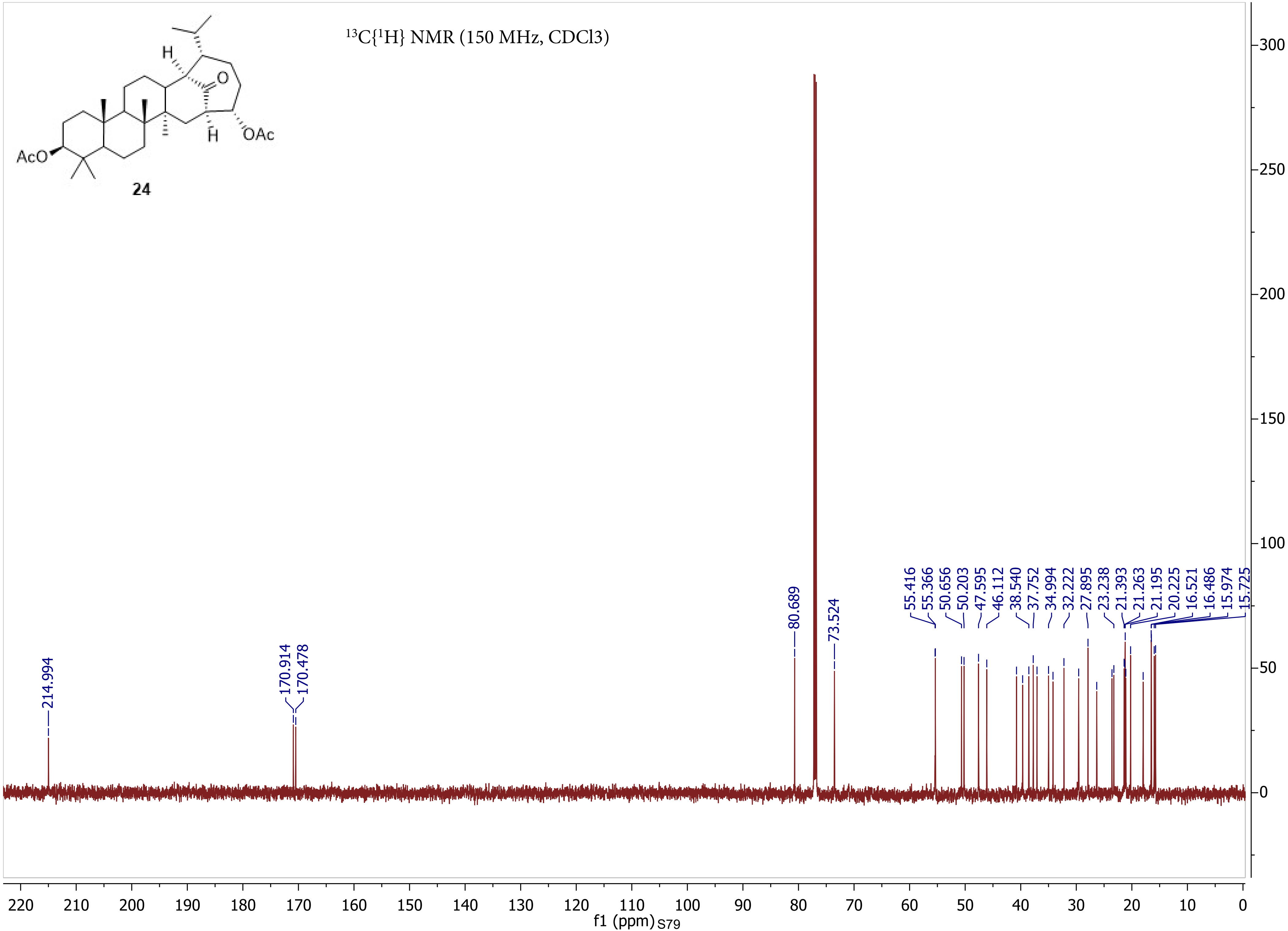

$^{13}C\{^1H\}$ NMR (150 MHz, CDCl3)
AcO
OAc
H
H
O
24
214.994
170.914
170.478
80.689
73.524
55.416
55.366
50.656
50.203
47.595
46.112
38.540
37.752
34.994
32.222
27.895
23.238
21.393
21.263
21.195
20.225
16.521
16.486
15.974
15.725
f1 (ppm)
